# Mass Spectrometric Detection of Formaldehyde-Crosslinked PBMC Proteins in Cell-Free DNA Blood Collection Tubes

**DOI:** 10.3390/molecules28237880

**Published:** 2023-11-30

**Authors:** Daniel Röth, Jessica Molina-Franky, John C. Williams, Markus Kalkum

**Affiliations:** 1 Department of Immunology and Theranostics, Arthur Riggs Diabetes and Metabolism Research Institute, Beckman Research Institute of the City of Hope, Duarte, CA 91010, USAjemolina@coh.org (J.M.-F.); 2 Molecular Biology and Immunology Department, Fundación Instituto de Inmunología de Colombia (FIDIC), Bogotá 112111, Colombia; 3 Biotechnology Institute Department, Faculty of Sciences, Universidad Nacional de Colombia, Bogotá 111321, Colombia; 4 Department of Cancer Biology and Molecular Medicine, Beckman Research Institute of the City of Hope, Duarte, CA 91010, USA

**Keywords:** formaldehyde, Streck tubes, peripheral blood mononuclear cells, crosslinking, targeted mass spectrometry

## Abstract

Streck tubes are commonly used to collect blood samples to preserve cell-free circulating DNA. They contain imidazolidinyl urea as a formaldehyde-releasing agent to stabilize cells. We investigated whether the released formaldehyde leads to crosslinking of intracellular proteins. Therefore, we employed a shotgun proteomics experiment on human peripheral blood mononuclear cells (PBMCs) that were isolated from blood collected in Streck tubes, EDTA tubes, EDTA tubes containing formaldehyde, or EDTA tubes containing allantoin. The identified crosslinks were validated in parallel reaction monitoring LC/MS experiments. In total, we identified and validated 45 formaldehyde crosslinks in PBMCs from Streck tubes, which were also found in PBMCs from formaldehyde-treated blood, but not in EDTA- or allantoin-treated samples. Most were derived from cytoskeletal proteins and histones, indicating the ability of Streck tubes to fix cells. In addition, we confirm a previous observation that formaldehyde crosslinking of proteins induces a +24 Da mass shift more frequently than a +12 Da shift. The crosslinking capacity of Streck tubes needs to be considered when selecting blood-collection tubes for mass-spectrometry-based proteomics or metabolomic experiments.

## 1. Introduction

The goal of this study was to determine if measurable formaldehyde crosslinking occurs in proteins of white blood cells (here PBMCs) after blood collection with Streck cell-free DNA blood-collection tubes (cfDNA BCTs). These tubes are designed to obtain cell-free circulating DNA for liquid biopsy testing, for example, for the identification of tumor mutations in blood samples, while avoiding contamination by cellular DNA of lysed white blood cells [1]. Streck’s cfDNA BCTs utilize imidazolidinyl urea (IDU), a formaldehyde-releasing agent, that facilitates the analysis of cfDNA by preserving intact cells and preventing cellular DNA release, without causing damage to cfDNA that would otherwise impair sequencing [1,2]. Additionally, Streck cfDNA BCTs contain glycine, a quenching agent, which can react with formaldehyde and render it inactive [2]. However, despite the tubes’ widespread use, the extent to which formaldehyde released in Streck’s cfDNA BCTs is capable of penetrating and crosslinking intracellular proteins has not yet been investigated. Knowledge of the extent of crosslinking matters for the potential use of Streck’s cfDNA BCTs for mass-spectrometry-based metabolomics and proteomics applications.

To explore this question, we employed mass spectrometry to investigate formaldehyde crosslinking in PBMCs from whole blood collected in Streck cfDNA BCTs. Although the use of formaldehyde as a fixative agent is well-documented, and several mass spectrometry studies have investigated formaldehyde as a crosslinking agent [3,4,5], an important discovery was recently published by the Kalisman group [6]. The group observed that the mass-spectrometric analysis of formaldehyde crosslinking between proteins predominantly produces a +24 Da mass shift rather than the expected +12 Da mass shift. Importantly, this +24 Da mass shift was not observed when reacting unstructured peptides with formaldehyde. The exact chemical nature of the +24 Da mass shift is reportedly under investigation [6].

**Figure 1:**
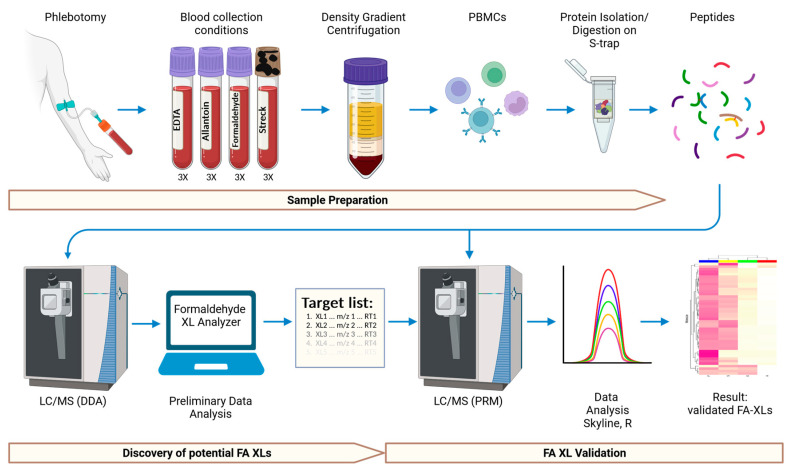
Workflow. Schematics of sample preparation, mass spectrometry, and data analysis for the identification of Streck-tube-induced formaldehyde crosslinking in PBMC proteins. Created with BioRender.com.

Using data-dependent acquisition (DDA), we carried out a shotgun mass spectrometry approach to identify and compare protein crosslinks formed in PBMCs from the collection of whole blood in Streck cfDNA BCTs, in EDTA tubes with no additional additive, and in EDTA tubes spiked with formaldehyde or allantoin, a known degradation product of IDU. We further validated crosslinked proteins using a parallel reaction monitoring (PRM) targeted mass spectrometry approach. The resulting data showed substantial formaldehyde crosslinks between intracellular proteins of PMBCs collected in Streck cfDNA BCTs (Figure 1).

## 2. Results

Blood samples were collected from three healthy human donors. For each donor, we collected one Streck 10 mL cfDNA BCT (S sample) and three 10 mL EDTA tubes. Out of those, one EDTA tube was treated with formaldehyde (F sample), or allantoin (A sample), or was left untreated (E sample). After treatment, the collection tubes were stored for approximately 24 h at room temperature to simulate real-life use and transport conditions, and the PBMCs were subsequently isolated.

Trypsin-derived peptides from the proteins extracted from the 12 PBMC samples (from 4 tubes × 3 donors) were analyzed by nano LC/MS in a data-dependent acquisition (DDA) mode (Figure 1). Since crosslinked peptides are expected to be larger and to contain more residues than most tryptic peptides, only precursor ions with charge states of 4+ to 8+ were selected for fragmentation. The resulting MS/MS spectra were processed and analyzed using the Kalisman group’s Formaldehyde_XL_Analyzer software, which identifies potential formaldehyde crosslinks with mass shifts of +24 or +12 Da [6]. To minimize false assignments and noise, only spectra that were unique to each of the Streck, formaldehyde, EDTA and allantoin samples, were used. Potential crosslinks with a ratio of the number of peptide fragment ions detected over the total length of the crosslinked peptides greater than 1.5 were considered as high-confidence potential crosslinks, and were kept as recommended by the instructions for use of the Formaldehyde_XL_Analyzer [6].

Given the complexity of whole-blood samples and the stochastic nature of shotgun proteomics (DDA), we sought to further validate the peptide crosslinks identified by the Formaldehyde_XL_Analyzer software. Focusing on potential crosslinks in the S samples, we obtained 116 precursor *m*/*z* value and retention time pairs of potential peptide crosslinks to serve as targets in PRM validation experiments, which allowed for the comparison of all samples in an unbiased and non-stochastic manner. To accommodate potential shifts in retention time and to observe the level of background noise signals, we employed a six-minute window centered around the average retention time at which each target precursor ion was originally identified. PRM was performed a total of 1392 times (116 peptide crosslink precursor ions × 12 samples). The resulting PRM LC/MS/MS data were analyzed with Skyline and R scripts and subjected to strict and conservative filtering. First, the expected transitions were precalculated for each crosslinked peptide pair using R and then matched to the PRM data by accepting only transitions to b and y ion numbers > 4, since smaller b or y ions can also originate from irrelevant non-crosslinked co-isolating peptide ions that were often seen in the extracted ion chromatograms (discussed below). Second, only crosslinks between peptides each at least seven residues long were retained, as shorter peptide sequences are more difficult to unambiguously assign to a protein. Third, ion chromatograms that had low intensities below 3000 counts (noise level) or deviated more than 1 ppm from the expected fragment ion masses were excluded. Finally, a crosslinked peptide precursor ion was only accepted if five or more expected transitions were observed in at least one sample. After applying these strict parameters, 57 crosslinked peptide precursor ions remained. The validation strategy ensured that any extract ion chromatogram reflected the presence of the targeted peptide crosslink and not that of an unrelated co-isolation peptide ion.

**Figure 2:**
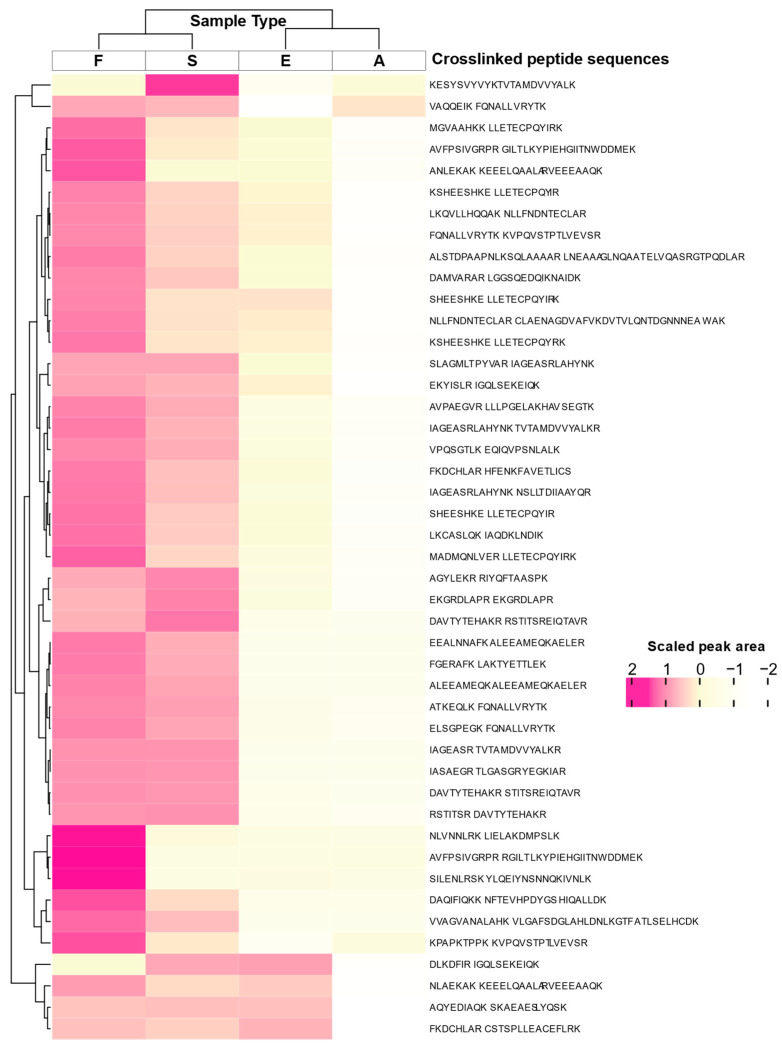
Validated crosslinks, heatmap. The scaled peak area is presented for averaged total transition intensities of crosslinked peptides from PBMC samples in BCTs with formaldehyde (F), Streck tubes (S), EDTA- (E), and allantoin-treated (A) tubes.

For all the 12 samples and the 57 crosslinked peptide precursor PRM experiments, extracted ion chromatograms were assembled. For each chromatogram, the intensity of each observed transition over the course of the observation window was displayed as a different colored trace (Supplementary Materials S2). Next, the area under the curve of all the transition signal intensities in each extracted ion chromatogram was averaged across the three donors. Where MS/MS data were obtained for multiple charged states of the same crosslinked pair, these results were combined, resulting in 45 distinct formaldehyde-crosslinked peptide pairs (from 57 peptide precursor ions, Figure 2). As shown in Figure 2, there was a high degree of similarity between the signal intensities of the crosslinked peptides from the S and F samples, as well as similarity between the E and A sample types, with E and A appearing weaker than the F and S intensities.

**Figure 3:**
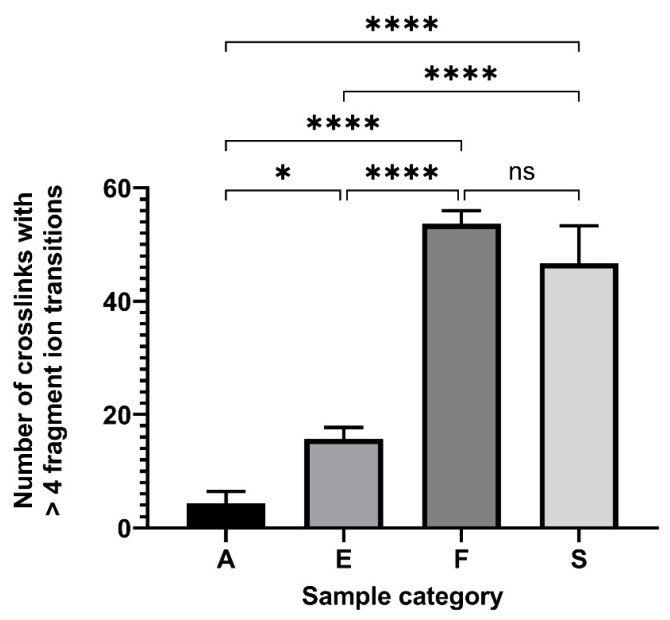
Number of validated crosslinked peptides with at least 5 precursor-to-fragment ion transitions. Significance was determined by one-way ANOVA with Tukey’s multiple comparisons tests. *p* ≤ 0.05 (*), *p* ≤ 0.0001 (****), not significant (ns).

Statistical analysis of validated crosslinked peptide ions with at least five fragment ion transitions (Figure 3) showed that the number of crosslinks between those obtained from Streck cfDNA BCTs and EDTA tubes with and without allantoin treatment was significantly different (sample categories S versus E or A). The same was true when crosslinks from formaldehyde-treated EDTA tubes (sample category F) were compared to those of allantoin-treated or untreated EDTA tube samples (F versus E or A). There was no statistical difference between the number of validated crosslinks in formaldehyde-treated (F) and Streck-tube-collected (S) samples.

Of the 45 validated formaldehyde-crosslinked peptides identified (Figure 2), 14 (approximately 31%) had the +12 Da mass shift and 31 (approximately 69%) had the +24 Da mass shift (Table 1). These results are comparable with those reported by Tary-Wilk et al., which determined that approximately 25% of formaldehyde crosslinks create a +12 Da mass shift and 75% of formaldehyde crosslinks create a +24 Da mass shift [6].

The 45 validated peptide pairs comprised 68 distinct peptides as some peptides formed formaldehyde crosslinks with more than one other peptide (Table 1). Of those 68 peptides, 67 matched exactly one protein in the human protein database (Table 2). The remaining peptide, AVFPSIVGRPR, which was formaldehyde-crosslinked to the protein ACTH_HUMAN (actin, gamma), occurs in both the protein POTEF_HUMAN (POTE ankyrin domain family member F) and the protein ACTB_HUMAN (actin, cytoplasmic 1) (Supplementary Materials S1). Regardless of which protein the peptide sequence AVFPSIVGRPR formaldehyde crosslinked, the PRM confirmed that ACTH_HUMAN interacted with one or the other or both.

**Figure 4:**
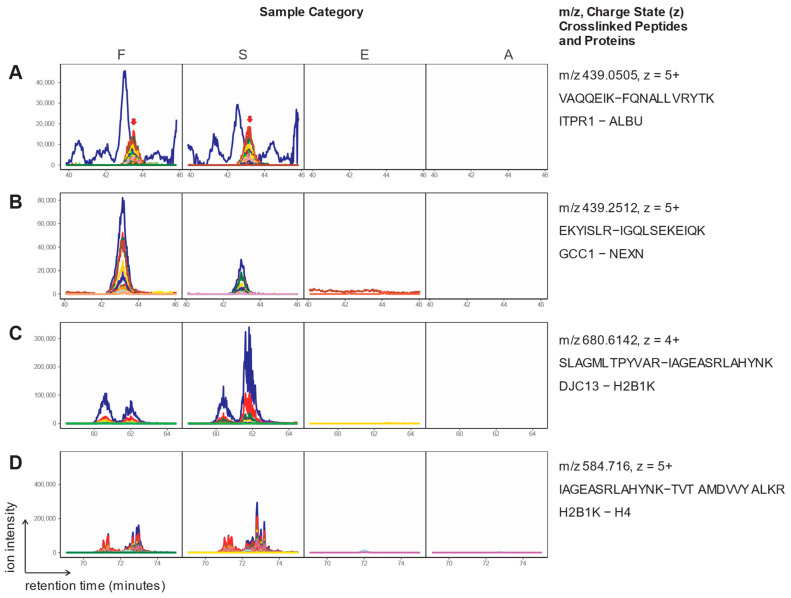
Examples of extracted ion chromatograms of precursor-to-fragment ion transitions of formaldehyde-crosslinked peptides. Each colored curve represents the extracted ion chromatogram of a transition (b or y ions) matching the indicated peptide crosslink. Red arrows in A mark the position of the multi-transition peak of the validated crosslink, while the other green and dark blue lines stem from co-isolating background ions.

Evaluation of the extracted ion chromatograms of the crosslinked peptides in the validation experiments revealed interesting details on the heterogeneous nature of formaldehyde crosslinking. In several cases a single chromatographic peak of several transitions was observed at the previously identified average retention times (Figure 4A,B). However, in most cases there were multiple chromatographic peaks for precursors of the same *m*/*z* and transitions. Often those signals appeared as two clearly separated peaks with distinct retention times (Figure 4C). In other cases, they appeared as shoulders or a series of multiple peaks (Figure 4D). The presence of interfering non-relevant peptide ions in the PRM data is demonstrated in Figure 4A. The blue chromatograms likely stem from other peptide ions that are not parts of the overlaid, multicolored, chromatographic peaks of the targeted crosslink. The *m*/*z* of the precursors shown in Figure 4A,B fell within the 0.7 Da isolation window used and had very similar retention times. This case demonstrates why strict filtering of the PRM data was necessary for the validation.

## 3. Discussion

The value of PBMCs for proteomic studies to investigate disease biomarkers has previously been discussed [7]. Our study demonstrates conclusively that formaldehyde crosslinking occurs between proteins in PBMCs from blood samples collected in Streck tubes. This is an important finding that should be considered when choosing blood-collection tubes for proteomics or metabolomic experiments [8,9]. We confirm the findings by the Kalisman group, that a +24 Da mass shift occurs more frequently in formaldehyde-crosslinked peptides from structural proteins than a +12 Da shift [6]. In addition, our study analyzed the intensity of crosslinks, providing a relative quantitative comparison of crosslinking between blood-collection conditions. Most formaldehyde crosslinks were observed in abundant structural proteins, including histones and cytoskeletal proteins, including myosin, actin, talin, and keratin (Table 2). Beyond finding this subset of the same crosslinked proteins reported in Kalisman, we also observed several novel intermolecular crosslinks. For instance, Indian hedgehog (IHH, crosslinked to the G-protein coupled receptor, ORJ10J4) has been shown to be expressed in CD4^+^CD8^+^ double-positive cells and plays a critical role in the regulation of thymocyte differentiation [10]. It was also reported to be expressed in macrophages [11], and has been quantified as a secreted protein in blood [12]. MEGF11, a protein most often associated with epithelial cells, was previously reported to be expressed in PBMCs at the mRNA level [13]. It plays a critical role in white blood cells as a single nucleotide polymorphism (SNP) in the MEGF11 gene, which is strongly associated with the development of Hodgkin lymphoma [14].

We also detected three intramolecular crosslinks within albumin, and three other crosslinks between albumin and the proteins DOK2, MYLK, and ITPR1. Similarly, we detected crosslinking between the β and δ subunits of hemoglobin. Despite stringent washes of the PBMCs, albumin was detected and validated in the protein crosslinks from lysed cells. This is an expected finding since albumin is the most abundant protein in blood and is known to become internalized under certain conditions. While it is not surprising that we and others detected formaldehyde crosslinking of histones, a set of highly abundant, positively charged proteins responsible for condensing genomic DNA, we posit that numerous proteins of low abundance are also crosslinked. Critically, our validated crosslink data indicate that formaldehyde, released from the Streck tubes, penetrates white blood cells and crosslinks proteins throughout the cell (e.g., histones largely residing in the nucleus).

## 4. Materials and Methods

### 4.1. Chemicals and Reagents

Chemicals used were purchased from Sigma-Aldrich (St. Louis, MI, USA) or Fisher Scientific (Waltham, MA, USA) unless stated otherwise.

### 4.2. Collection and Processing of Blood Samples

Four tubes of blood (one in a 10 mL Streck cell-free DNA BCT tube (“Streck Tube”), and three in 10-mL EDTA Vacutainer tubes (BD)) were collected from each of three volunteers under an approved protocol by the Institutional Review Board of the City of Hope National Medical Center. Collections were performed in accordance with the tube manufacturers’ instructions (https://www.streck.com/wp-content/uploads/sync/Stabilization/Cell-Free_DNA_BCT_RUO_CE/01_Instructions_(IFU)/01_Cell-Free_DNA_BCT_RUO_IFU.pdf last accessed on 29 November 2023; http://static.bd.com/documents/eifu/VDP40161_ENv10.pdf; last accessed on 29 November 2023). Two of the three EDTA tube samples from each donor were further processed after one hour of the venous blood draw. For each donor, 200 µL of an aqueous solution containing 30% allantoin was mixed into one of the EDTA tube samples, and 225 µL of a 10% neutral buffered solution containing formaldehyde (4% weight per volume) was added as a positive control to the other EDTA tube sample, resulting in a final formaldehyde concentration of 0.1%. As a negative control, the remaining EDTA tube sample underwent no further processing. In total, we obtained a total of three Streck tube samples, three Allantoin samples, three formaldehyde samples, and three untreated EDTA tube samples. The twelve tubes were mixed by multiple slow inversions and left to stand for approximately 24 h before processing began. 

### 4.3. Isolation of Human Peripheral Blood Mononuclear Cells

Each blood sample was diluted 1:1 with phosphate-buffered saline (PBS) by gently mixing 10 mL whole blood with 10 mL PBS by pipetting it up and down. The peripheral blood mononuclear cells (PBMCs) were isolated using two SepMate-50 (StemCell, Cambridge, MA) tubes according to the manufacturer’s instructions and centrifuged at room temperature at 760× *g* for 20 min. The enriched PBMCs from the top layer were poured into a fresh tube, and washed with 20 mL PBS by centrifugation at 760× *g* for 10 min. After carefully removing the supernatants without disturbing the pellets, the two fractions from each sample were combined and mixed by pipetting up and down. Finally, the combined cells were washed with PBS and centrifuged at 760× *g* for 10 min.

### 4.4. Lysis of the Peripheral Blood Mononuclear Cells

Each cell pellet was resuspended in 1.4 mL SDT-lysis buffer (4% SDS, 100 mM Tris/HCl pH 7.6, 0.1 M DTT) [15]. The samples were incubated at 37 °C for 20 min. The viscosity of the samples was subsequently decreased by shearing the DNA using a 25-gauge syringe needle and the PBMC lysates were clarified by centrifugation at 16,000× *g* for 5 min. After lysis, the protein cell lysates were evaluated by SDS-polyacrylamide gel electrophoresis, and the protein concentration was determined by densitometry using a BSA standard and the ImageJ program v1.53s of the National Institutes of Health. 

### 4.5. Protein Preparation for Mass Spectrometry

Samples of 100 µg protein each were adjusted to 5% SDS. Proteins were alkylated by adding 210 mM iodoacetamide and then incubated at room temperature in the dark for 15 min. Proteins were precipitated and washed on S-trap mini-columns according to the instructions by the manufacturer (ProtiFi, S-trap mini protocol 4.1, https://files.protifi.com/protocols/s-trap-mini-long-4-1.pdf; accessed on 21 March 2023). Subsequently, the proteins were digested on the S-trap columns with 10 µg trypsin/lys-C each at 25 °C overnight. Peptides were eluted following the manufacturer’s protocol and evaporated to dryness using a speed vac.

### 4.6. Liquid Chromatography—Mass Spectrometry (LC-MS)

The digested peptides were analyzed on an Exploris 480 orbitrap mass spectrometer equipped with a 1200 Easy nanoLC system using an Acclaim PepMap 100 pre-column (75 µm × 2 cm, nano Viper 2Pk C18, 3 µm, 100 Å) with a PepMap RSLC C18 analytical reversed-phase column (75 µm × 25 cm, 2 µm, 100 Å). A 190-min-long acetonitrile/water gradient with 0.1% formic acid was applied at 300 nL/min as follows: Buffer A consisted of 0.1% formic acid in water; Buffer B was 80% acetonitrile with 0.1% formic acid, the rest water. Gradient: 0 min at 5% B, 175 min to 45% B, 180 min to 100% B, and 190 min at 100% B. Before each analytical gradient the columns were equilibrated, and at least one blank was run after each sample. Before and after each nLC/MS experiment, BSA digest (Thermo, Waltham, MA, USA) was used to verify the proper performance of the nLC/MS system.

For the mass-spectrometric discovery of crosslinks, all samples were first analyzed in data-dependent acquisition mode using the following parameters: ion positive mode polarization with a spray voltage of 1800 V, static gas mode, an ion transfer tube temperature of 275 °C, and EASY-IC internal mass calibration set to on. For MS1 experiments, a scan range of *m*/*z* 400–1800 was set at 60,000 resolution, with an RF lens at 50%; the automatic gain control (AGC) target was set to “standard” with automatic maximum injection time mode. MS2 experiments used filters for monoisotopic precursor ion selection (MIPS) in peptide mode, with “relaxed restrictions when too few precursors found” set to False. For MS2 scans, the intensity threshold was set at a minimum of 1000, with an isolation width of *m*/*z* 0.7, at an orbitrap resolution of 60,000. The normalized AGC target was set to 200% with a maximum injection time of 100 ms. Only charge states from 4+ to 8+ were selected for MS2 fragmentation. Peptide ion charge states from 4+ to 6+ were fragmented at a normalized HCD collision energy of 28%, while charge states above 6+ were fragmented at 38% collision energy.

In addition, to determine which proteins were present in the PBMC samples, regardless of crosslink status, aliquots of the digest peptides of the EDTA tubes (E samples) were also analyzed in comparable DDA experiments that included charge states 2+ to 8+.

### 4.7. Data Processing and Analysis

The LC/MS data of the E samples were analyzed with MSFragger in FragPipe v19 using the default settings and the canonical human uniport protein sequence database. Briefly, the key parameters were: enzymatic digestion with trypsin, maximum of 2 missed cleavages, variable modifications for methionine oxidation and N-terminal acetylation, fixed modification for cysteine carbamidomethylation. Protein Prophet (FragPipe) settings included a protein false discovery rate of 0.01. The resulting list of identified protein IDs was used to retrieve a FASTA file of amino acid sequences for each protein from the UniProt web portal, which served as a database for identified proteins.

The LC/MS data raw files of the E, S, F, and A samples were converted from RAW to MGF format using MSConvert (ProteoWizard toolkit, v3.0.21101) and filtered using a custom python script to generate MGF files of spectra unique to each of the Streck, formaldehyde, allantoin, and EDTA samples. In addition, precursor masses >8000 Da were filtered out. The resulting data were then analyzed with the Formaldehyde_XL_Analyzer 64bit java program by the Nir Kalisman laboratory using the database comprising the identified proteins mentioned above plus the membrane proteins designated in the Human Protein Atlas database and all membrane proteins extracted from UniProt. Default settings with the FDR (15% of sequences) were used. Further data analysis was performed in R and the one-way ANOVA with Tukey’s multiple comparisons tests was performed in Prism V. 8 (GraphPad).

### 4.8. Validation of Formaldehyde Protein Crosslinks Using Parallel Reaction Monitoring (PRM)

We conducted a targeted LC/MS PRM experiment by using a list of the top-ranking FAXL precursor ions as targets (ranking according to the FAXL Finder’s ratio number of fragments/total length) and their retention times at the center of a 6 min retention time window. The list contained 116 PRM targets and was spread over a 190 min LC/MS gradient with identical columns and flow conditions as used for the original discovery experiments (see above). The resulting data were analyzed with Skyline software (MacCoss Lab, v22.2.0.527) against a list of calculated transitions for b and y ions for each crosslinked peptide and charge state with and without the +12 Da formaldehyde modification mass shifts. Fragment b and y ions with less than 3 residues were omitted since they are typically not uniquely sequence specific. Precursor peptides shorter than 7 amino acids were also excluded as they are less likely to be unambiguously matched to a single protein. The skyline output was further analyzed with R scripts to remove extracted ion chromatograms that had zero to 3000 count low intensities (noise level) or deviated more than 1 ppm from the expected fragment ion masses. Because a single transition in a chromatogram can represent noise rather than a true signal, a crosslinked peptide precursor ion was only accepted if five or more transitions were observed in at least one sample. The resulting data were assembled into a heatmap using the tidyHeatmap R package v1.8.1 [16].

## 5. Conclusions

Our analysis, which employs controls and additional validation measures, shows that proteins in PBMCs collected in Streck cfDNA BCTs are crosslinked by formaldehyde. Furthermore, we show that the released formaldehyde in Streck tubes is capable of penetrating white blood cells, a finding that is consistent with the stabilization of those cells. These findings are consistent with the application of formaldehyde to preserve biological samples. Future research should investigate the structural requirements of formaldehyde crosslinking (e.g., structure determination), contact time, etc., and enrichment methods to extend the number of protein crosslink identifications in samples.

## Figures and Tables

**Table 1:** Crosslinked peptides detected and validated in Streck tube samples ^1^.

ID	Crosslink	z	*m*/*z* (calc.)	*m*/*z* (obs.)	CM	RT [min]
1	AGYLEKR—RIYQFTAASPK	4	536.043	536.0420	24	40.2
2	ALEEAMEQK—ALEEAMEQKAELER	4	677.3295	677.3230	12	53.7
3	ALSTDPAAPNLKSQLAAAAR— LNEAAAGLNQAATELVQASRGTPQDLAR	5	971.715	971.715	24	102.9
4	ANLEKAK—KEEELQAALARVEEEAAQK	4 5	735.3925 588.5154	735.3925 588.5156	24	66.9 67.0
5	AQYEDIAQK—SKAEAESLYQSK	4	605.0512	605.0511	12	32
6	ATKEQLK—FQNALLVRYTK	4 5	549.0652 439.4536	549.0655 439.4538	24	43.0 43.1
7	AVFPSIVGRPR—GILTLKYPIEHGIITNWDDMEK	4 5 6	952.7618 762.4109 635.5103	952.7612 762.4112 635.5105	24	99.1 99.5 99.0
8	AVFPSIVGRPR— RGILTLKYPIEHGIITNWDDMEK	6	661.5272	661.5275	24	92.8
9	AVPAEGVR—LLLPGELAKHAVSEGTK	4	646.8669	646.8665	24	91.9
10	DAMVARAR—LGGSQEDQIKNAIDK	4	632.8276	632.8277	24	50.7
11	DAQIFIQKK—NFTEVHPDYGSHIQALLDK	4 5	822.178 657.9439	822.1781 657.9442	12	73.1 73.1
12	DAVTYTEHAKR—RSTITSREIQTAVR	5	587.1137	587.1135	24	31.7
13	DAVTYTEHAKR—STITSREIQTAVR	4 5	694.6151 555.8935	694.6149 555.8934	24	36.8 36.9
14	DLKDFIR—IGQLSEKEIQK	5	438.8484	439.2515	12	42.8
15	EEALNNAFK—ALEEAMEQKAELER	4	677.0826	677.33375 *	24	53.9
16	EKGRDLAPR—EKGRDLAPR	4	527.2936	527.2959	24	75.9
17	EKYISLR—IGQLSEKEIQK	5	439.2515	439.4538 *	12	43.1
18	ELSGPEGK—FQNALLVRYTK	4	548.7982	548.7982	24	43
19	FGERAFK—LAKTYETTLEK	4	544.2928	544.293	24	40.8
20	FKDCHLAR—CSTSPLLEACEFLRK	5 6	576.8849 480.9053	576.8852 480.9054	24	67.4 67.3
21	FKDCHLAR—HFENKFAVETLICS	5	553.6725	553.6729	24	65.6
22	FQNALLVRYTK—KVPQVSTPTLVEVSR	4 5	754.6802 603.9456	754.6804 603.9458	24	73.7 73.8
23	IAGEASRLAHYNK—NSLLTDIIAAYQR	4	730.3925	730.3901	12	71
24	IAGEASRLAHYNK—TVTAMDVVYALKR	4 5 6	730.6432 584.716 487.4312	730.6436 584.7161 487.4313	24	72.1 72.0 72.0
25	IAGEASR—TVTAMDVVYALKR	4	549.0479	549.0475	24	79.2
26	IASAEGR—TLGASGRYEGKIAR	4	549.0488	549.0477	12	79.4
27	KESYSVYVYK—TVTAMDVVYALK	4	647.5896	647.5913	12	74.9
28	KPAPKTPPK—KVPQVSTPTLVEVSR	4	657.3877	657.3879	24	61.8
29	KSHEESHKE—LLETECPQYIR	4	636.5603	636.5604	12	17.8
30	KSHEESHKE—LLETECPQYIRK	4 5 6	671.5841 537.4687 448.0585	671.5840 537.4684 448.0583	24	15.2 15.9 16.1
31	LKCASLQK—IAQDKLNDIK	4	532.8003	532.8015	24	39.7
32	LKQVLLHQQAK—NLLFNDNTECLAR	4	727.892	727.8919	24	65.2
33	MADMQNLVER—LLETECPQYIRK	4	692.5948	692.5941	12	58.8
34	MGVAAHKK—LLETECPQYIRK	4	604.3225	604.3228	24	28.7
35	NLAEKAK—KEEELQAALARVEEEAAQK	4	735.3925	735.3928	24	67
36	NLLFNDNTECLAR— CLAENAGDVAFVKDVTVLQNTDGNNNEAWAK	5	991.6732	991.6738	12	100.1
37	NLVNNLRK—LIELAKDMPSLK	5	471.0754	471.0758	24	68.5
38	RSTITSR—DAVTYTEHAKR	4	534.2804	534.2802	24	17.5
39	SHEESHKE—LLETECPQYIR	4	604.5366	604.5362	12	22.3
40	SHEESHKE—LLETECPQYIRK	5	511.8497	511.8496	24	23.2
41	SILENLRSK—YLQEIYNSNNQKIVNLK	4	791.6832	791.6837	24	73.1
42	SLAGMLTPYVAR—IAGEASRLAHYNK	4	680.6142	680.6152	12	61.4
43	VAQQEIK—FQNALLVRYTK	5	439.0505	439.2513 *	24	42.9
44	VPQSGTLK—EQIQVPSNLALK	4	548.8126	549.0652 *	24	43.1
45	VVAGVANALAHK— VLGAFSDGLAHLDNLKGTFATLSELHCDK	6	715.8784	715.8791	12	101.9

^1^ observed (obs.); calculated (calc.); charge state (z); mass (m); crosslink mass shift (CM); average retention time (RT); * isotope error was detected by FAXL finder. The number ID of the crosslink is the same as for Table 2.

**Table 2:** Proteins associated to crosslinked peptides in Streck tube samples.

ID	Protein 1				Protein 2			
	Name	MW [kD]	Gene Name	Uniprot ID	Name	MW [kD]	Gene Name	Uniprot ID
1	Src kinase-associated phosphoprotein 2	41.2	SKAP2	O75563	Src kinase-associated phosphoprotein 2	42.2	SKAP2	O75563
2	Myosin-9	226.5	MYH9	P35579	Myosin-9	226.5	MYH9	P35579
3	Talin-1	269.8	TLN1	Q9Y490	Talin-1	269.8	TLN1	Q9Y490
4	Myosin-9	226.5	MYH9	P35579	Myosin-9	226.5	MYH9	P35579
5	Keratin	66	KRT1	P04264	Keratin	66	KRT1	P04264
6	Albumin	69.4	ALB	P02768	Albumin	69.4	ALB	P02768
7	POTE ankyrin dom. family member F/Actin	121.4 41.9	POTEF ACTG2	A5A3E0 P63267	Actin	41.9	ACTG2	P63267
8	POTE ankyrin dom. family member F/Actin	121.4 41.9	POTEF ACTG2	A5A3E0 P63267	Actin	41.9	ACTG2	P63267
9	Mannosyl-oligosaccharide glucosidase	91.9	MOGS	Q13724	Histone H2B	13.9	H2BC12	O60814
10	Transaldolase	37.5	TALDO1	P37837	Transaldolase	37.5	TALDO1	P37837
11	Catalase	59.8	CAT	P04040	Catalase	59.8	CAT	P04040
12	Histone H4	11.4	H4C1-16	P62805	Histone H2B	13.9	H2BC12	O60814
13	Histone H4	11.4	H4C1-16	P62805	Histone H2B	13.9	H2BC12	O60814
14	Potassium voltage-gated channel subfamily H member 8	123.8	KCNH8	Q96L42	Nexilin	80.7	NEXN	Q0ZGT2
15	Arf-GAP with SH3 dom., ANK repeat and PH dom.-containing protein 2	111.7	ASAP2	O43150	Myosin-9	226.5	MYH9	P35579
16	Multiple epidermal growth-factor-like dom. protein 11	110.8	MEGF11	A6BM72	Multiple epidermal growth factor-like dom. protein 11	110.8	MEGF11	A6BM72
17	GRIP and coiled-coil dom.-containing protein 1	87.8	GCC1	Q96CN9	Nexilin	80.7	NEXN	Q0ZGT2
18	Docking protein 2	45.4	DOK2	O60496	Albumin	69.4	ALB	P02768
19	Albumin	69.4	ALB	P02768	Albumin	69.4	ALB	P02768
20	Lactotransferrin	78.2	LTF	P02788	Lactotransferrin	78.2	LTF	P02788
21	Lactotransferrin	78.2	LTF	P02788	Integral membrane protein 2B	30.3	ITM2B	Q9Y287
22	Albumin	69.4	ALB	P02768	Albumin	69.4	ALB	P02768
23	Histone H2B	13.9	H2BC12	O60814	AFG3-like protein 2	88.6	AFG3L2	Q9Y4W6
24	Histone H2B	13.9	H2BC12	O60814	Histone H4	11.4	H4C1-16	P62805
25	Histone H2B	13.9	H2BC12	O60814	Histone H4	11.4	H4C1-16	P62805
26	Olfactory receptor 10J4	34.9	OR10J4	P0C629	Indian hedgehog protein	45.25	IHH	Q14623
27	Histone H2B	13.9	H2BC12	O60814	Histone H4	11.4	H4C1-16	P62805
28	Myosin light chain kinase	210.7	MYLK	Q15746	Albumin	69.4	ALB	P02768
29	Protein S100-A8	10.8	S100A8	P05109	Protein S100-A8	10.8	S100A8	P05109
30	Protein S100-A8	10.8	S100A8	P05109	Protein S100-A8	10.8	S100A8	P05109
31	Albumin	69.4	ALB	P02768	DNA repair protein RAD50	153.9	RAD50	Q92878
32	Lactotransferrin	78.2	LTF	P02788	Lactotransferrin	78.2	LTF	P02788
33	Adenylyl cyclase-associated protein 1	51.9	CAP1	Q01518	Protein S100-A8	10.8	S100A8	P05109
34	Protein S100-A8	10.8	S100A8	P05109	Protein S100-A8	10.8	S100A8	P05109
35	Protein mono-ADP-ribosyltransferase PARP9	96.3	PARP9	Q8IXQ6	Myosin-9	226.5	MYH9	P35579
36	Lactotransferrin	78.2	LTF	P02788	Lactotransferrin	78.2	LTF	P02788
37	Myeloid cell nuclear differentiation antigen	45.8	MNDA	P41218	Myeloid cell nuclear differentiation antigen	45.8	MNDA	P41218
38	Histone H2B	13.9	H2BC12	O60814	Histone H4	11.4	H4C1-16	P62805
39	Protein S100-A8	10.8	S100A8	P05109	Protein S100-A8	10.8	S100A8	P05109
40	Protein S100-A8	10.8	S100A8	P05109	Protein S100-A8	10.8	S100A8	P05109
41	Fibrinogen beta chain	55.9	FGB	P02675	Fibrinogen gamma chain	51.5	FGG	P02679
42	DnaJ homolog subfamily C member 13	254.4	DNAJC13	O75165	Histone H2B	13.9	H2BC12	O60814
43	Inositol 1,4,5-trisphosphate receptor type 1\	313.9	ITPR1	Q14643	Albumin	69.4	ALB	P02768
44	Low-density lipoprotein receptor-related protein 11	53.3	LRP11	Q86VZ4	Ras-related protein Rab-33A	26.6	RAB33A	Q14088
45	Hemoglobin subunit delta	16.1	HBD	P02042	Hemoglobin subunit beta	16	HBB	P68871

Molecular weight (MW); number ID of crosslink is the same as for Table 1; domain (dom.).

## Data Availability

Raw data will be made available upon request.

## Supplementary Materials

The following supporting information can be downloaded at: https://www.mdpi.com/article/10.3390/molecules28237880/s1. S1: S1_All_FAXL_over_1.5.xlsx, Excel spread sheet summarizing the output from Formaldehyde_XL_Analyzer software from the Kalisman lab (http://biolchem.huji.ac.il/nirka/index.html; last accessed on 29 November 2023) using three different protein FASTA databases (detected, HPA, membrane). See: http://biolchem.huji.ac.il/nirka/Software/Formaldehyde_2020/Read_Me.pdf; last accessed on 29 November 2023; S2: S2_ XICs_in_Heatmap_order.pdf; Extracted ion chromatograms for the validation of all potential crosslinks in S samples. XICs for F, S, E, and A samples are shown for donors 2, 3, and 4. Each colored chromatogram represents one transition to a b or y ion.
