# Supplementary material for: Mass Spectrometric Detection of Formaldehyde-Crosslinked PBMC Proteins in Cell-Free DNA Blood Collection Tubes
Source: Molecules. 2023 Nov 30;28(23):7880. doi: 10.3390/molecules28237880 (PMC10708122; doi:10.3390/molecules28237880)
Supplement: Supplementary file 1 [file molecules-28-07880-s001.zip › S2_ XICs_in_Heatmap_order.pdf]

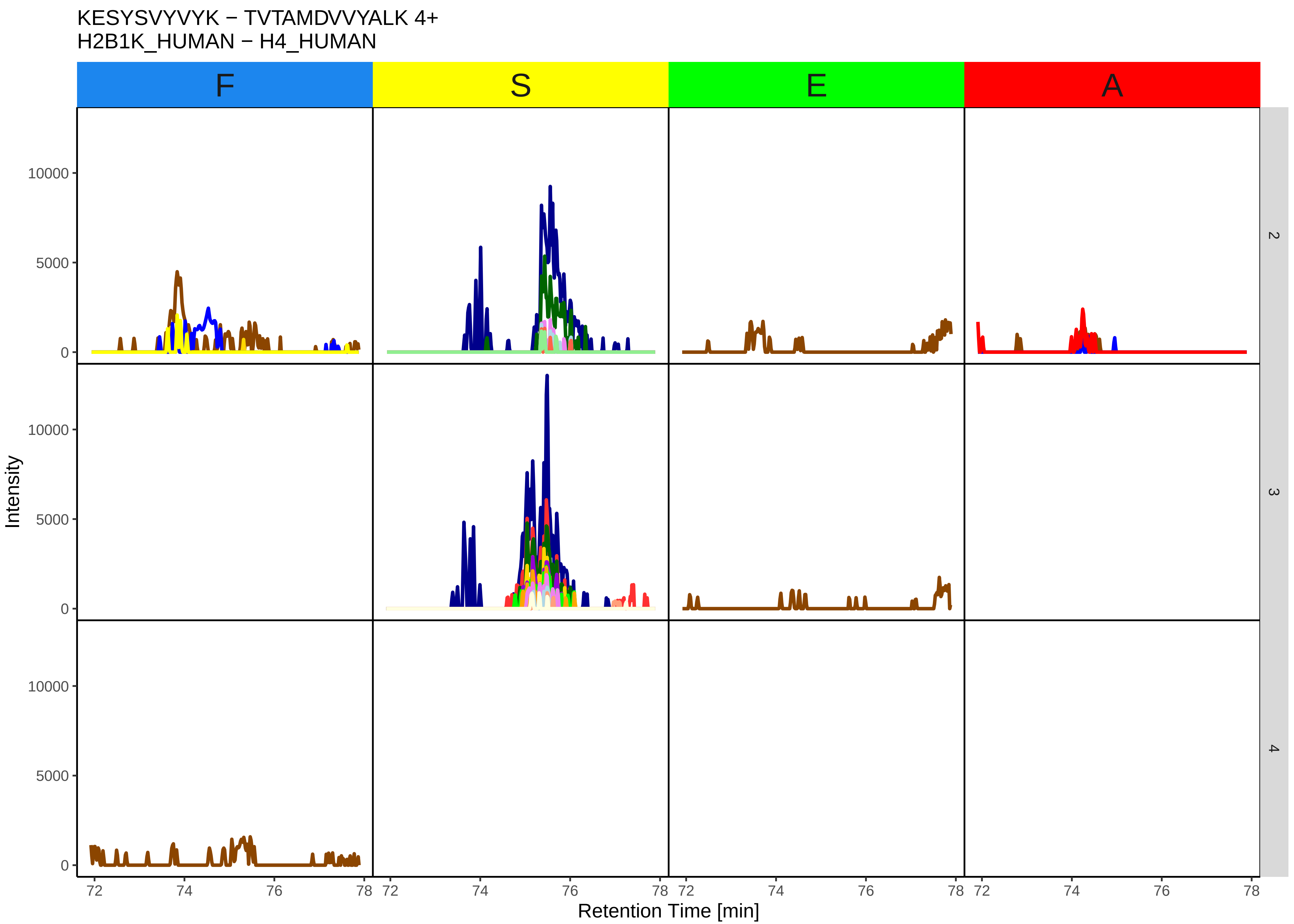
KESYSVYVYK − TVTAMDVVYALK 4+
H2B1K_HUMAN − H4_HUMAN
F
S
E
A
2
3
4
Intensity
10000
5000
0
72
74
76
78
Retention Time [min]

VAQQEIK − FQNALLVRYTK 5+
ITPR1_HUMAN − ALBU_HUMAN

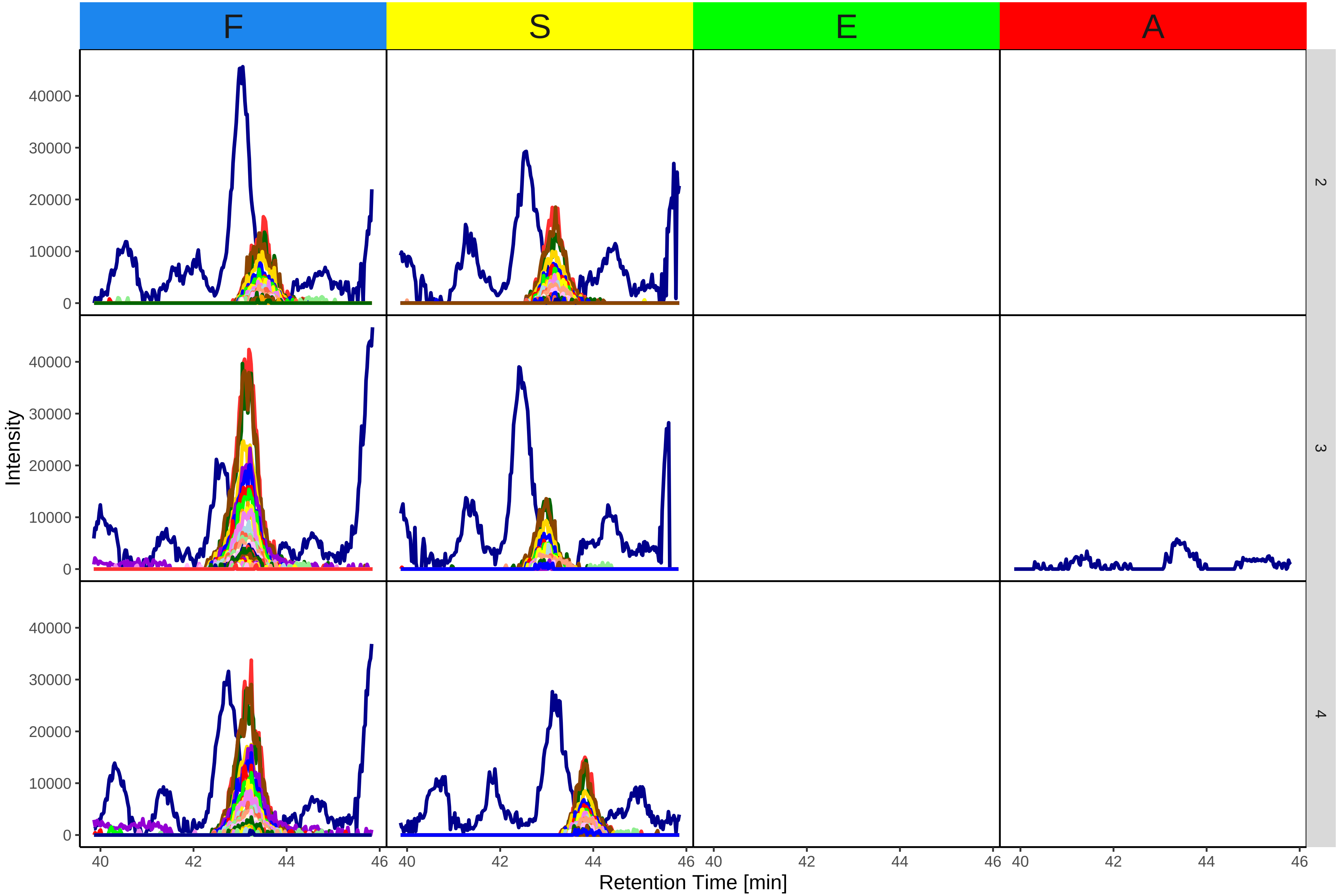

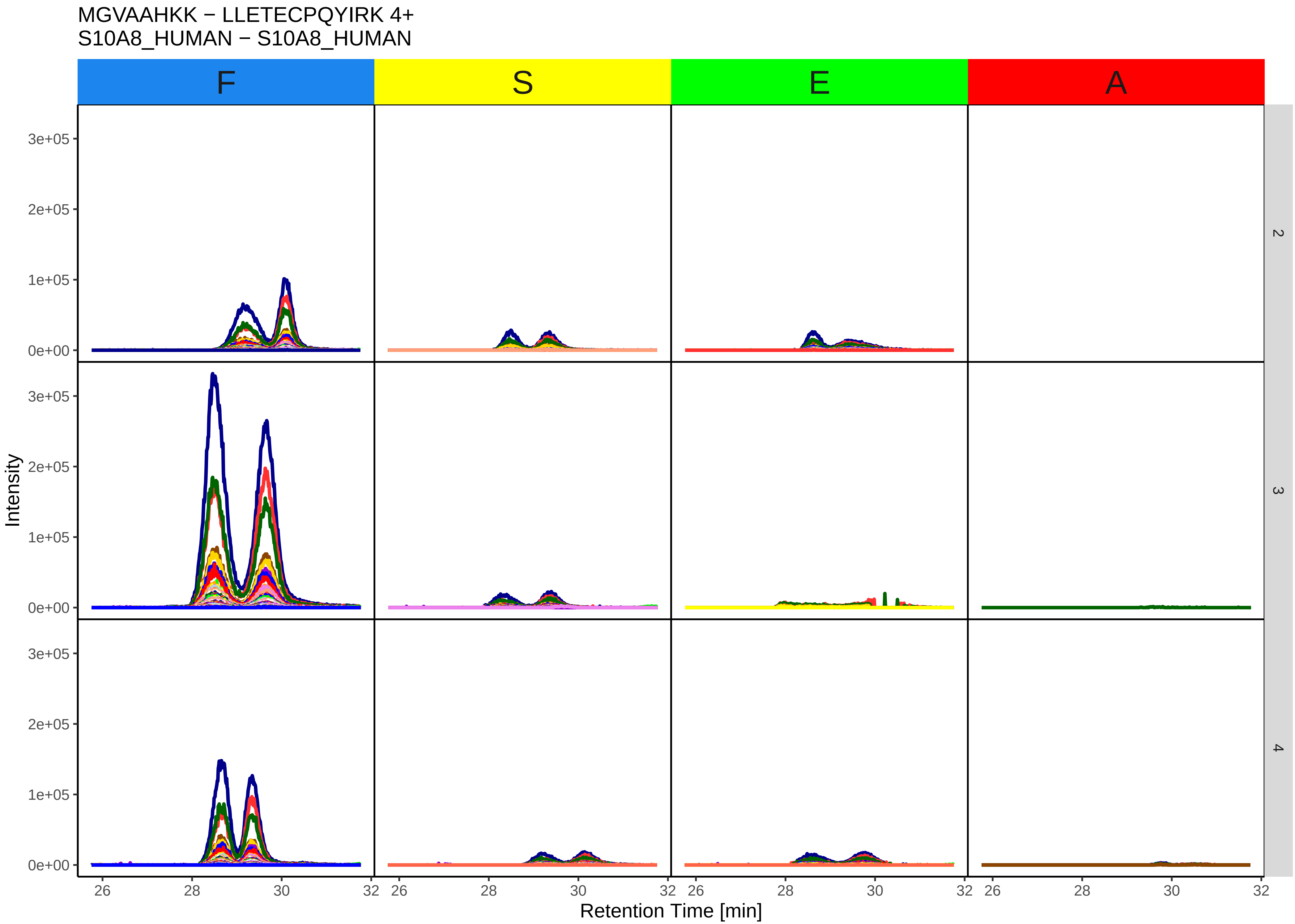
MGVAAHKK − LLETECPQYIRK 4+
S10A8_HUMAN − S10A8_HUMAN
F
S
E
A
2
3
4
Intensity
3e+05
2e+05
1e+05
0e+00
26
28
30
32
Retention Time [min]

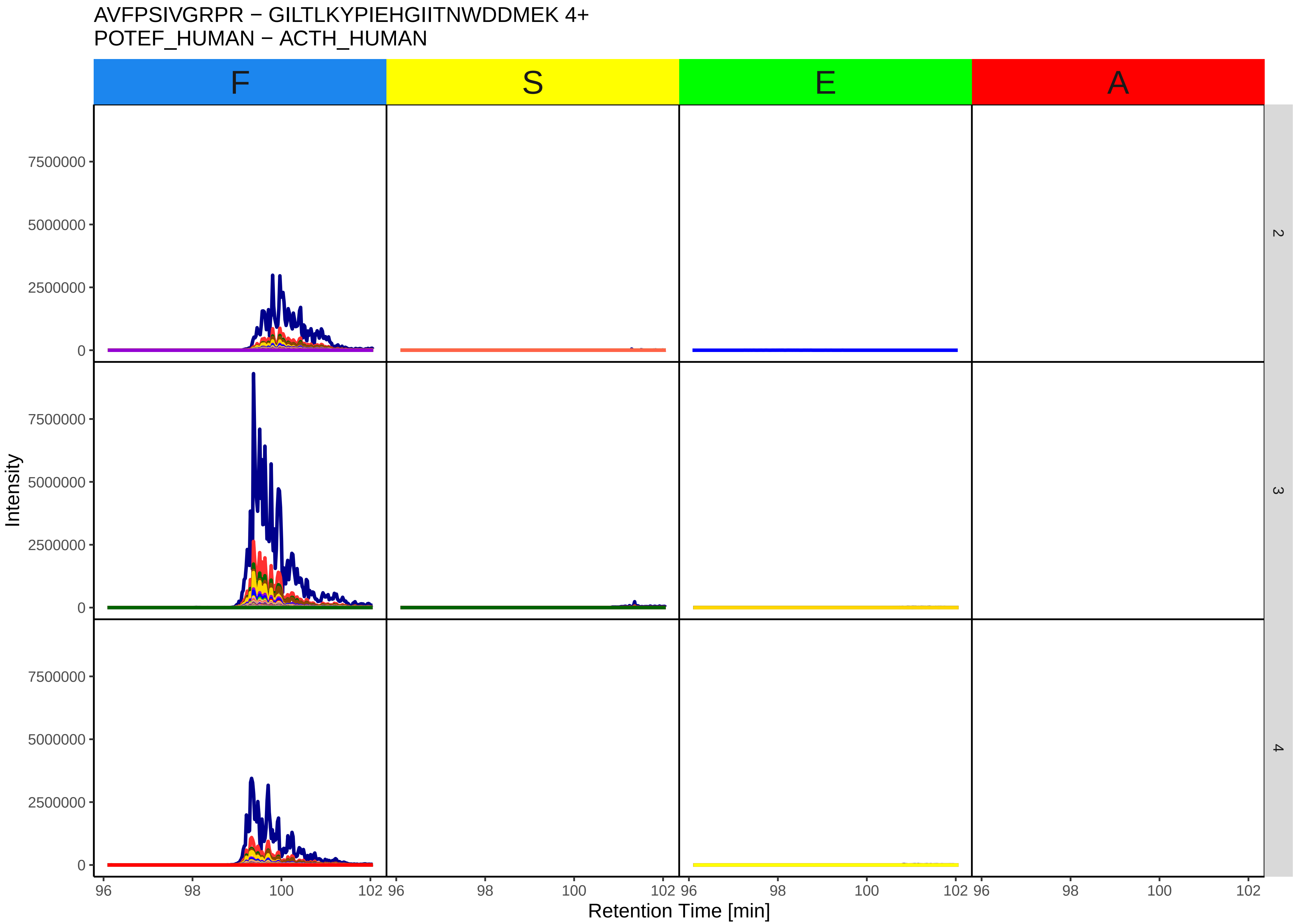

AVFPSIVGRPR − GILTLKYPIEHGIITNWDDMEK 4+
POTEF_HUMAN − ACTH_HUMAN
F
S
E
A
2
3
4
Intensity
7500000
5000000
2500000
0
96
98
100
102
Retention Time [min]

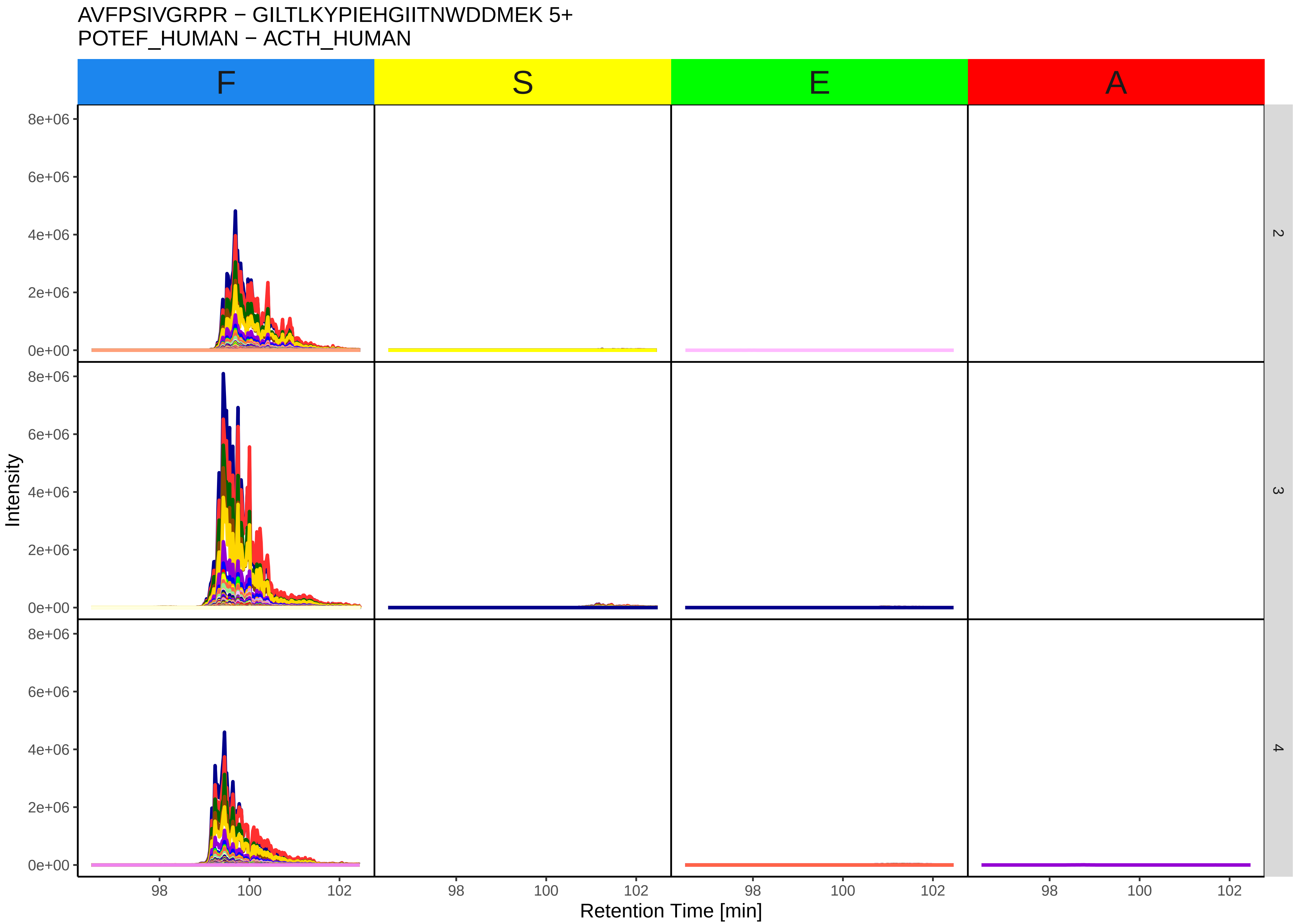

AVFPSIVGRPR − GILTLKYPIEHGIITNWDDMEK 5+
POTEF_HUMAN − ACTH_HUMAN
F
S
E
A
Intensity
8e+06
6e+06
4e+06
2e+06
0e+00
2
3
4
98
100
102
Retention Time [min]

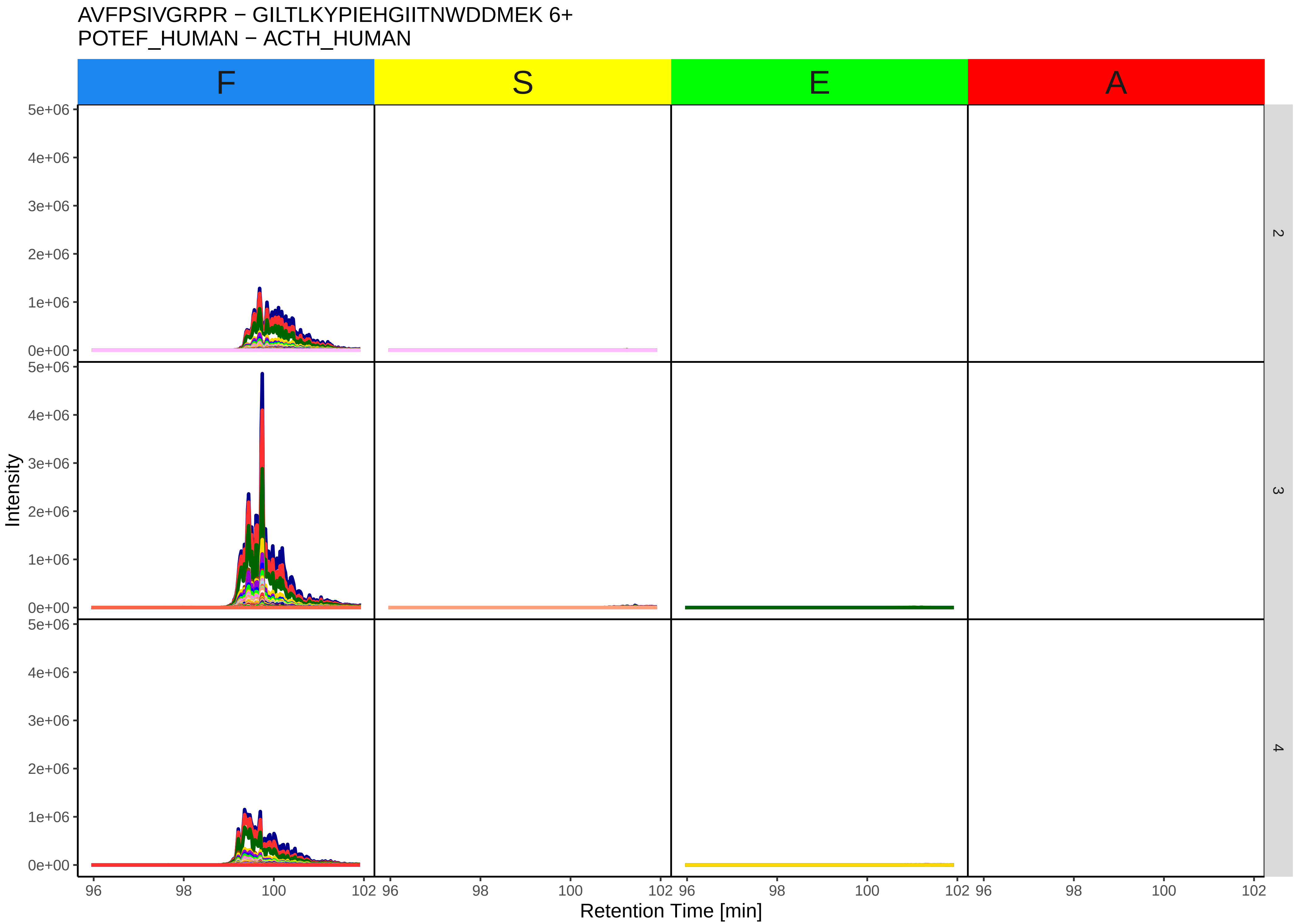
AVFPSIVGRPR − GILTLKYPIEHGIITNWDDMEK 6+
POTEF_HUMAN − ACTH_HUMAN
F
S
E
A
Intensity
5e+06
4e+06
3e+06
2e+06
1e+06
0e+00
2
3
4
96
98
100
102
Retention Time [min]

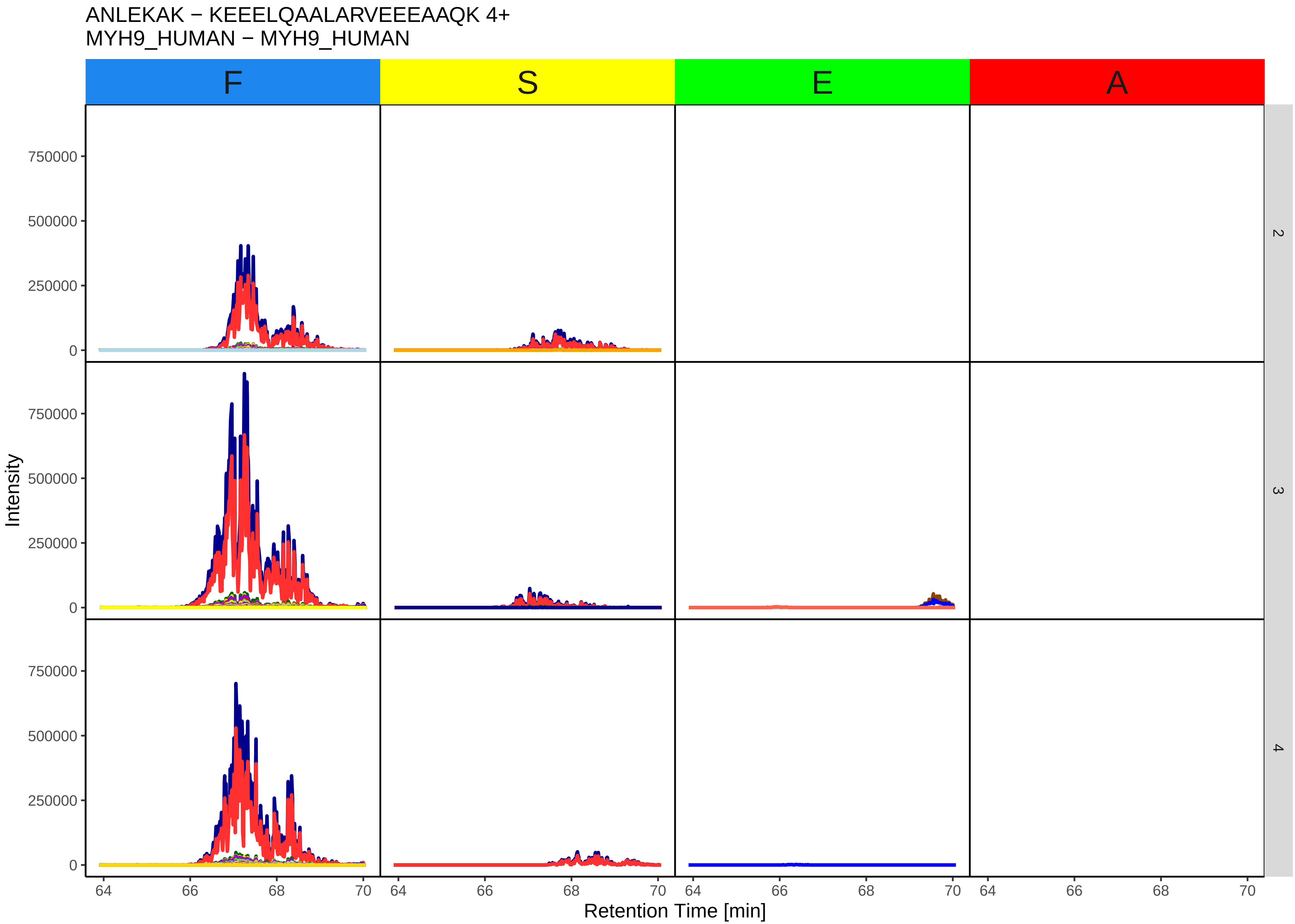

ANLEKAK − KEEELQAALARVEEEAAQK 4+
MYH9_HUMAN − MYH9_HUMAN
F
S
E
A
2
3
4
Intensity
750000
500000
250000
0
64
66
68
70
Retention Time [min]

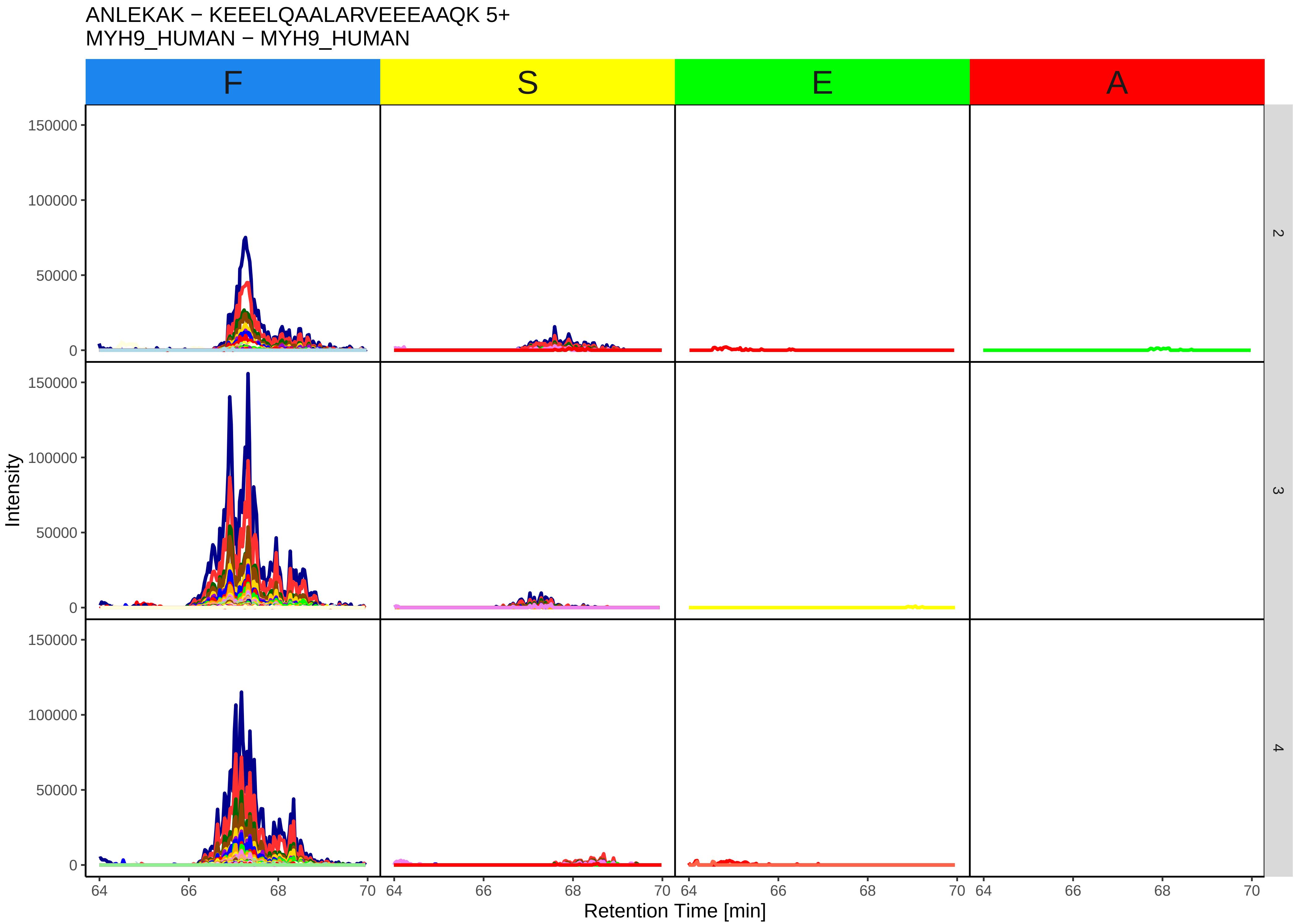
ANLEKAK − KEEELQAALARVEEEAAQK 5+
MYH9_HUMAN − MYH9_HUMAN
F
S
E
A
Intensity
0
50000
100000
150000
2
3
4
64
66
68
70
Retention Time [min]

KSHEESHKE − LLETECPQYIR 4+
S10A8_HUMAN − S10A8_HUMAN

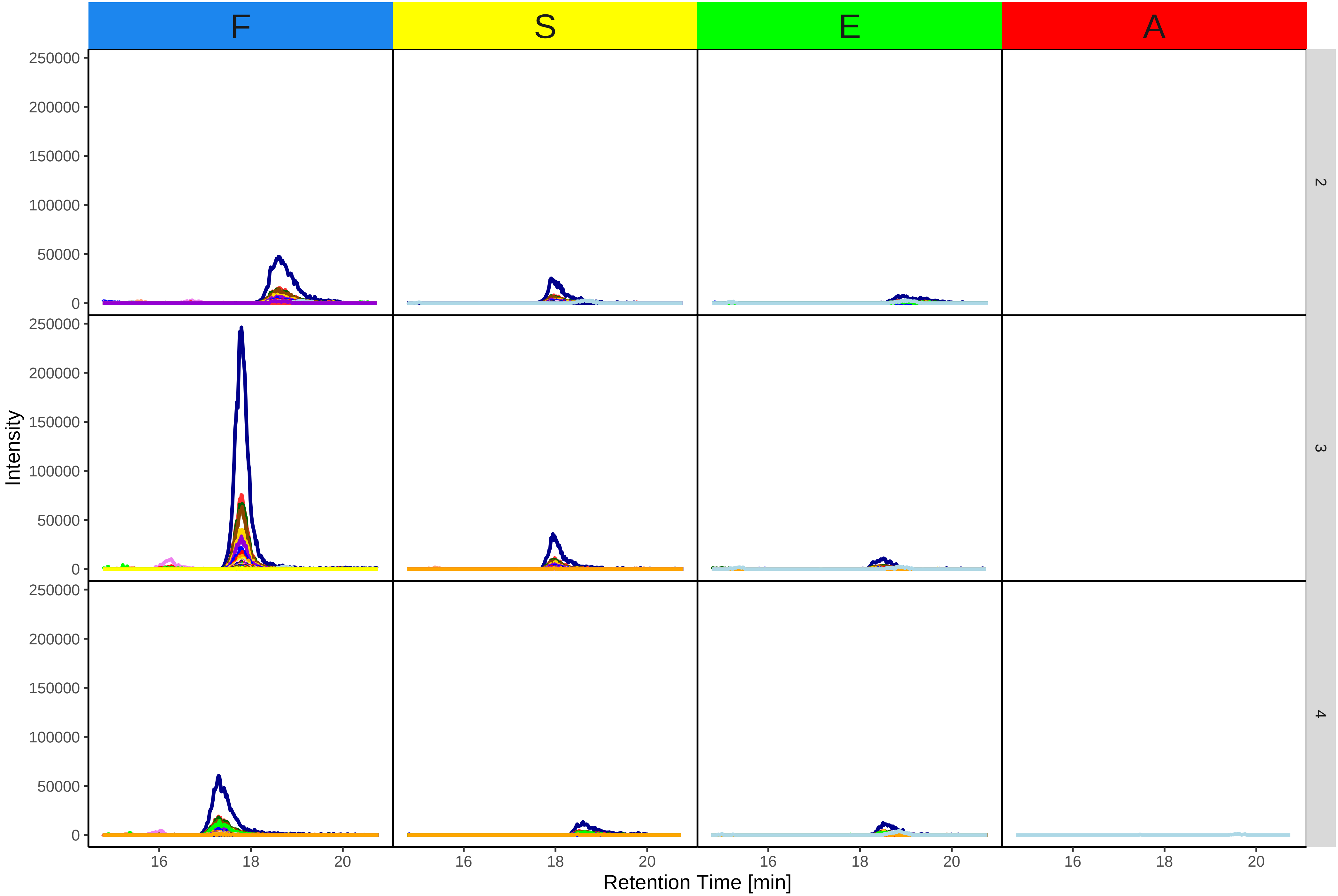

LKQVLLHQQAK − NLLFNDNTECLAR 4+
TRFL_HUMAN − TRFL_HUMAN

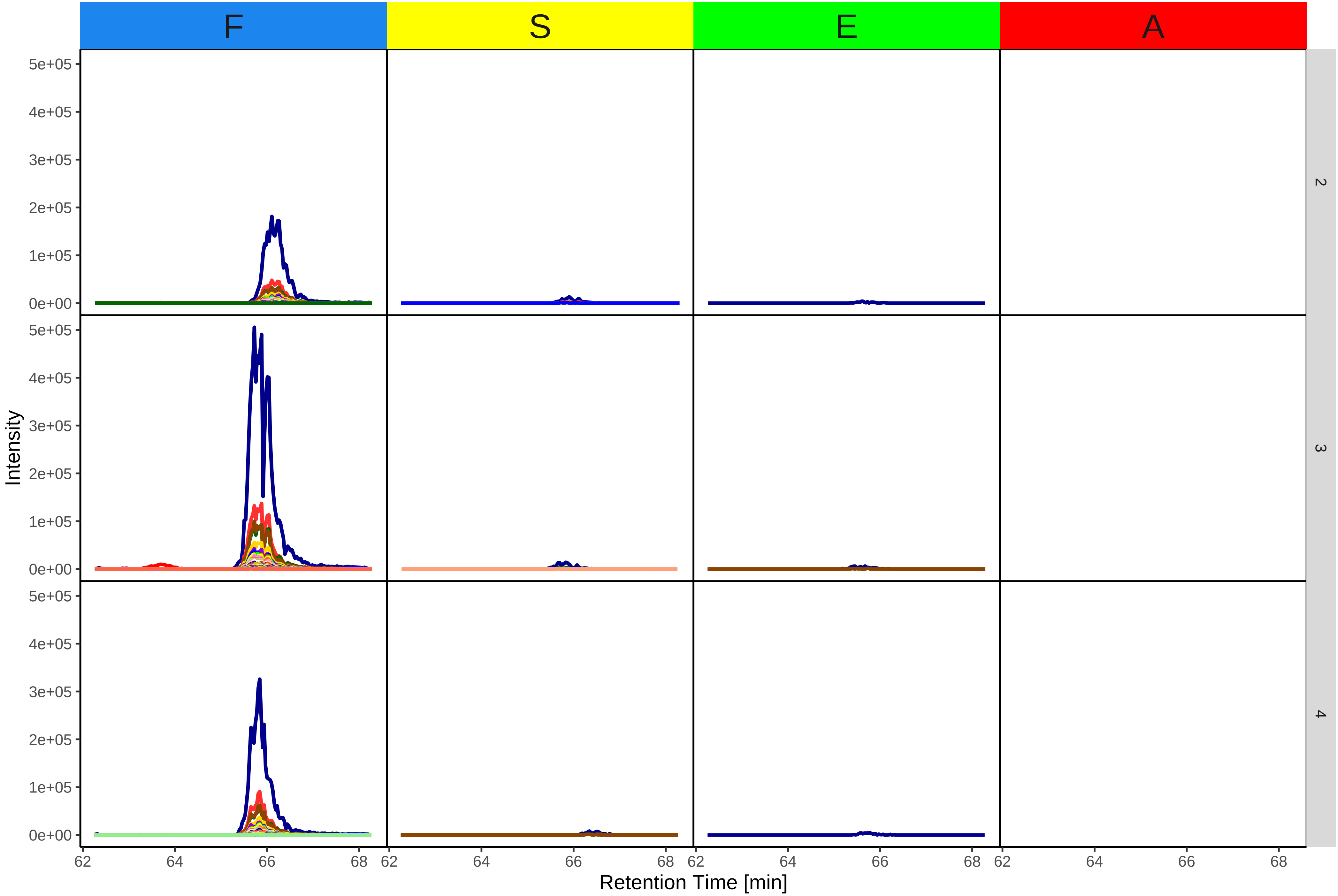

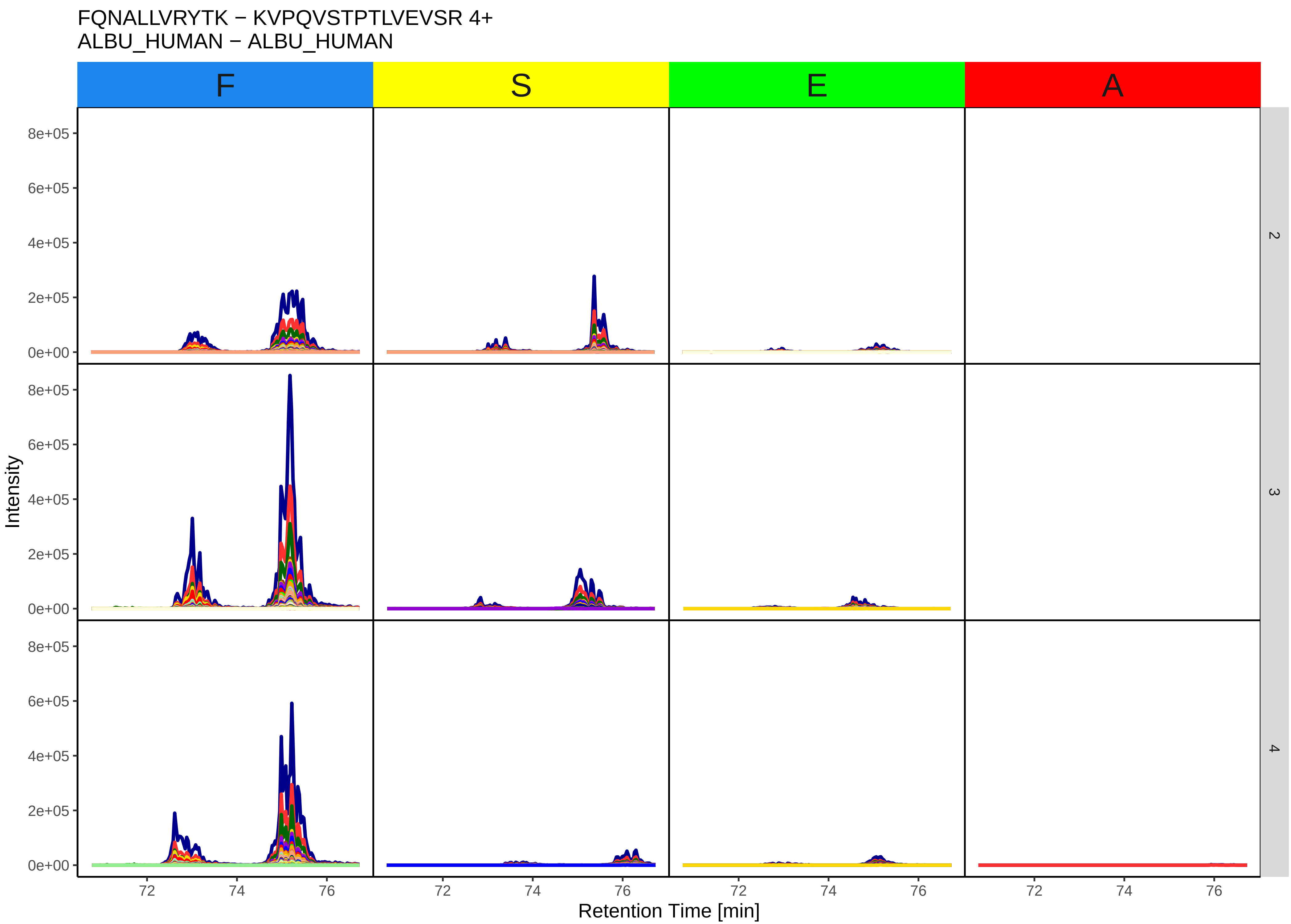
FQNALLVRYTK − KVPQVSTPTLVEVSR 4+
ALBU_HUMAN − ALBU_HUMAN
F
S
E
A
2
3
4
Intensity
8e+05
6e+05
4e+05
2e+05
0e+00
72
74
76
Retention Time [min]

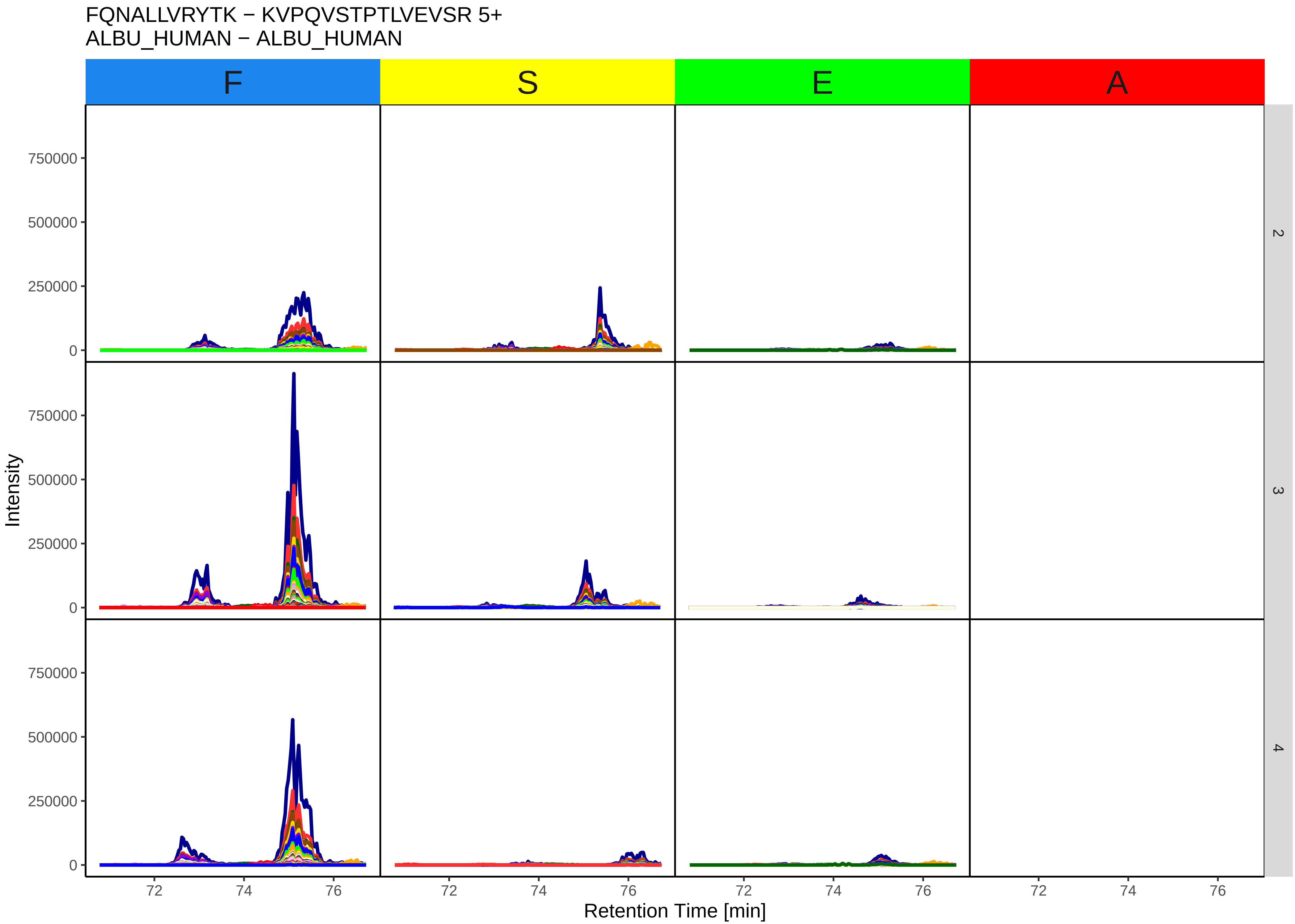
FQNALLVRYTK − KVPQVSTPTLVEVSR 5+
ALBU_HUMAN − ALBU_HUMAN
F
S
E
A
2
3
4
750000
500000
250000
0
Intensity
72
74
76
Retention Time [min]

ALSTDPAAPNLKSQLAAAAR − LNEAAAGLNQAATELVQASRGTPQDLAR 5+
TLN1_HUMAN − TLN1_HUMAN

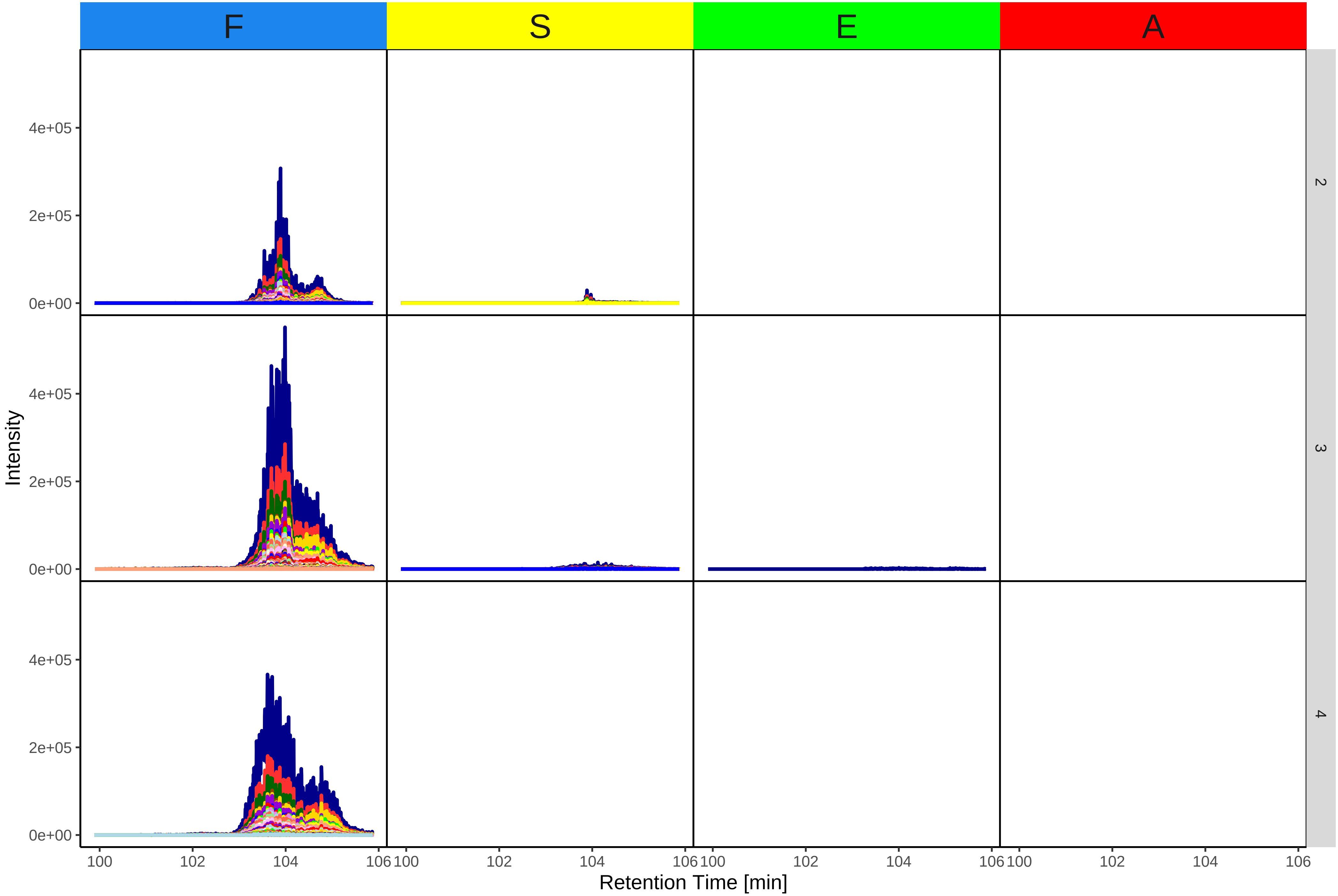

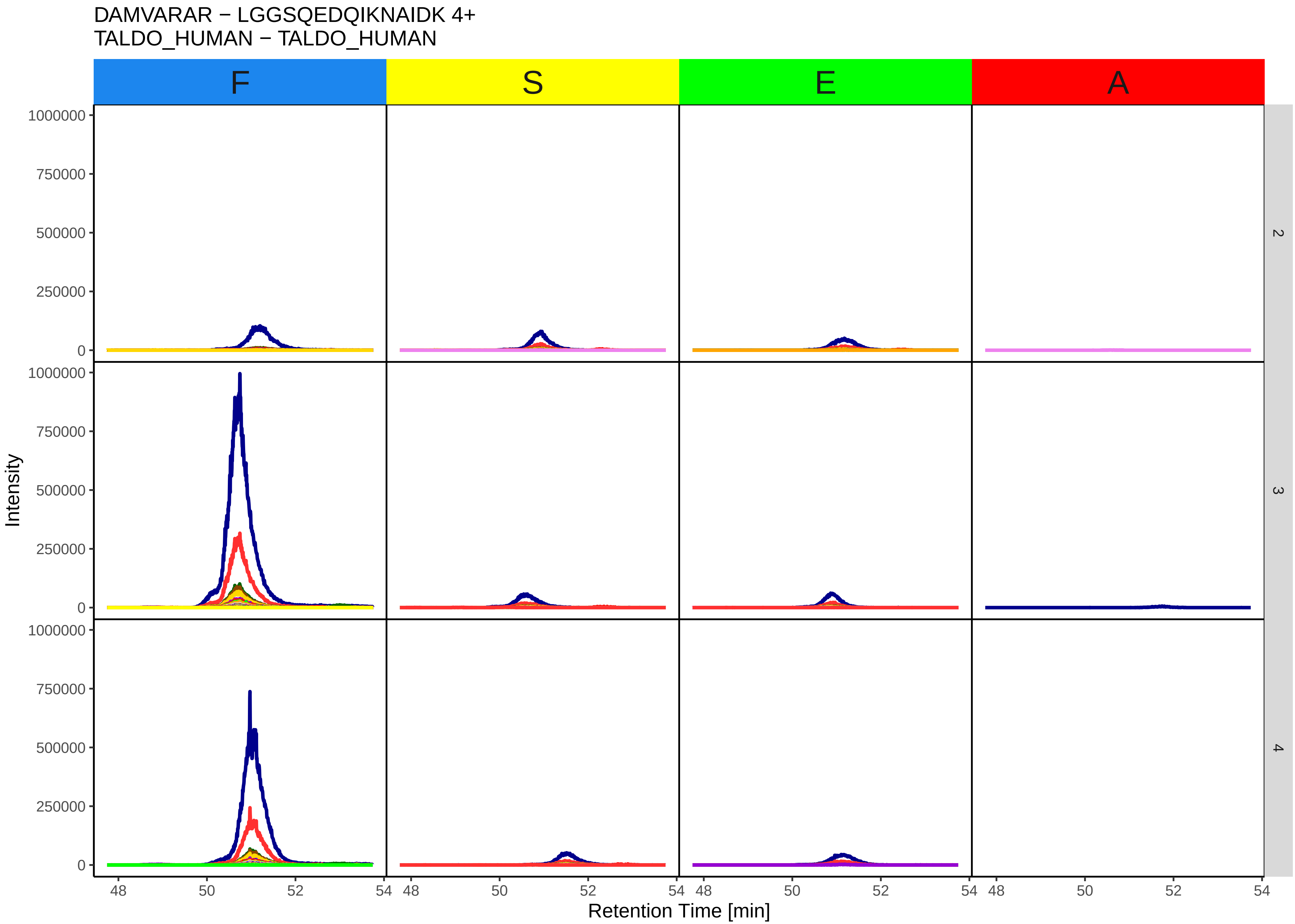
DAMVARAR − LGGSQEDQIKNAIDK 4+
TALDO_HUMAN − TALDO_HUMAN
F
S
E
A
2
3
4
Intensity
1000000
750000
500000
250000
0
48
50
52
54
Retention Time [min]

SHEESHKE − LLETECPQYIRK 5+
S10A8_HUMAN − S10A8_HUMAN

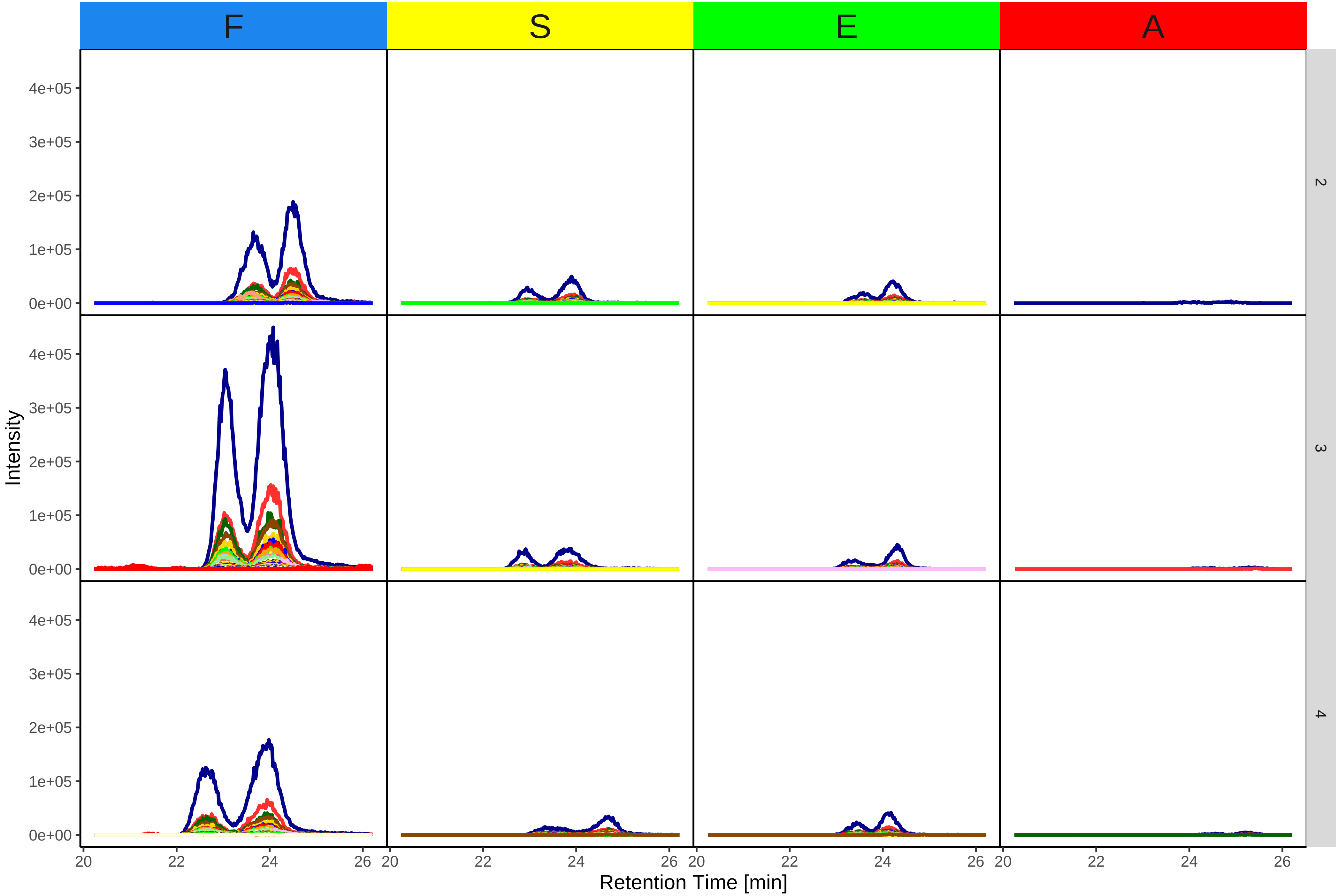

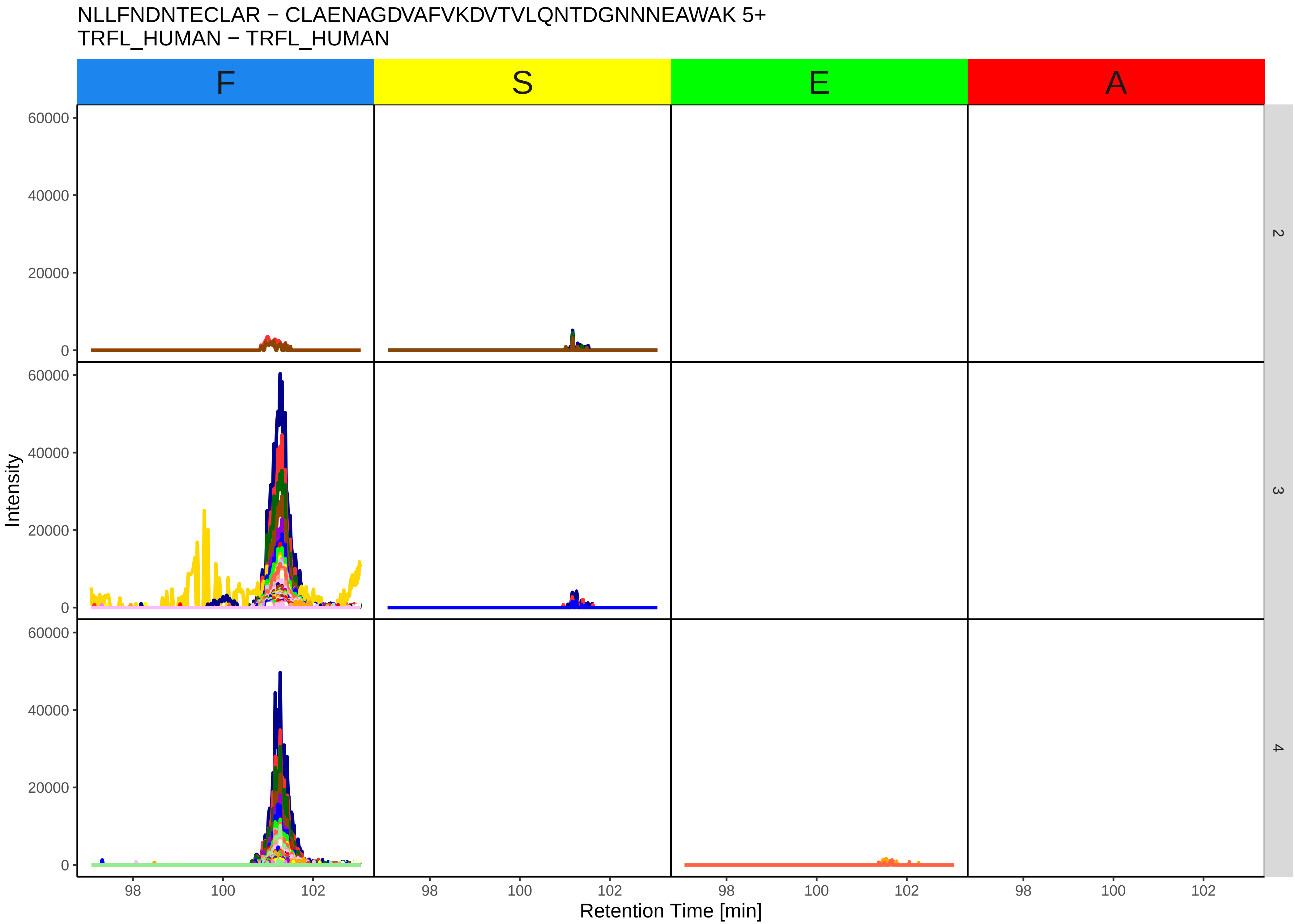

NLLFNDNTECLAR − CLAENAGDVAFVKDVTVLQNTDGNNNEAWAK 5+
TRFL_HUMAN − TRFL_HUMAN
F
S
E
A
2
3
4
Intensity
60000
40000
20000
0
98
100
102
Retention Time [min]

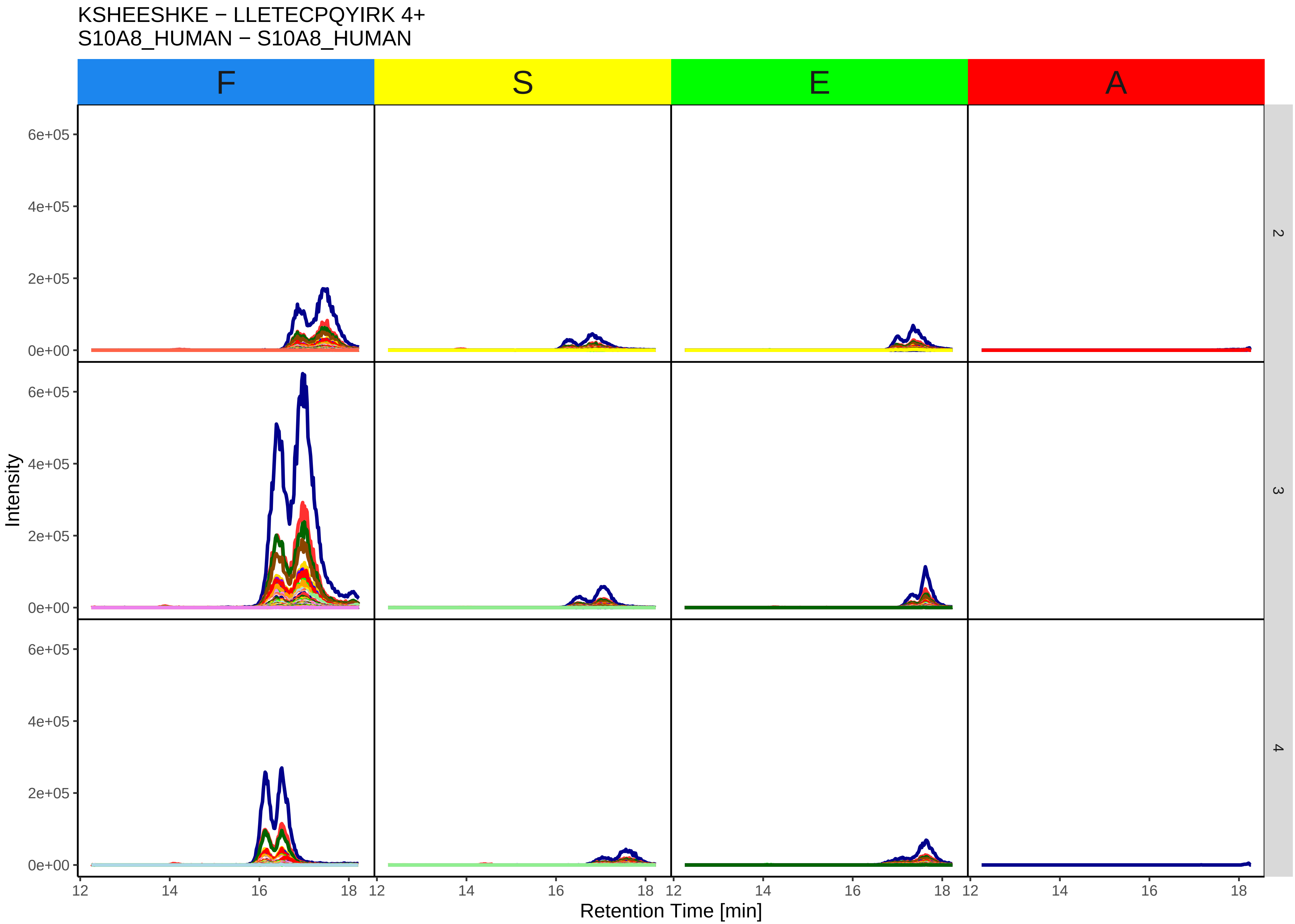

KSHEESHKE − LLETECPQYIRK 4+
S10A8_HUMAN − S10A8_HUMAN
F
S
E
A
2
3
4
6e+05
4e+05
2e+05
0e+00
Intensity
12
14
16
18
Retention Time [min]

KSHEESHKE − LLETECPQYIRK 5+
S10A8_HUMAN − S10A8_HUMAN

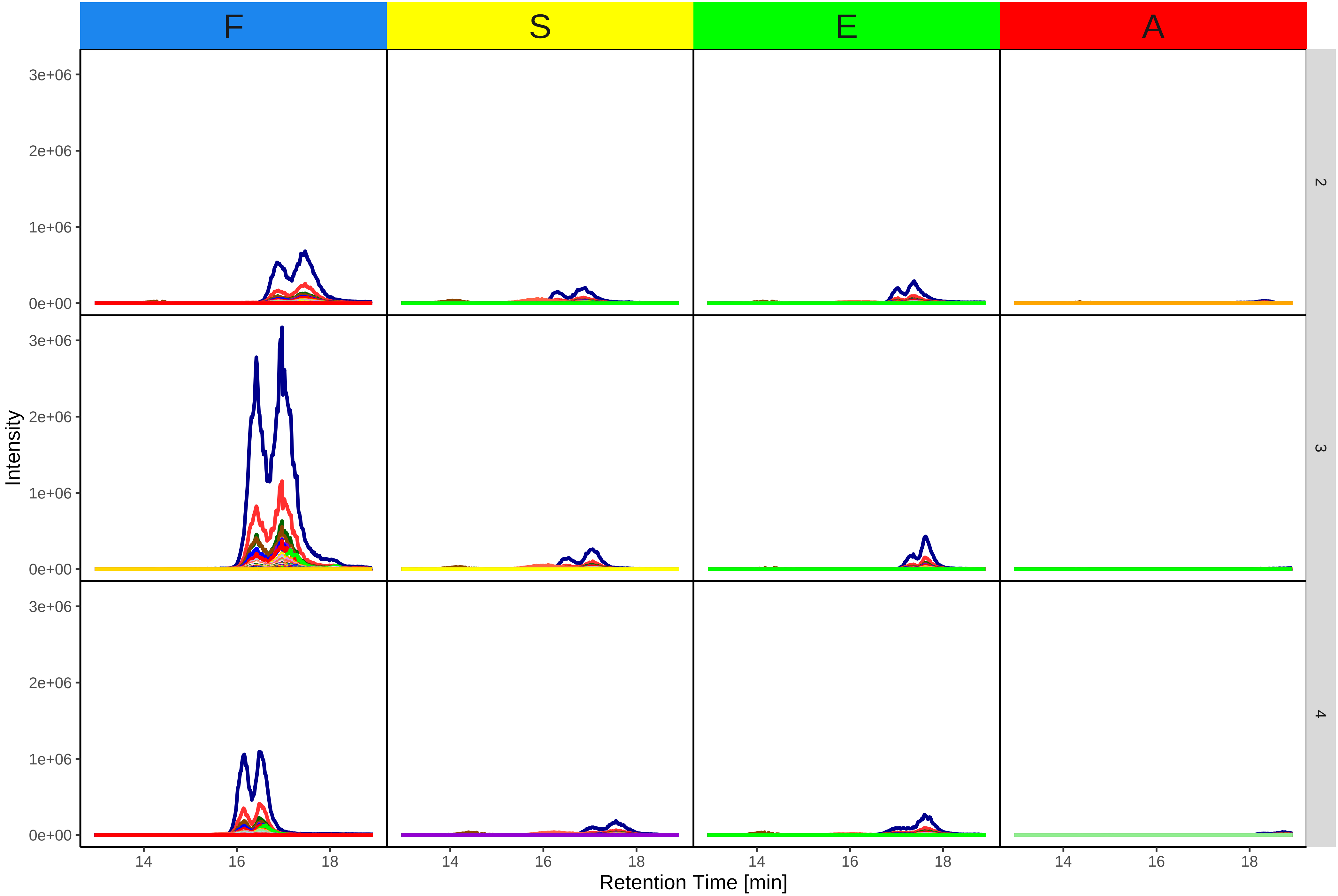

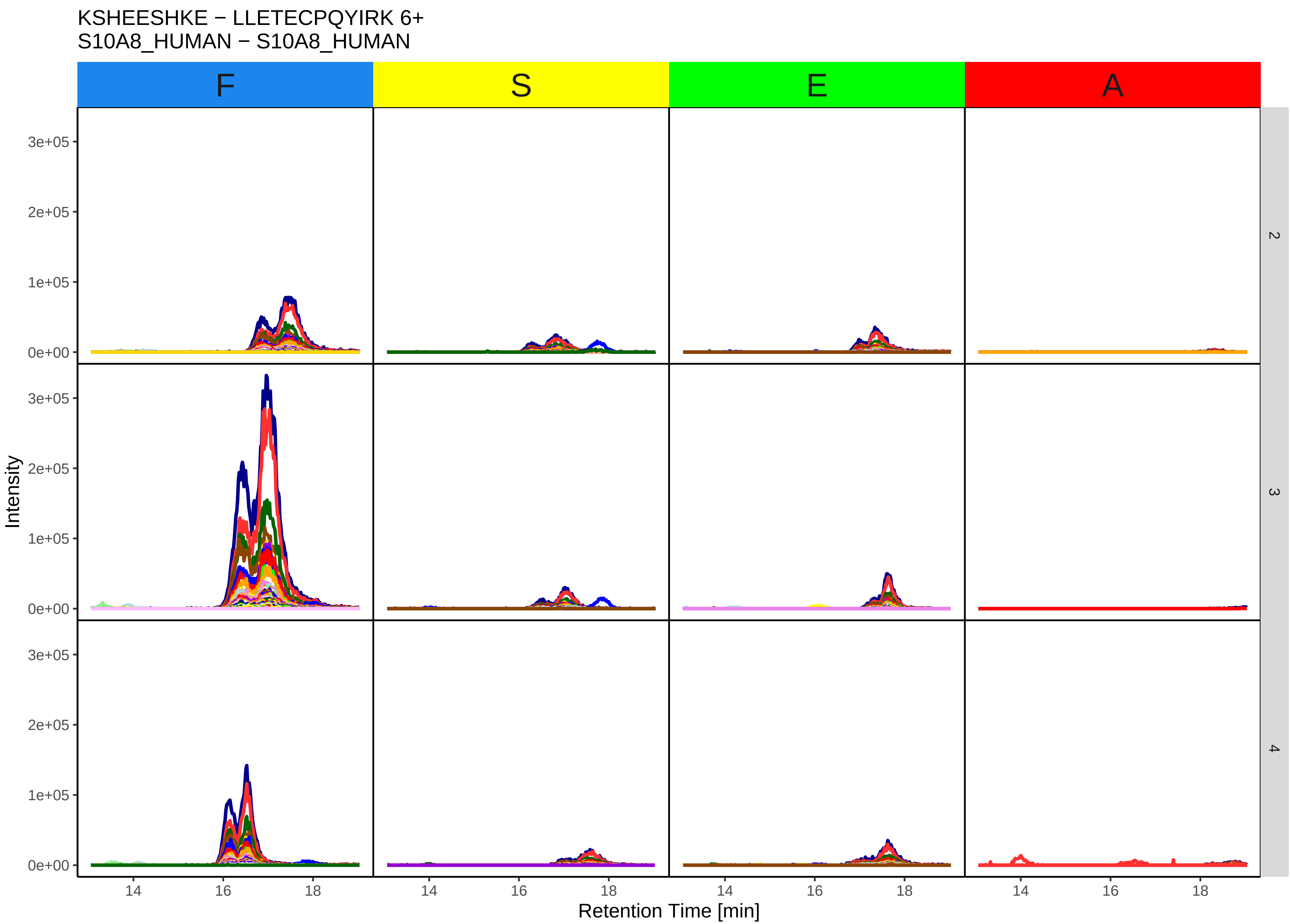

KSHEESHKE − LLETECPQYIRK 6+
S10A8_HUMAN − S10A8_HUMAN
F
S
E
A
2
3
4
3e+05
2e+05
1e+05
0e+00
Intensity
14
16
18
Retention Time [min]

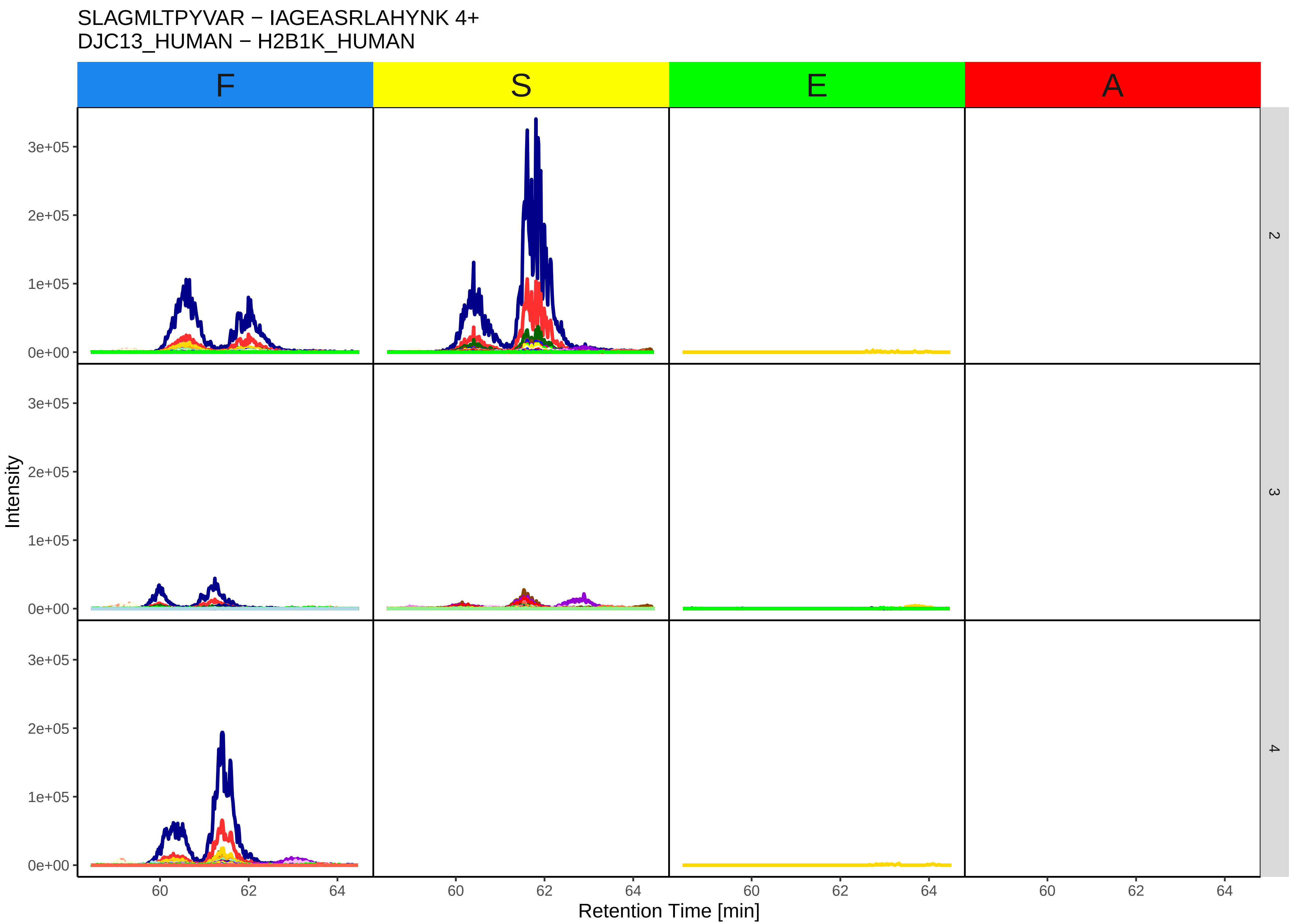
SLAGMLTPYVAR − IAGEASRLAHYNK 4+
DJC13_HUMAN − H2B1K_HUMAN
F
S
E
A
2
3
4
Intensity
3e+05
2e+05
1e+05
0e+00
60
62
64
Retention Time [min]

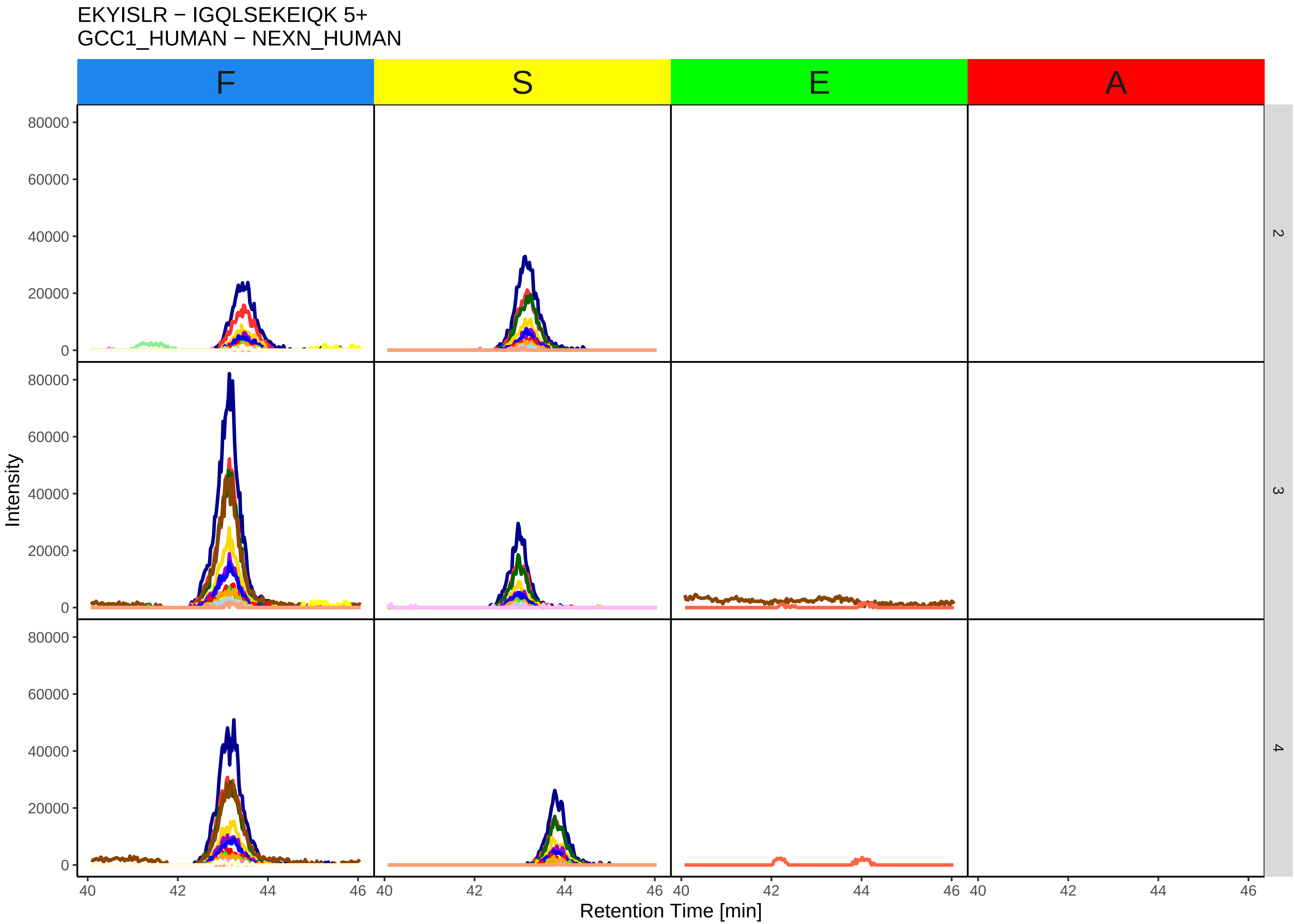

EKYISLR − IGQLSEKEIQK 5+
GCC1_HUMAN − NEXN_HUMAN
F
S
E
A
2
3
4
Intensity
0
20000
40000
60000
80000
40
42
44
46
Retention Time [min]

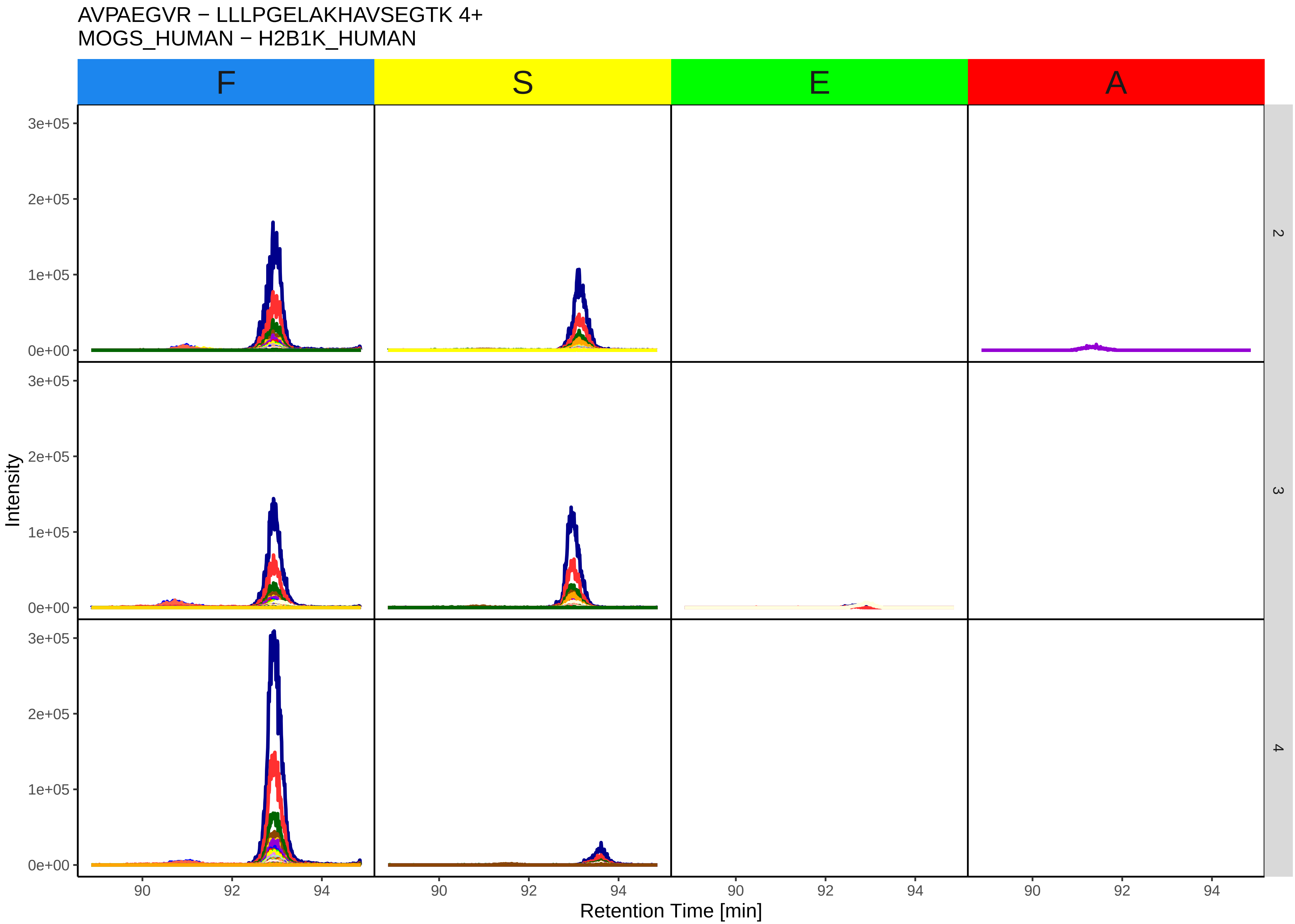

AVPAEGVR − LLLPGELAKHAVSEGTK 4+
MOGS_HUMAN − H2B1K_HUMAN
F
S
E
A
2
3
4
Intensity
3e+05
2e+05
1e+05
0e+00
90
92
94
Retention Time [min]

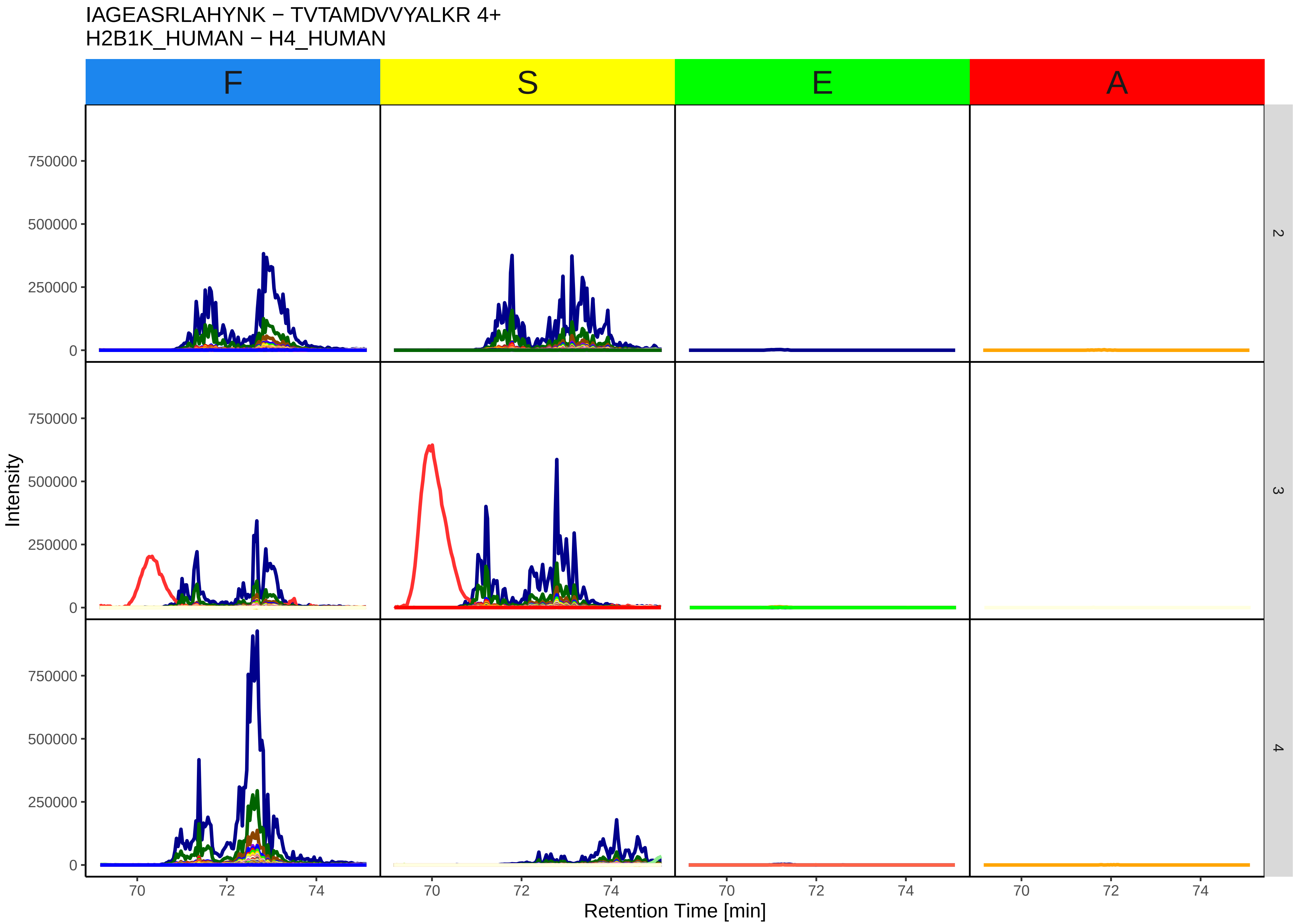
IAGEASRLAHYNK − TVTAMDVVYALKR 4+
H2B1K_HUMAN − H4_HUMAN
F
S
E
A
2
3
4
Intensity
750000
500000
250000
0
70
72
74
Retention Time [min]

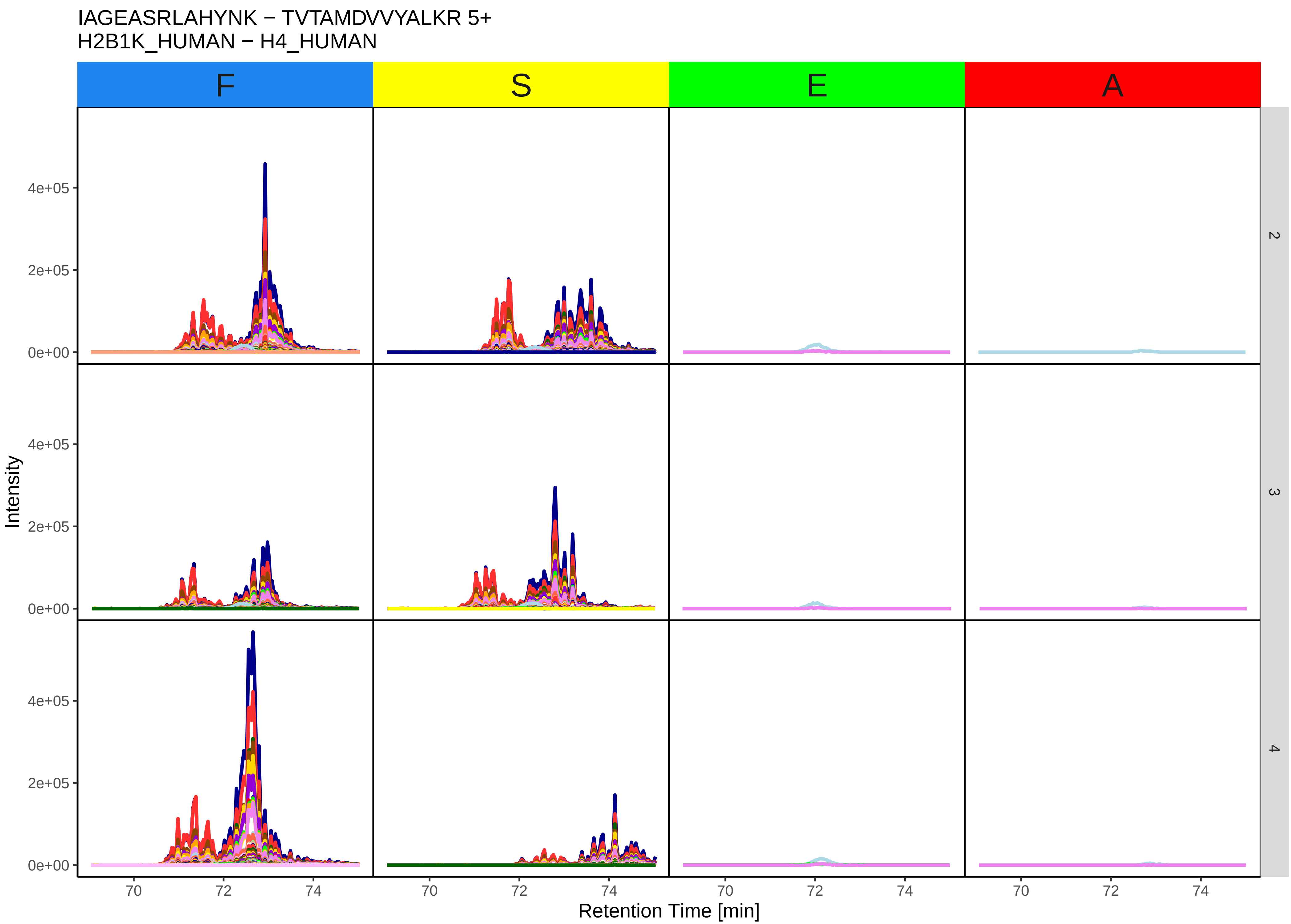
IAGEASRLAHYNK − TVTAMDVVYALKR 5+
H2B1K_HUMAN − H4_HUMAN
F
S
E
A
2
3
4
Intensity
4e+05
2e+05
0e+00
70
72
74
Retention Time [min]

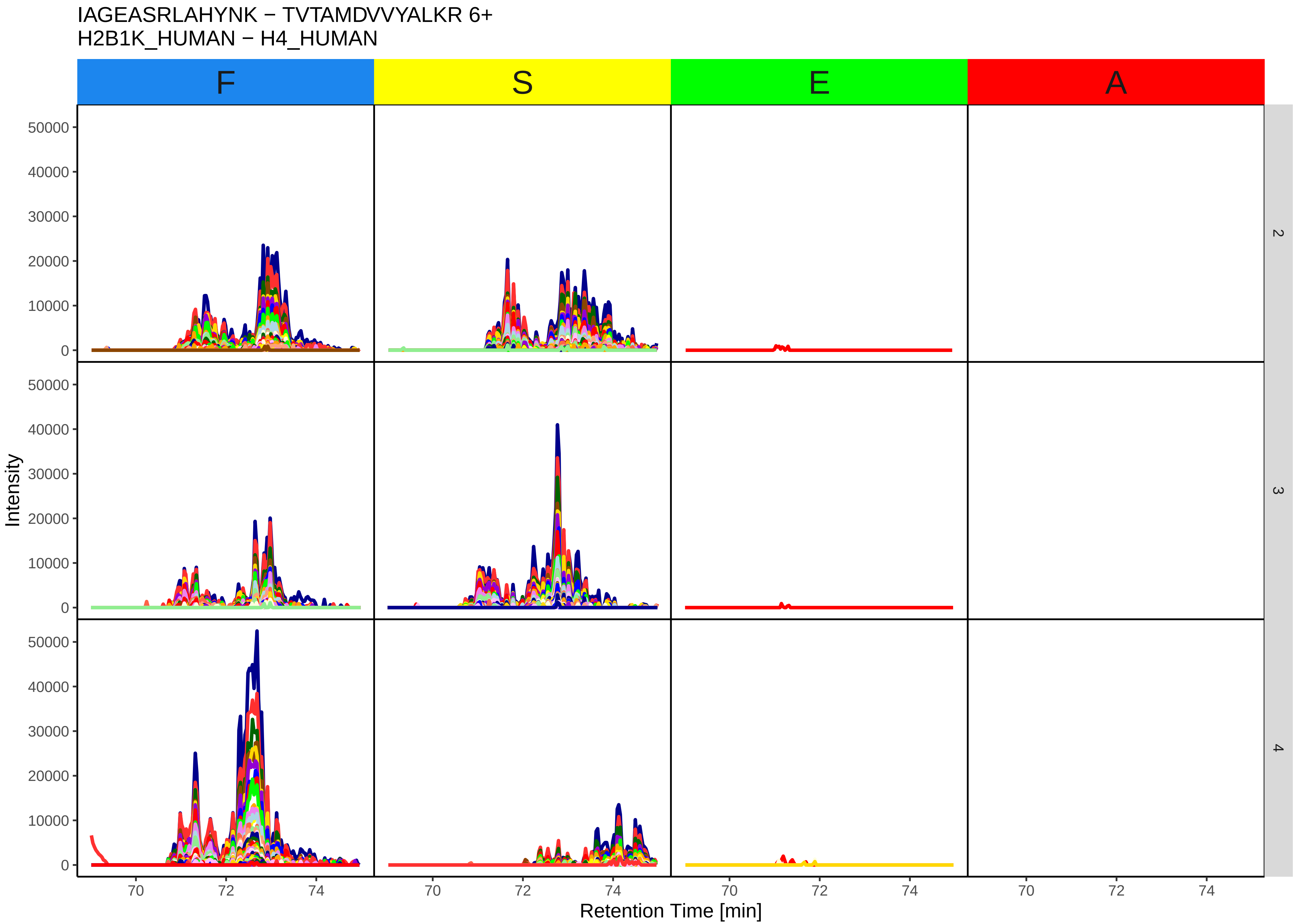

IAGEASRLAHYNK − TVTAMDVVYALKR 6+
H2B1K_HUMAN − H4_HUMAN
F
S
E
A
2
3
4
Intensity
0
10000
20000
30000
40000
50000
70
72
74
Retention Time [min]

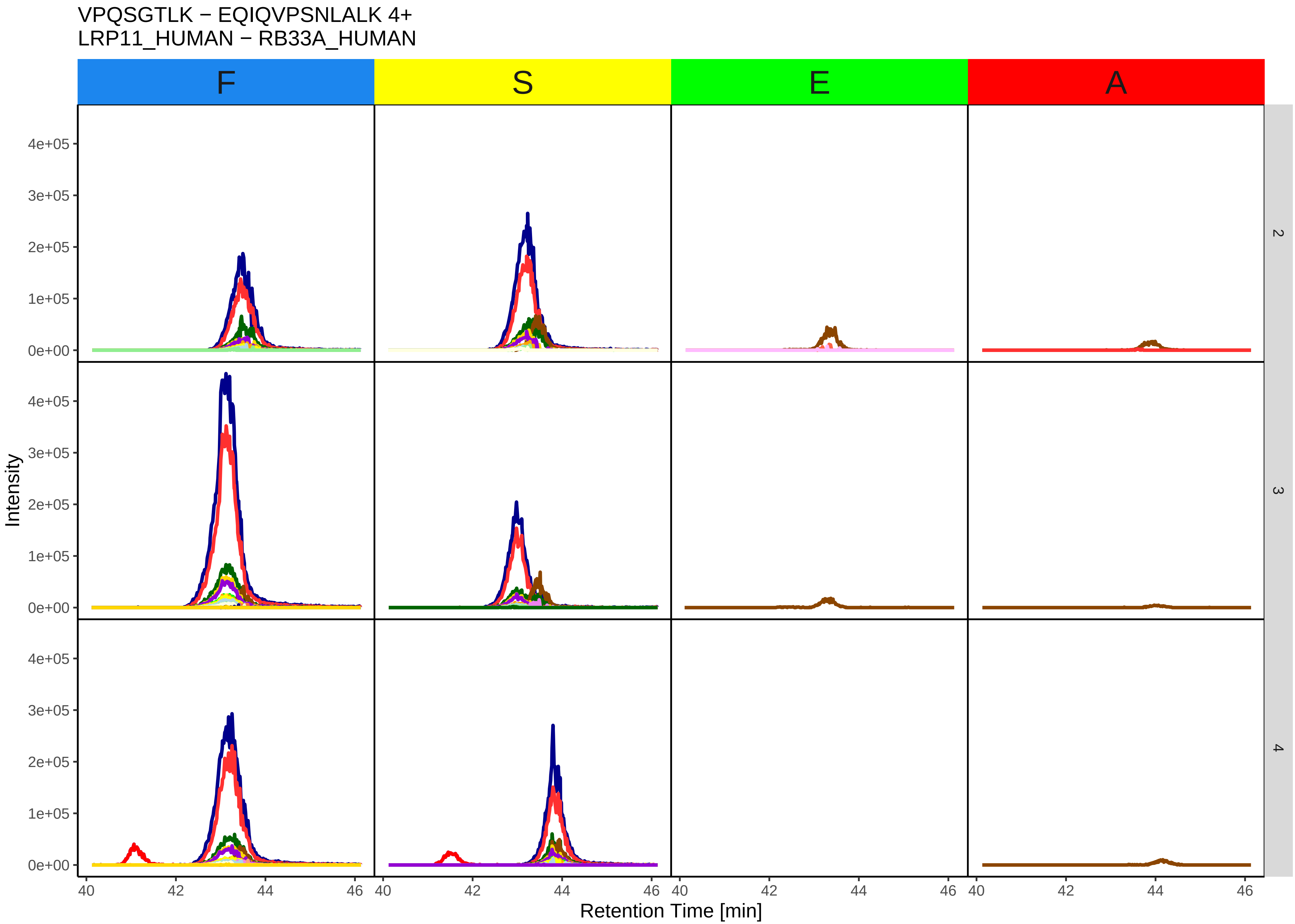
VPQSGTLK − EQIQVPSNLALK 4+
LRP11_HUMAN − RB33A_HUMAN
F
S
E
A
2
3
4
Intensity
4e+05
3e+05
2e+05
1e+05
0e+00
40
42
44
46
Retention Time [min]

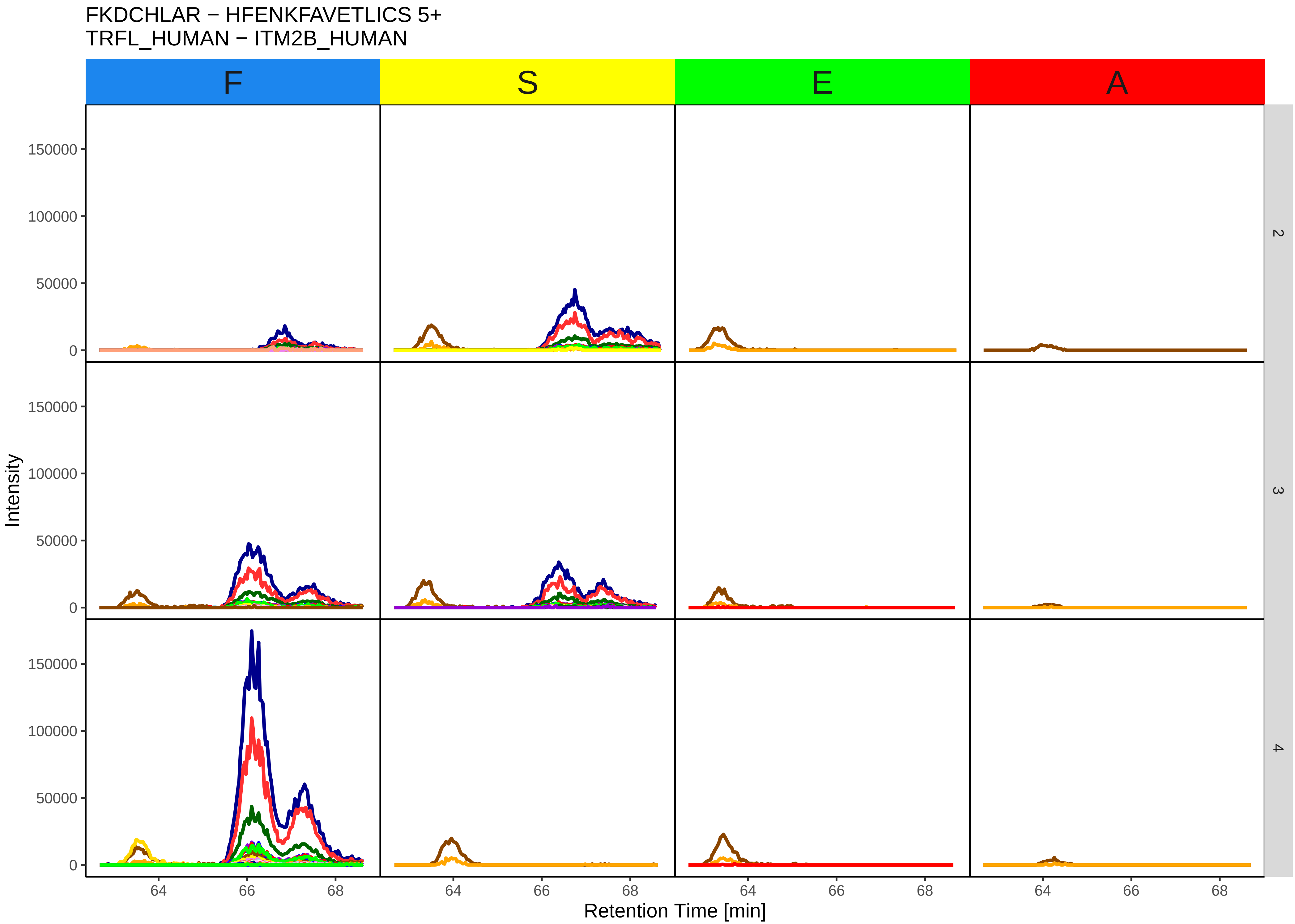
FKDCHLAR − HFENKFAVETLICS 5+
TRFL_HUMAN − ITM2B_HUMAN
F
S
E
A
2
3
4
Intensity
150000
100000
50000
0
64
66
68
Retention Time [min]

IAGEASRLAHYNK − NSLLTDIIAAYQR 4+
H2B1K_HUMAN − AFG32_HUMAN

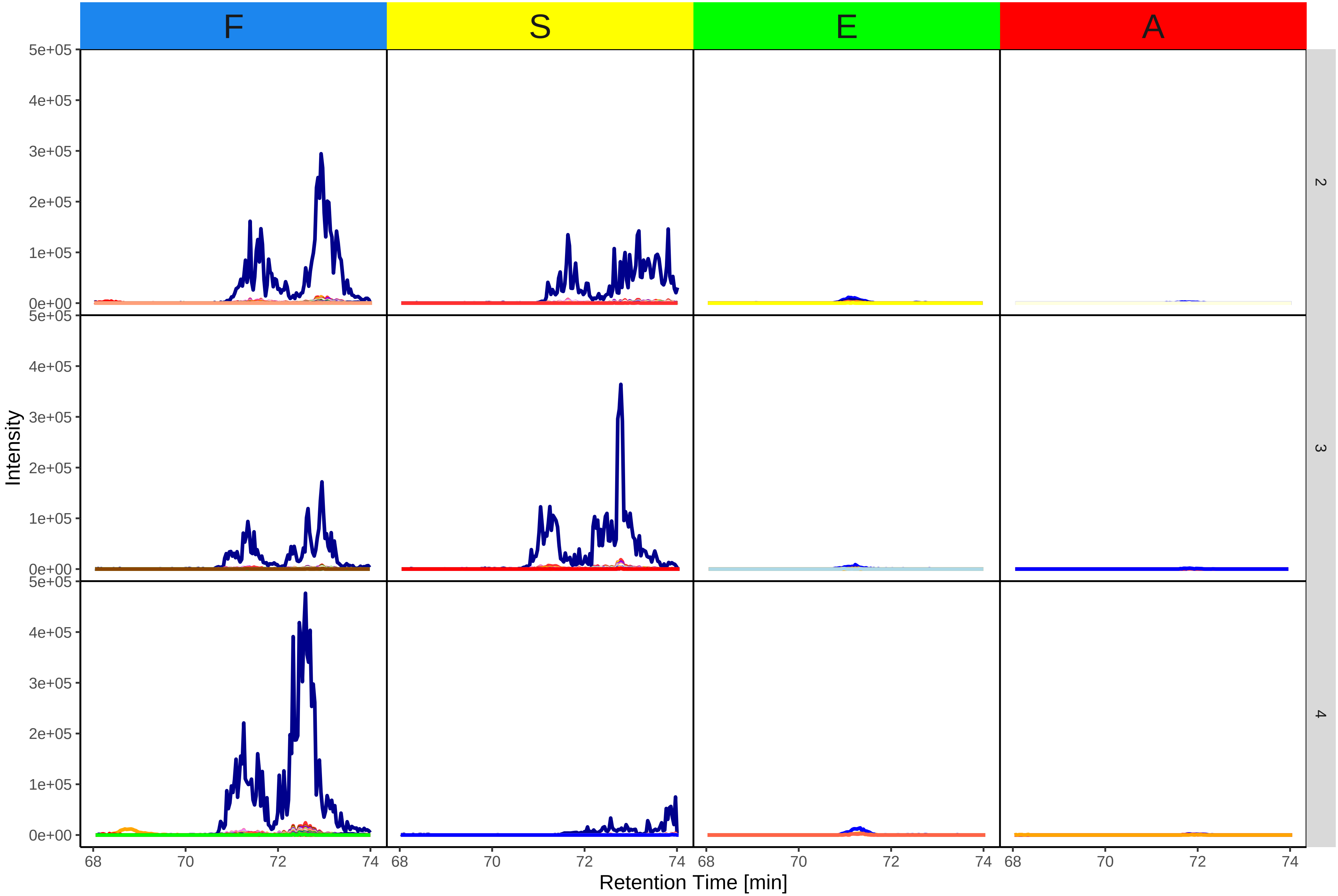

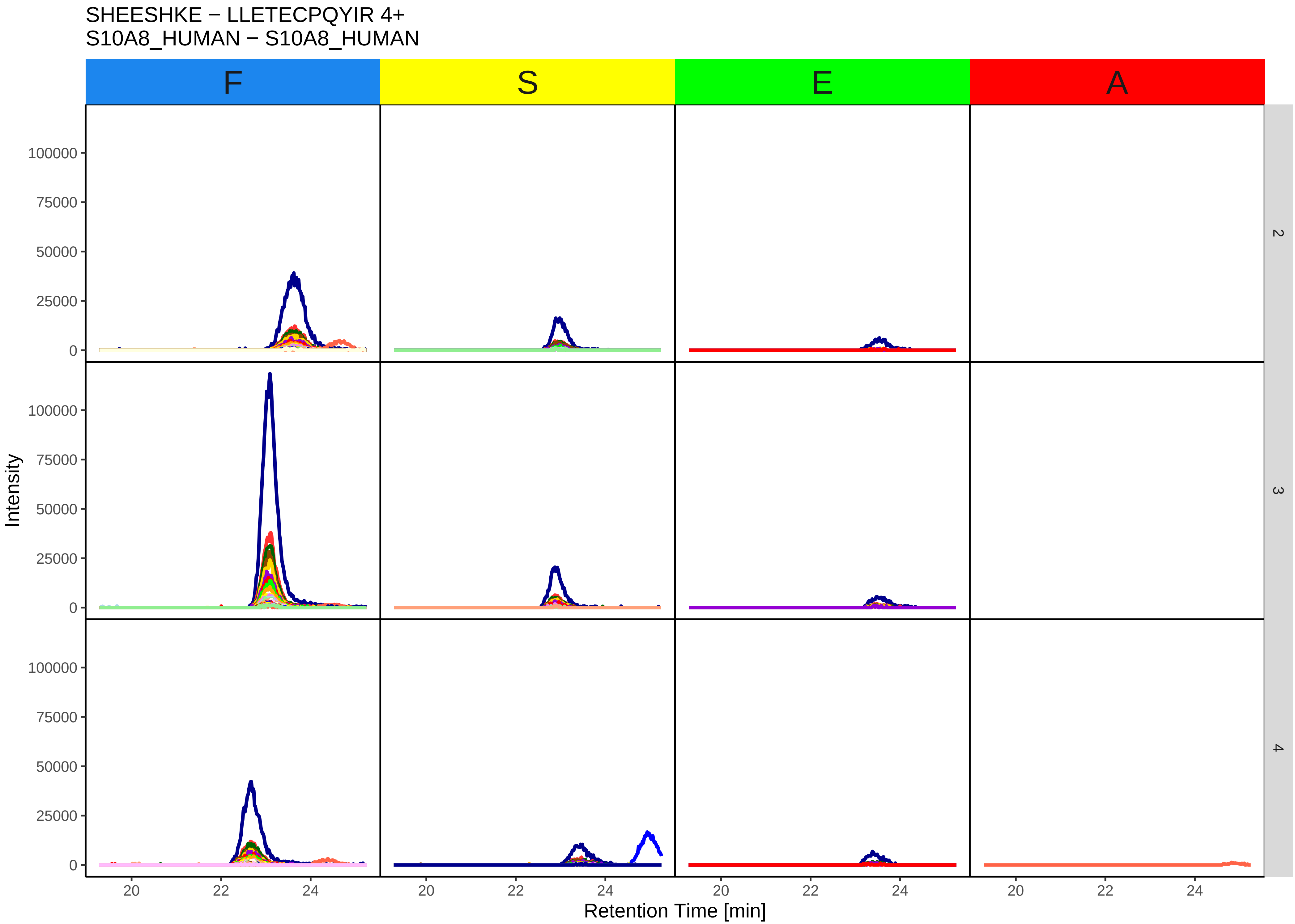

SHEESHKE − LLETECPQYIR 4+
S10A8_HUMAN − S10A8_HUMAN
F
S
E
A
2
3
4
100000
75000
50000
25000
0
Intensity
20
22
24
Retention Time [min]

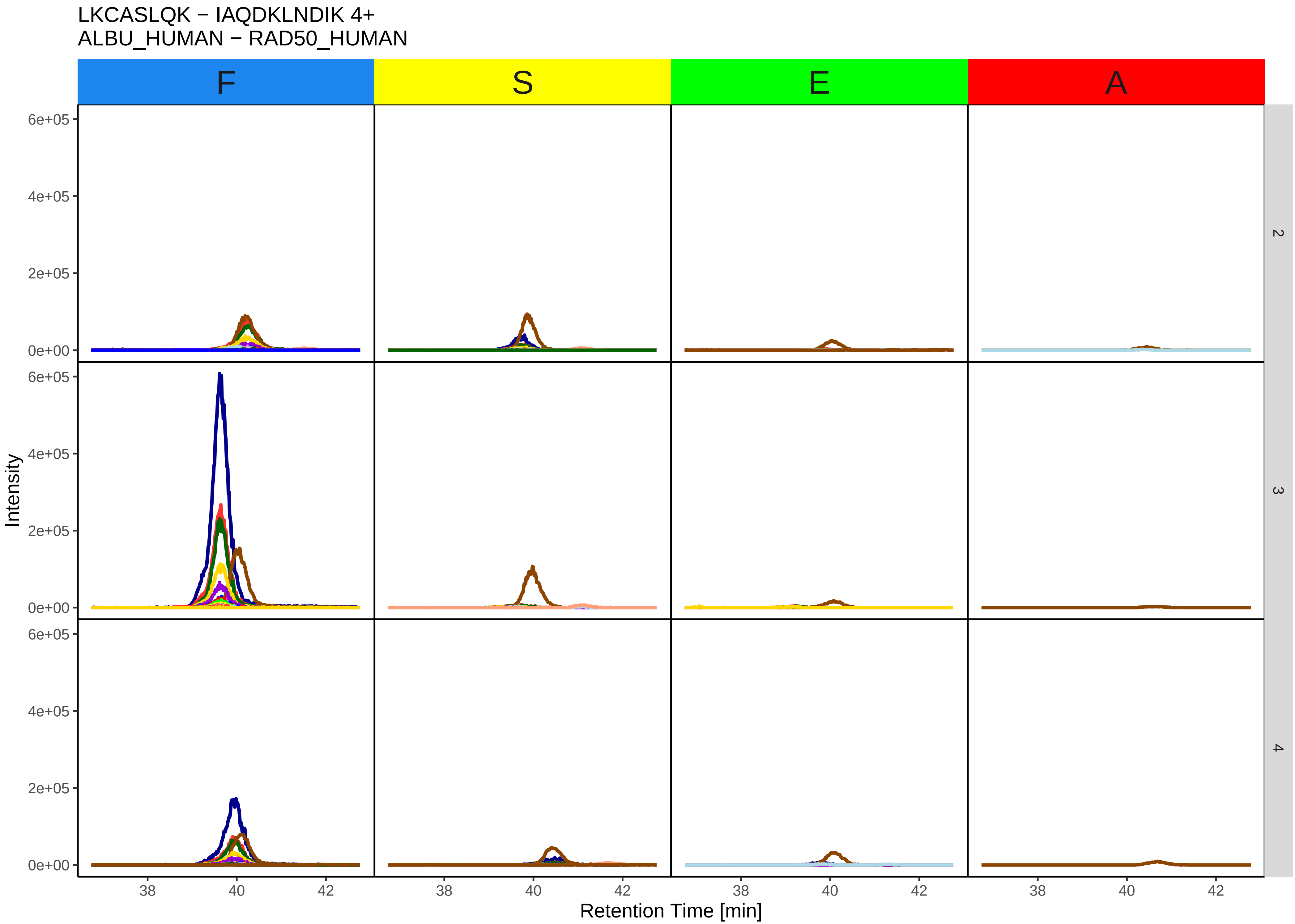

LKCASLQK − IAQDKLNDIK 4+
ALBU_HUMAN − RAD50_HUMAN
F
S
E
A
2
3
4
Intensity
6e+05
4e+05
2e+05
0e+00
38
40
42
Retention Time [min]

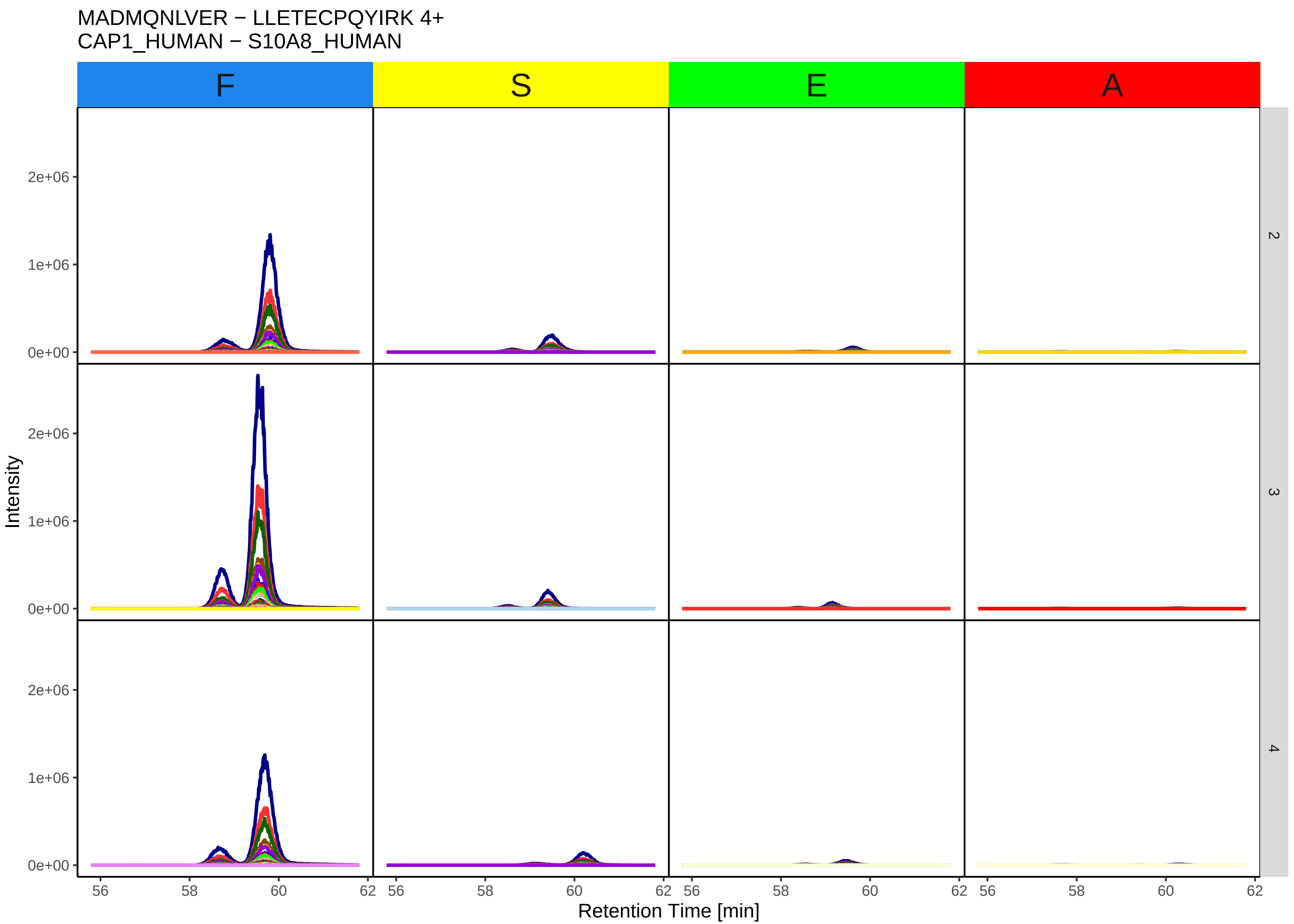
MADMQNLVER − LLETECPQYIRK 4+
CAP1_HUMAN − S10A8_HUMAN
F
S
E
A
2
3
4
Intensity
0e+00
1e+06
2e+06
56
58
60
62
Retention Time [min]

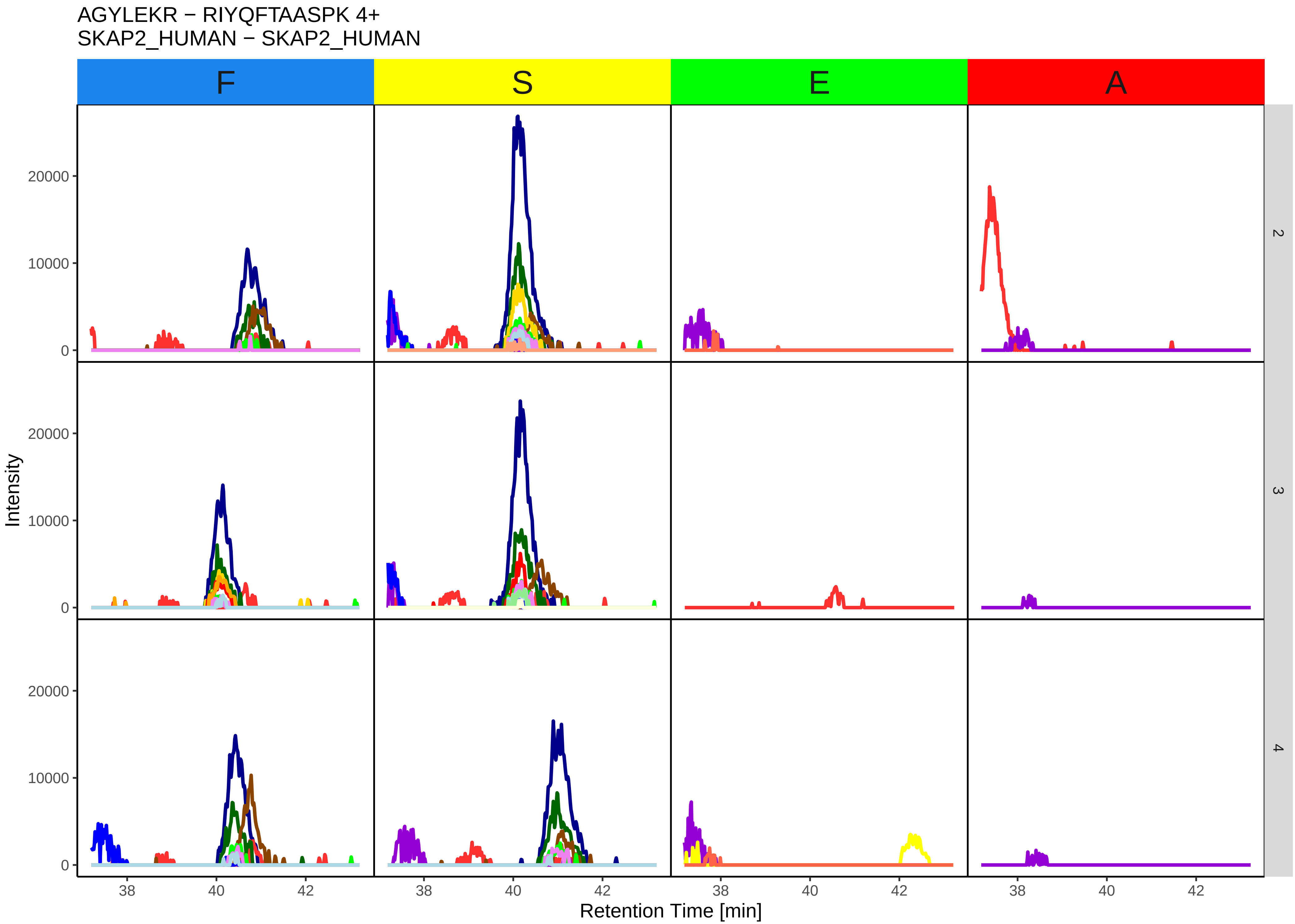
AGYLEKR − RIYQFTAASPK 4+
SKAP2_HUMAN − SKAP2_HUMAN
F
S
E
A
2
3
4
Intensity
20000
10000
0
38
40
42
Retention Time [min]

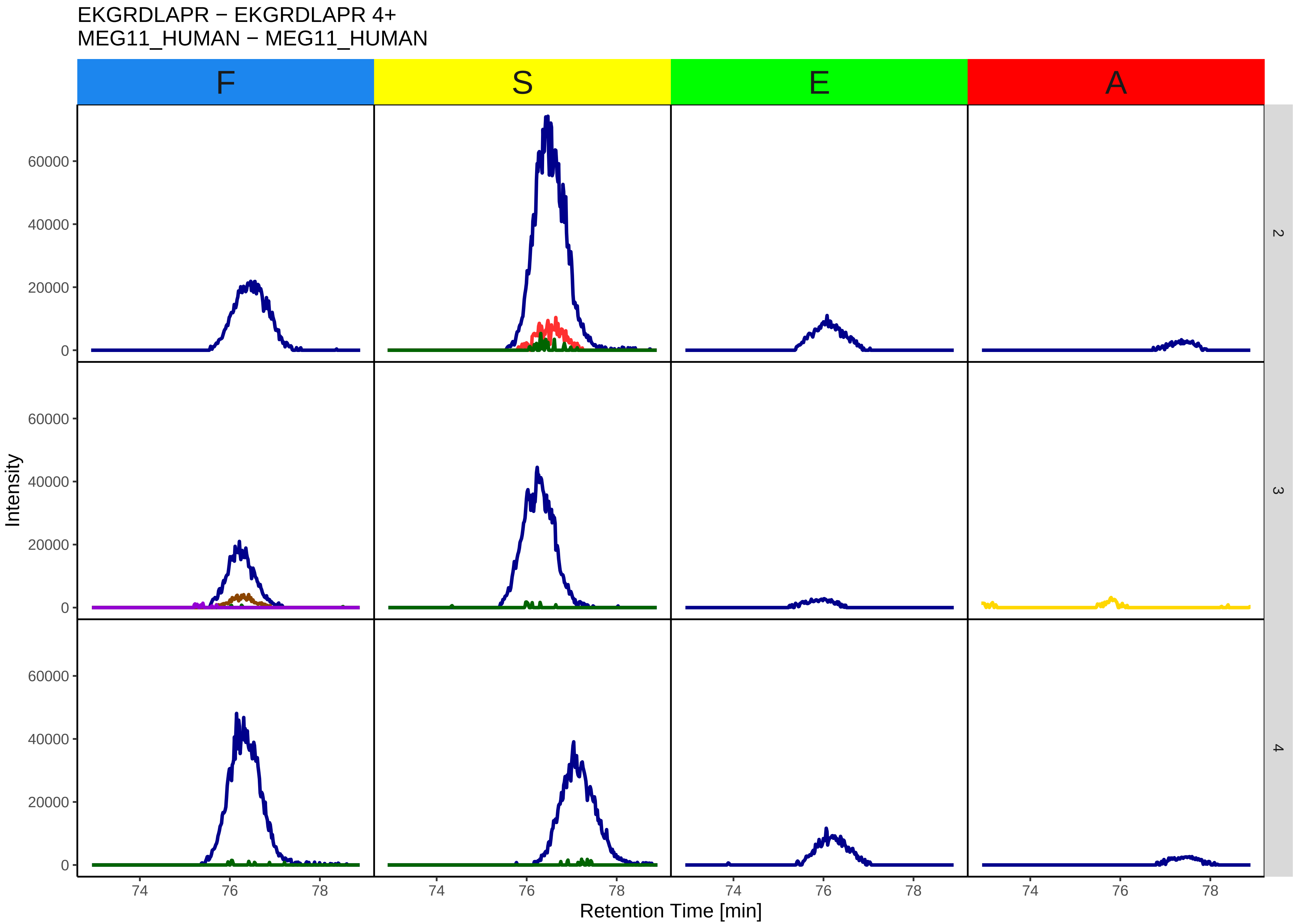

EKGRDLAPR − EKGRDLAPR 4+
MEG11_HUMAN − MEG11_HUMAN
F
S
E
A
2
3
4
60000
40000
20000
0
Intensity
74
76
78
Retention Time [min]

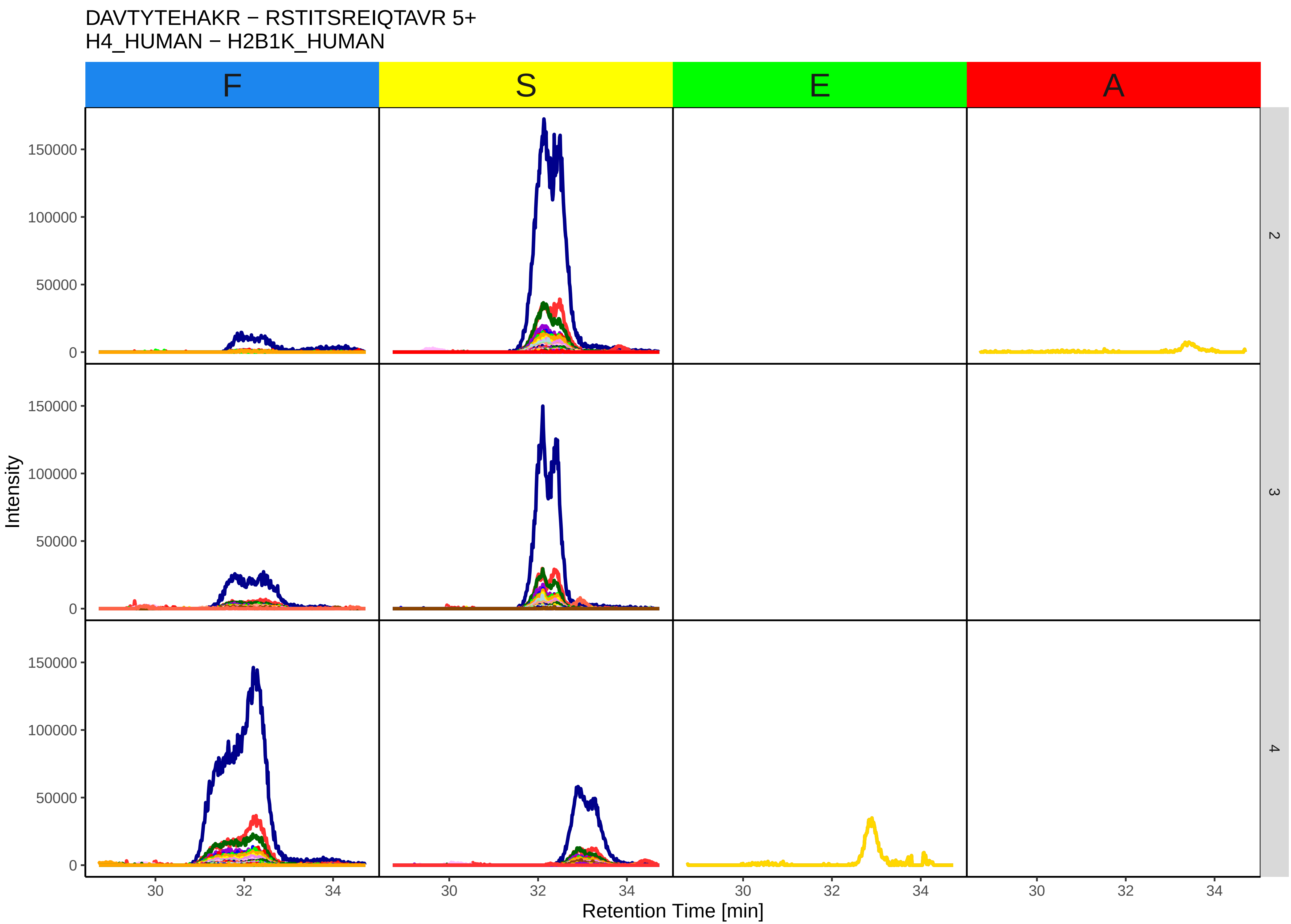
DAVTYTEHAKR − RSTITSREIQTAVR 5+
H4_HUMAN − H2B1K_HUMAN
F
S
E
A
2
3
4
150000
100000
50000
0
Intensity
30
32
34
Retention Time [min]

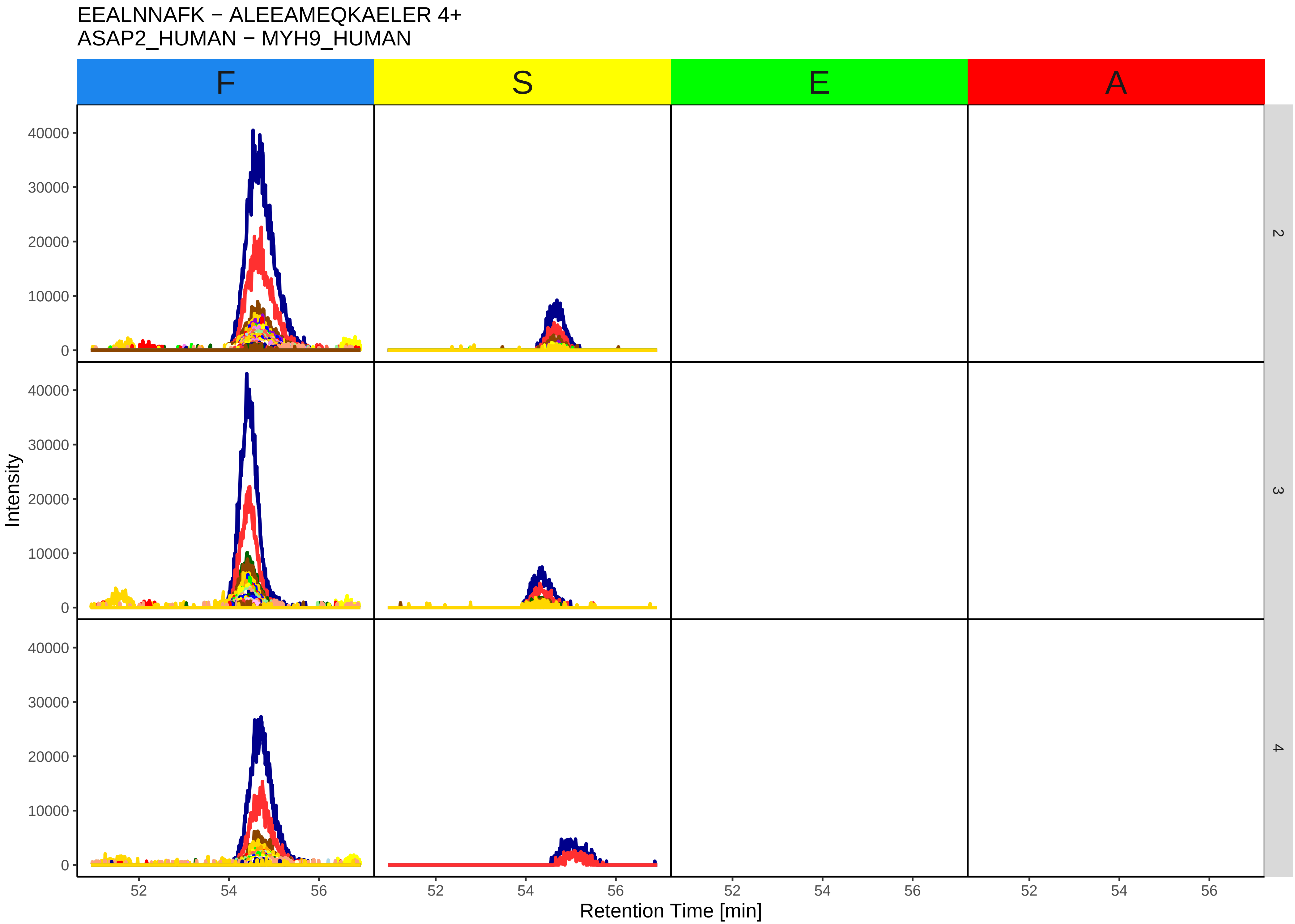
EEALNNAFK − ALEEAMEQKAELER 4+
ASAP2_HUMAN − MYH9_HUMAN
F
S
E
A
2
3
4
Intensity
40000
30000
20000
10000
0
52
54
56
Retention Time [min]

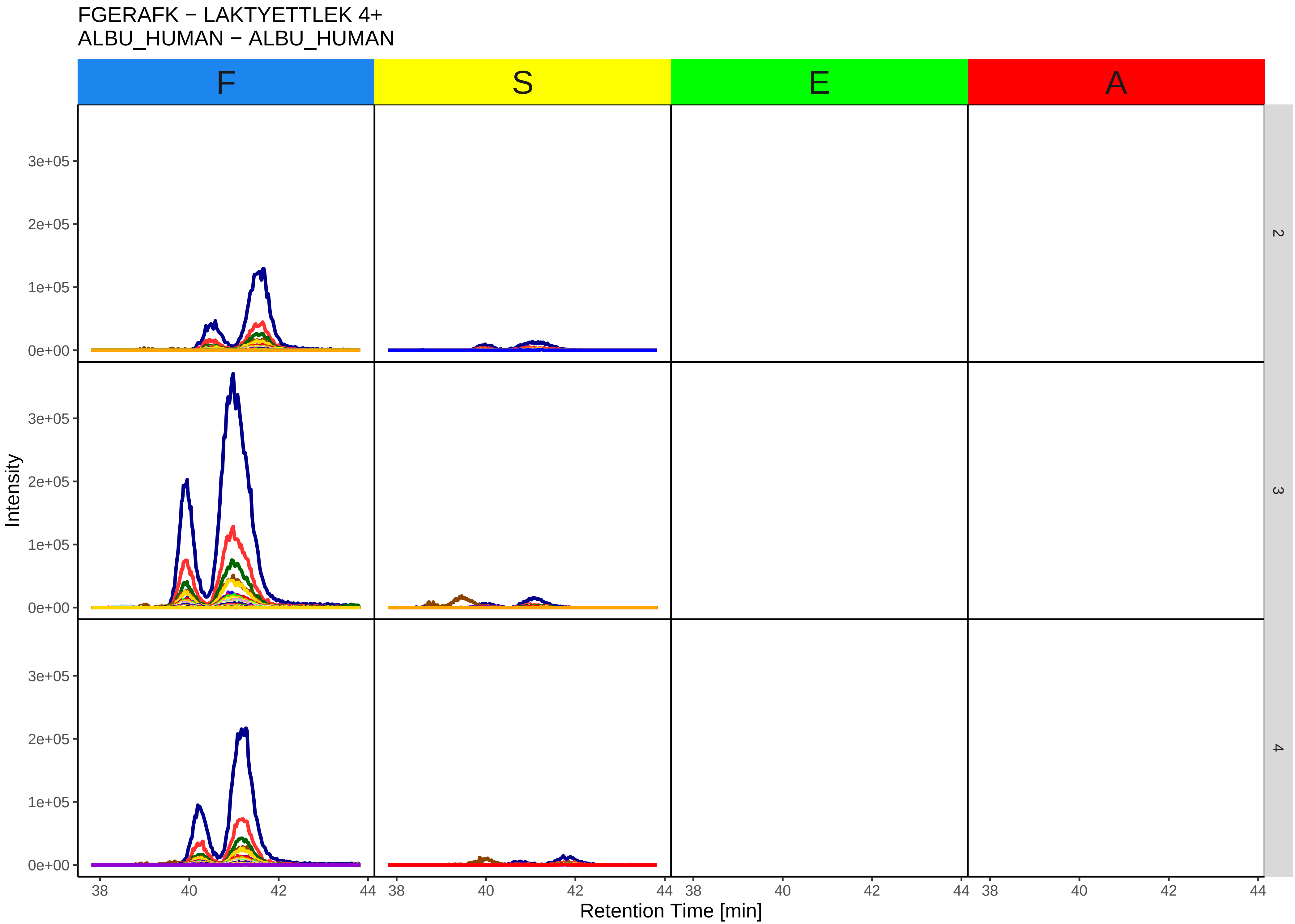
FGERAFK − LAKTYETTLEK 4+
ALBU_HUMAN − ALBU_HUMAN
F
S
E
A
2
3
4
Intensity
0e+00
1e+05
2e+05
3e+05
38
40
42
44
Retention Time [min]

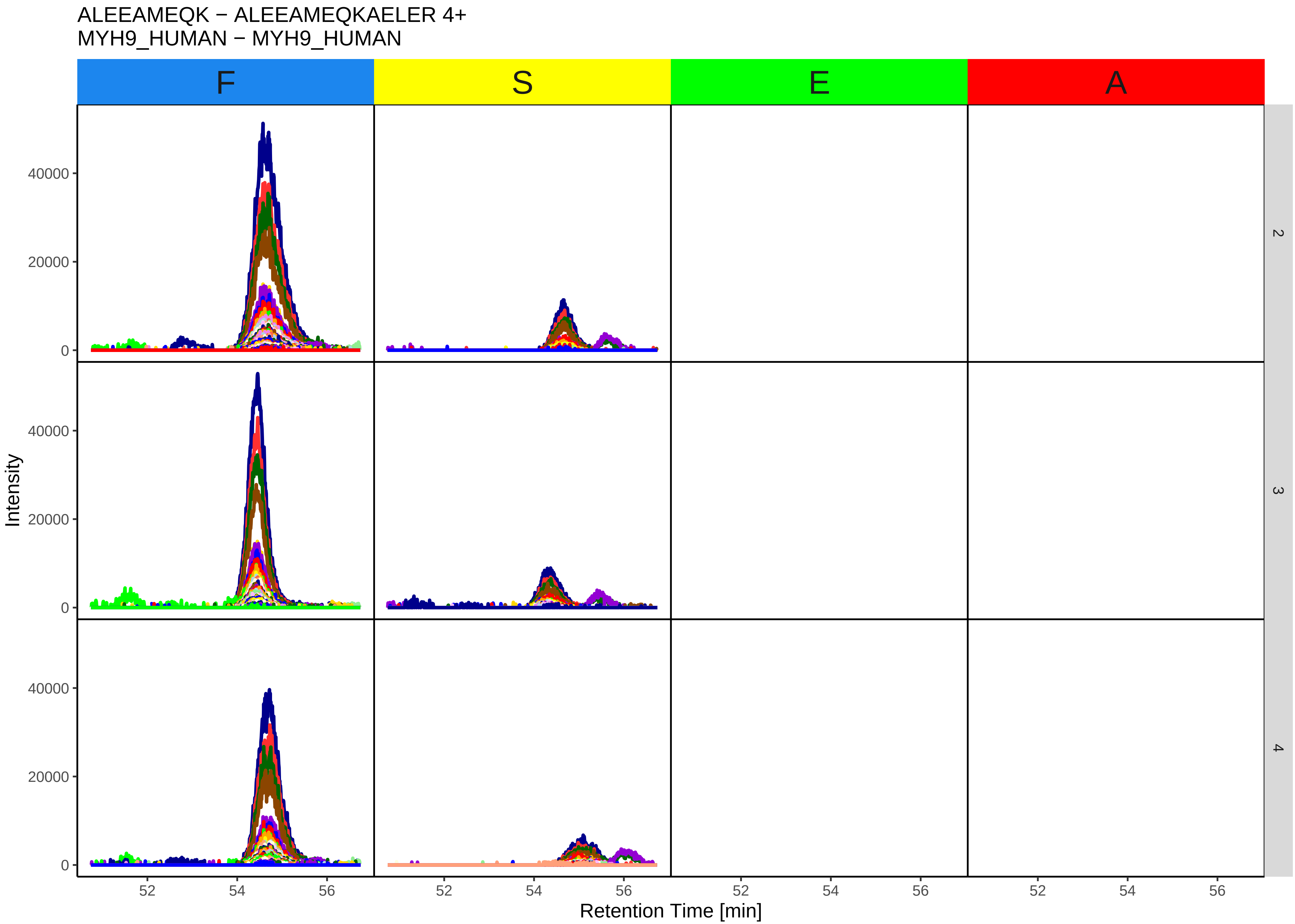
ALEEAMEQK − ALEEAMEQKAELER 4+
MYH9_HUMAN − MYH9_HUMAN
F
S
E
A
2
3
4
Intensity
0
20000
40000
52
54
56
Retention Time [min]

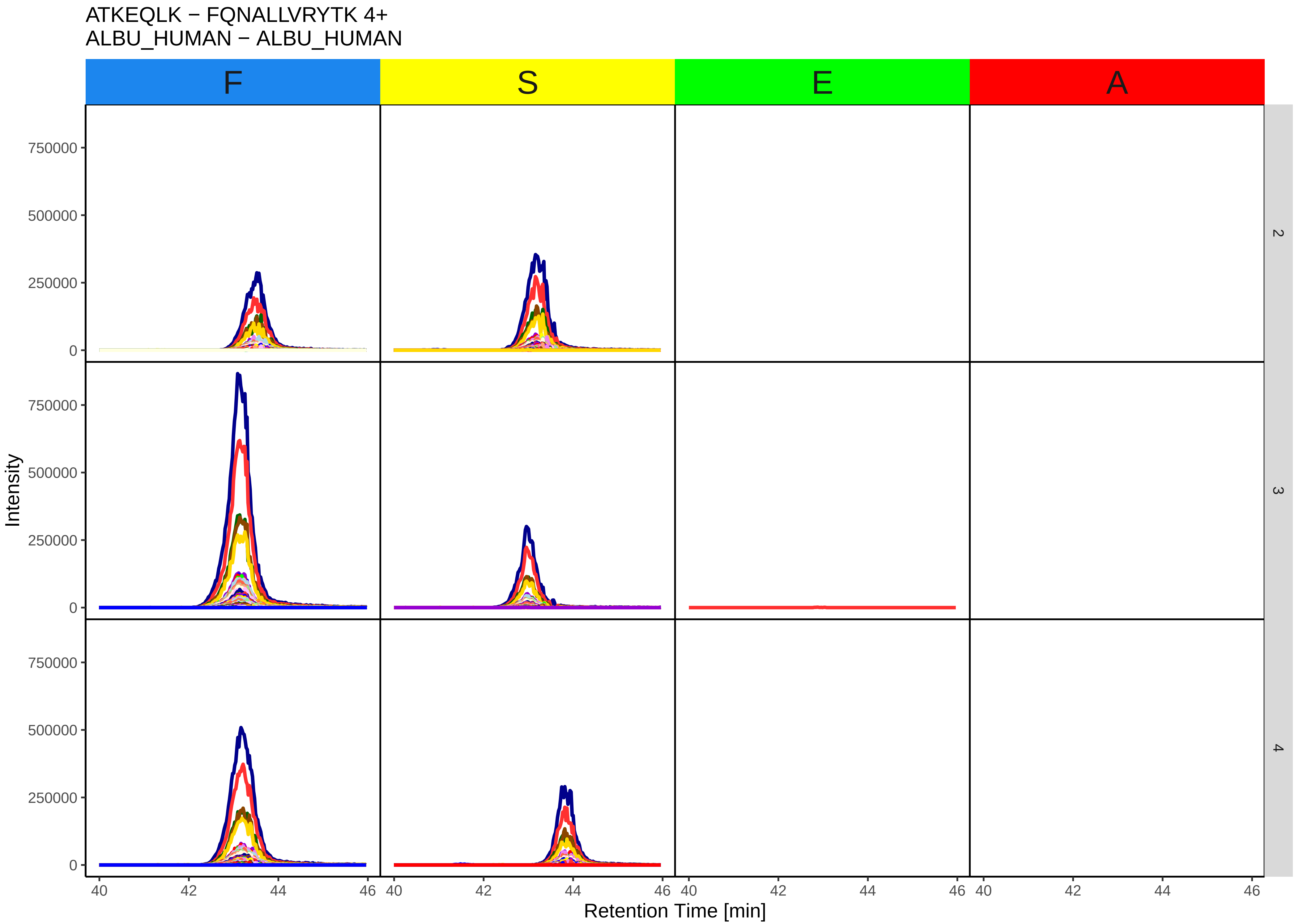
ATKEQLK − FQNALLVRYTK 4+
ALBU_HUMAN − ALBU_HUMAN
F
S
E
A
2
3
4
Intensity
750000
500000
250000
0
40
42
44
46
Retention Time [min]

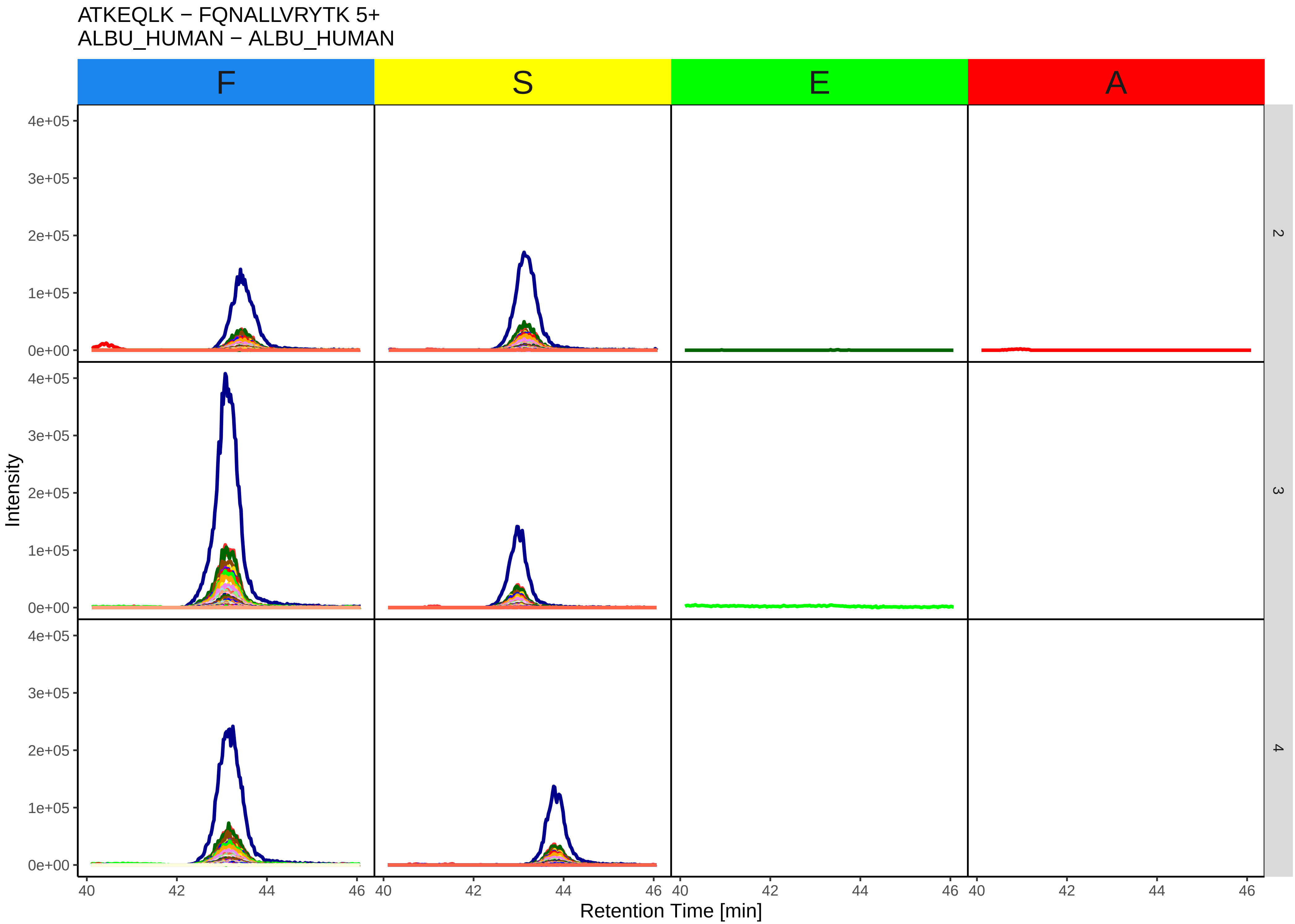
ATKEQLK − FQNALLVRYTK 5+
ALBU_HUMAN − ALBU_HUMAN
F
S
E
A
2
3
4
Intensity
4e+05
3e+05
2e+05
1e+05
0e+00
40
42
44
46
Retention Time [min]

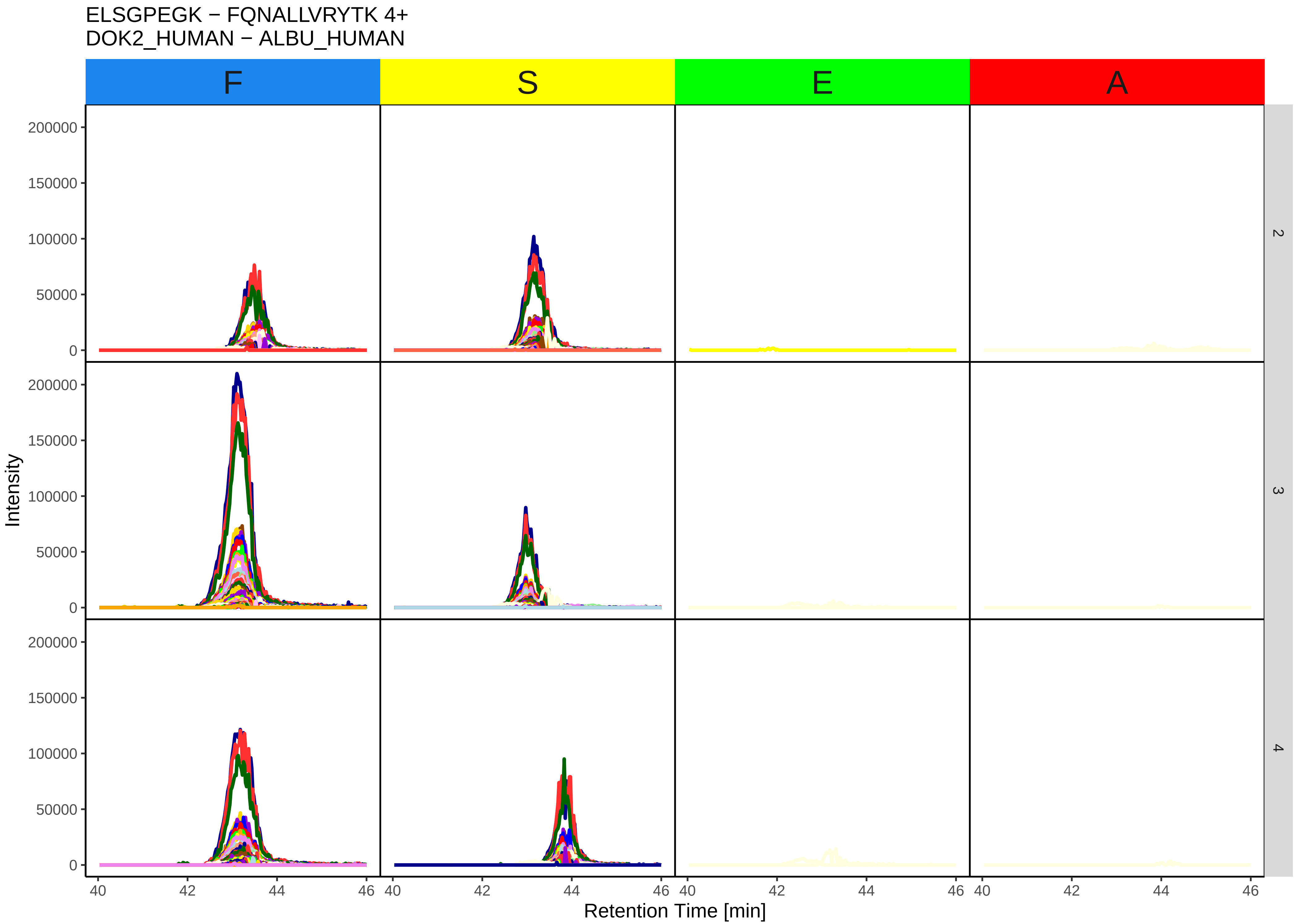
ELSGPEGK − FQNALLVRYTK 4+
DOK2_HUMAN − ALBU_HUMAN
F
S
E
A
2
3
4
Intensity
200000
150000
100000
50000
0
40
42
44
46
Retention Time [min]

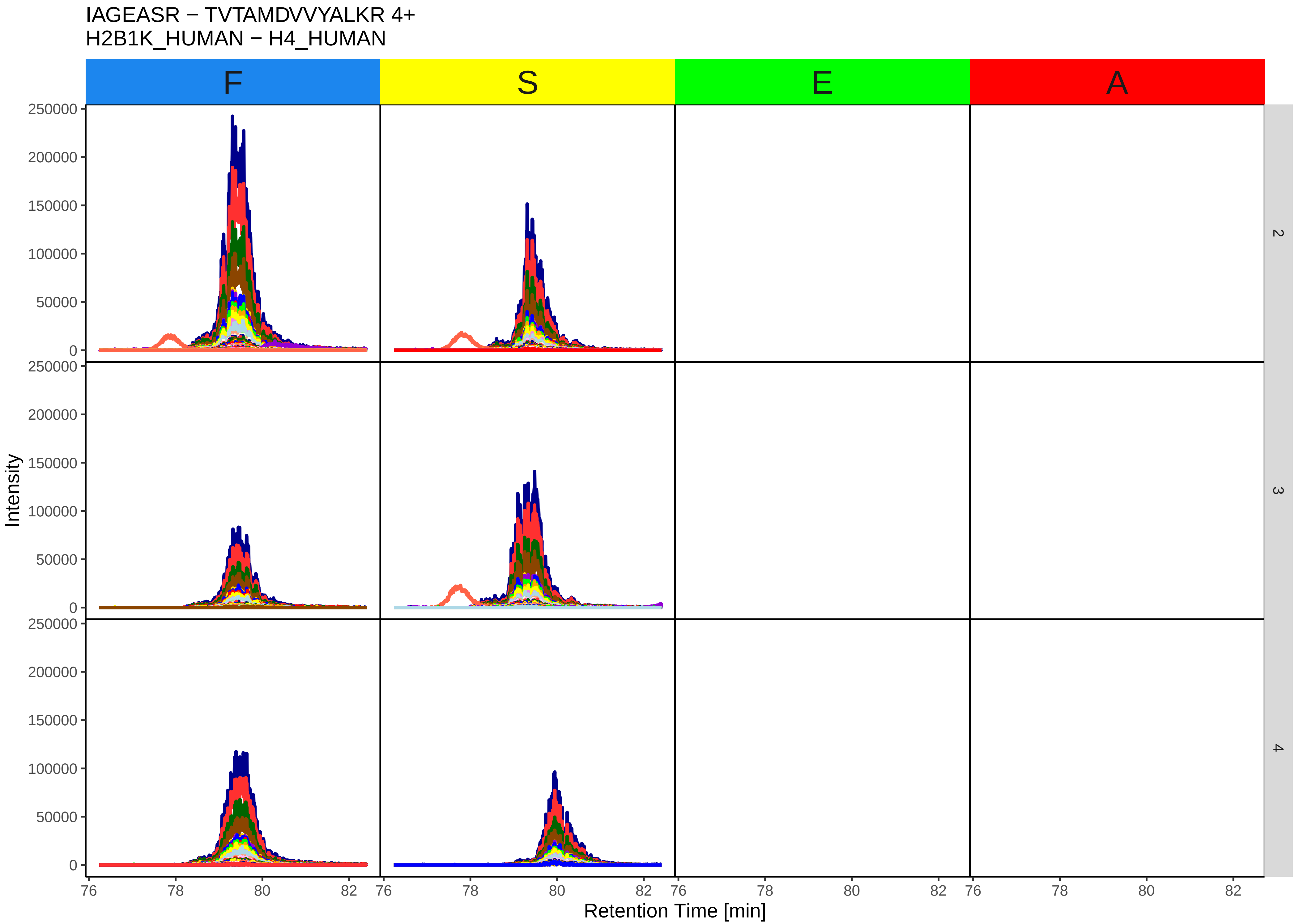

IAGEASR − TVTAMDVVYALKR 4+
H2B1K_HUMAN − H4_HUMAN
F
S
E
A
2
3
4
Intensity
250000
200000
150000
100000
50000
0
76
78
80
82
Retention Time [min]

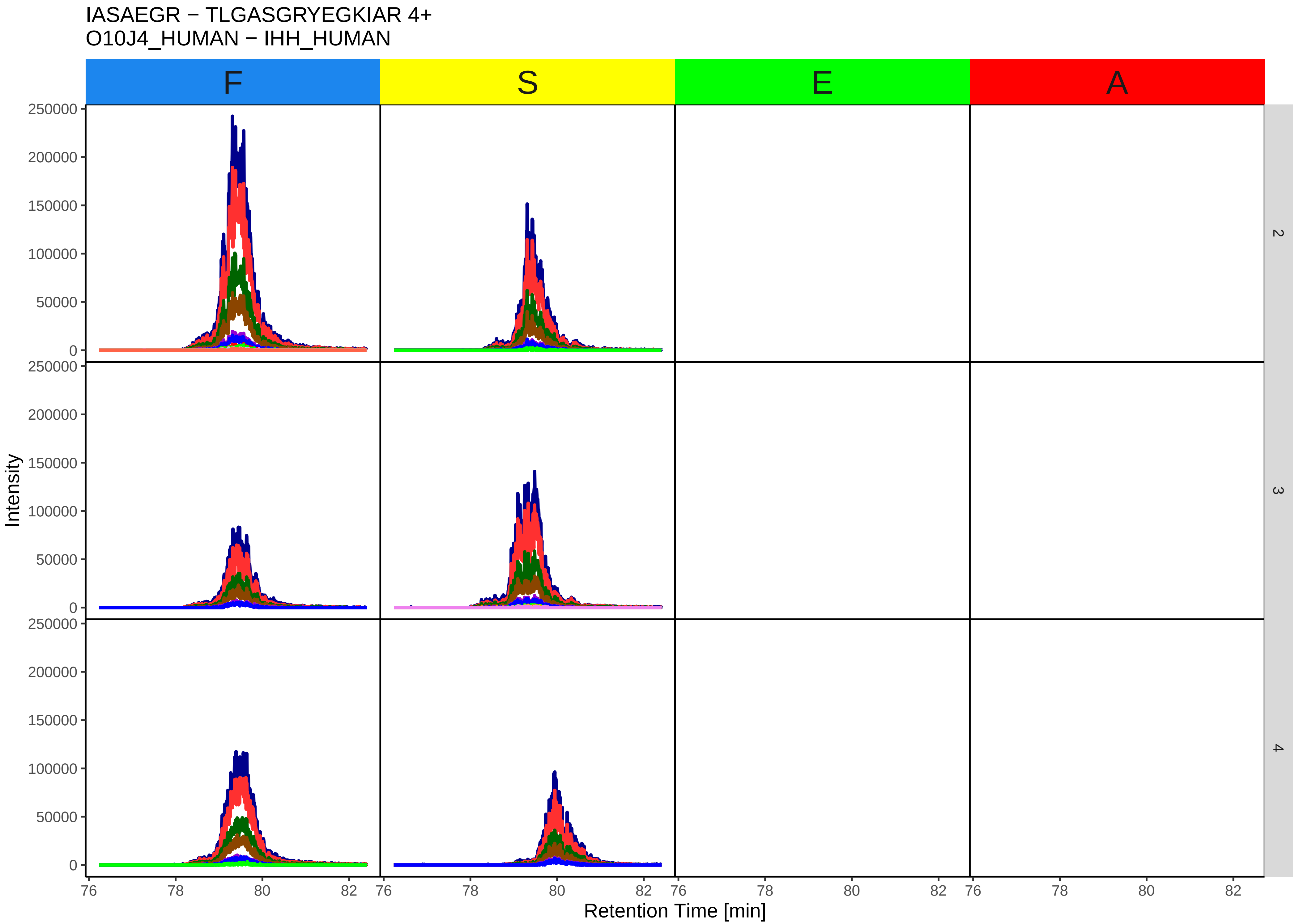
IASAEGR − TLGASGRYEGKIAR 4+
O10J4_HUMAN − IHH_HUMAN
F
S
E
A
2
3
4
Intensity
0
50000
100000
150000
200000
250000
76
78
80
82
Retention Time [min]

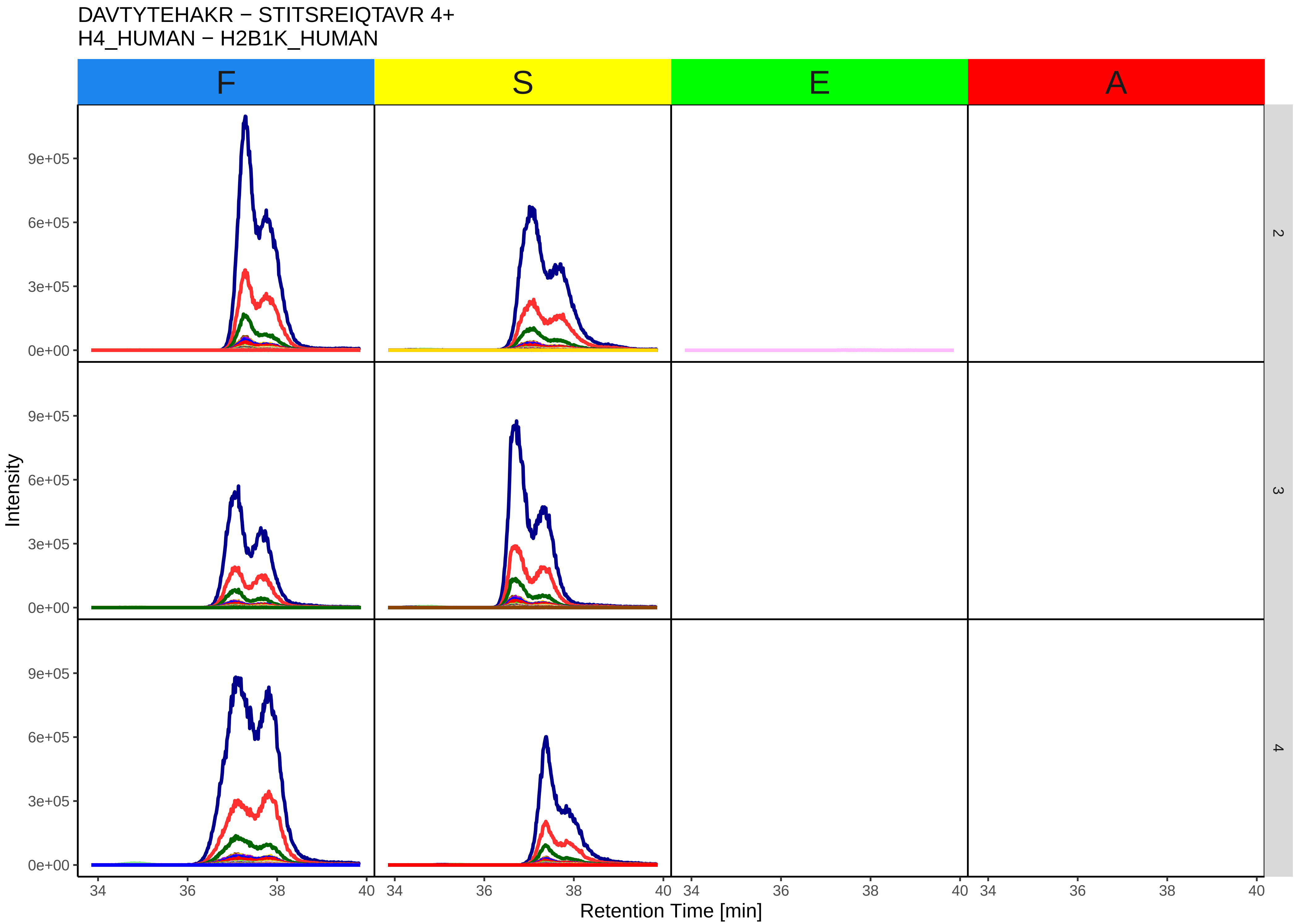
DAVTYTEHAKR − STITSREIQTAVR 4+
H4_HUMAN − H2B1K_HUMAN
F
S
E
A
2
3
4
Intensity
9e+05
6e+05
3e+05
0e+00
34
36
38
40
Retention Time [min]

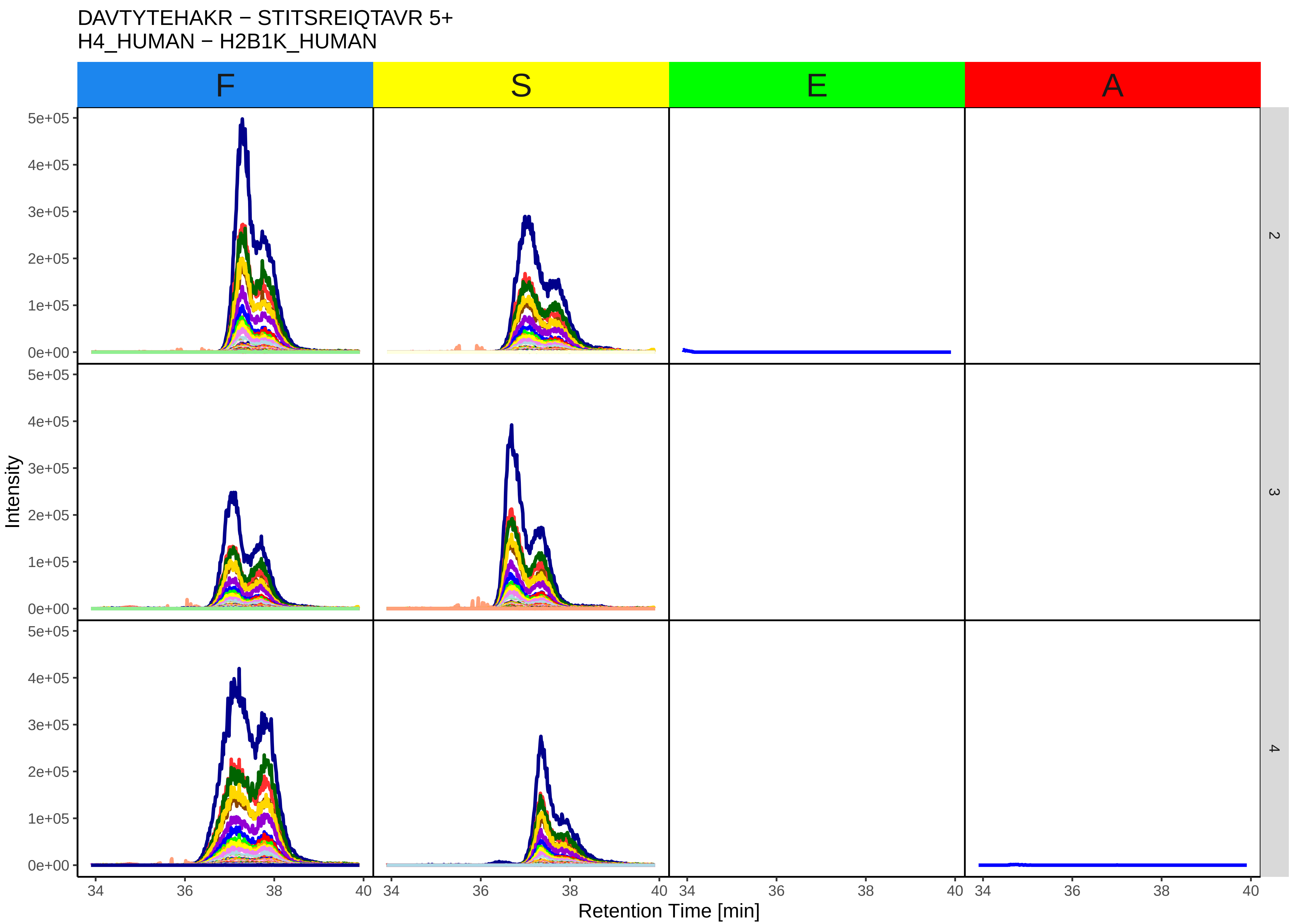
DAVTYTEHAKR − STITSREIQTAVR 5+
H4_HUMAN − H2B1K_HUMAN
F
S
E
A
2
3
4
Intensity
0e+00
1e+05
2e+05
3e+05
4e+05
5e+05
34
36
38
40
Retention Time [min]

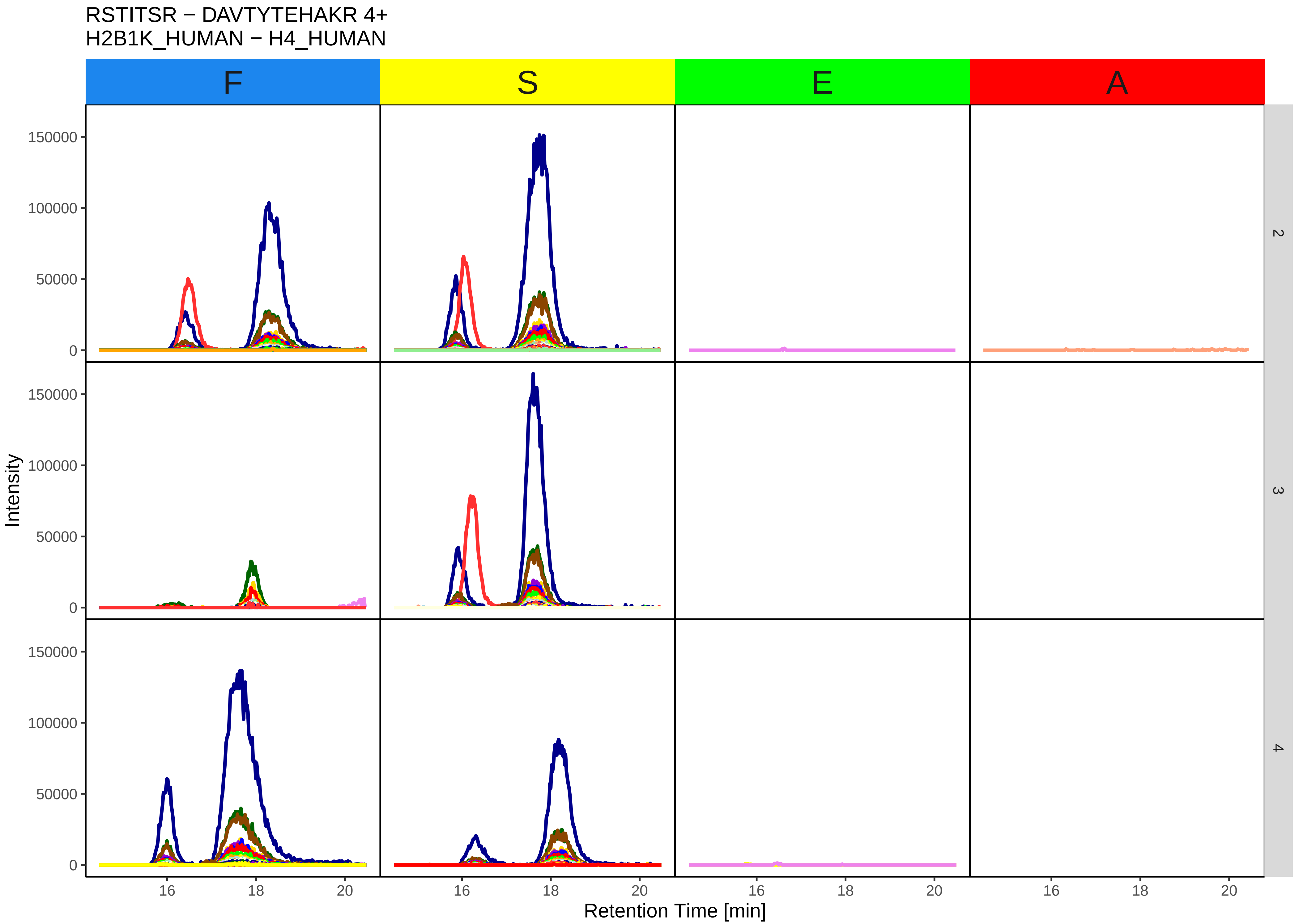
RSTITSR − DAVTYTEHAKR 4+
H2B1K_HUMAN − H4_HUMAN
F
S
E
A
2
3
4
Intensity
150000
100000
50000
0
16
18
20
Retention Time [min]

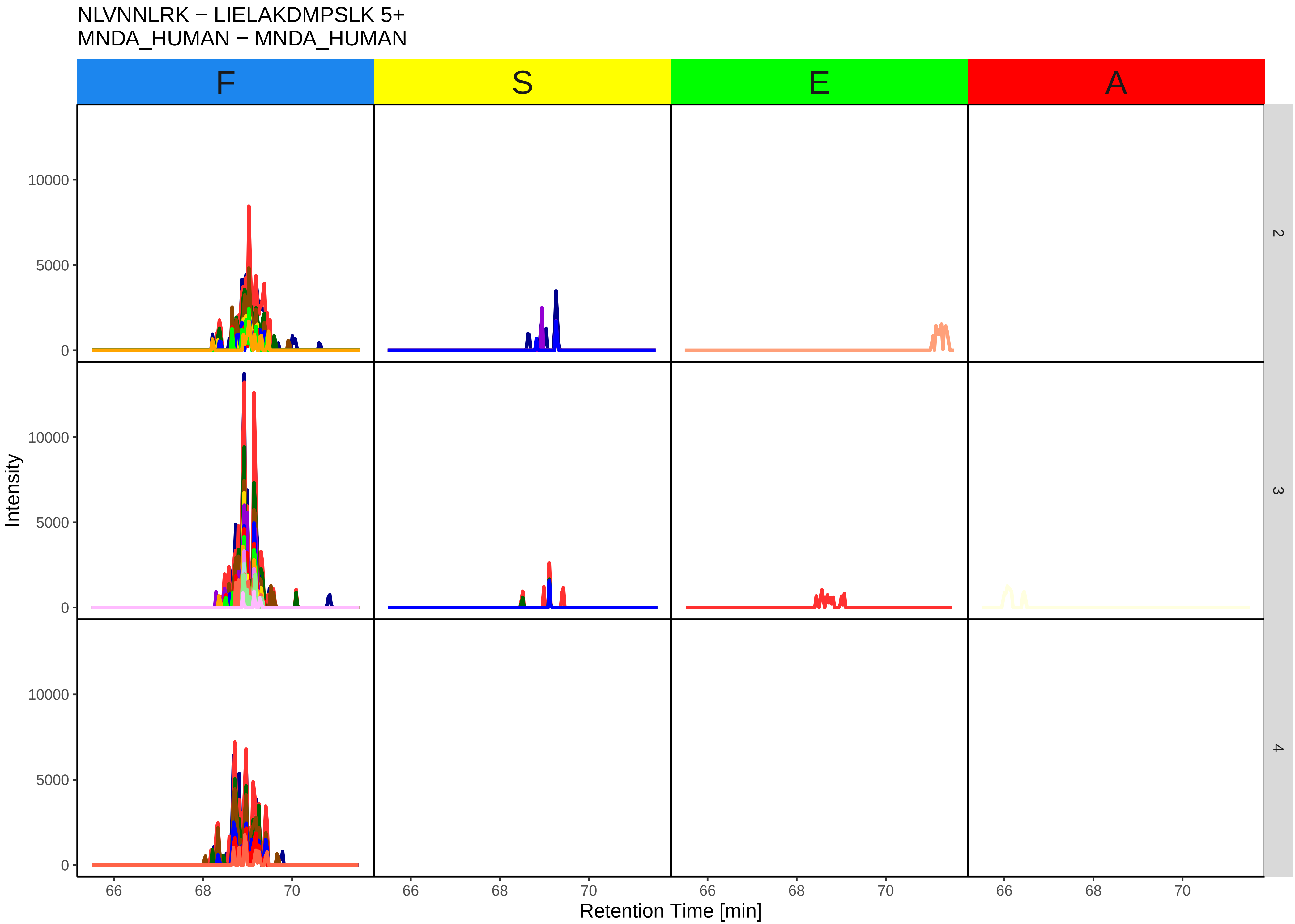
NLVNNLRK − LIELAKDMPSLK 5+
MNDA_HUMAN − MNDA_HUMAN
F
S
E
A
2
3
4
Intensity
0
5000
10000
66
68
70
Retention Time [min]

AVFPSIVGRPR − RGILTLKYPIEHGIITNWDDMEK 6+
POTEF_HUMAN − ACTH_HUMAN

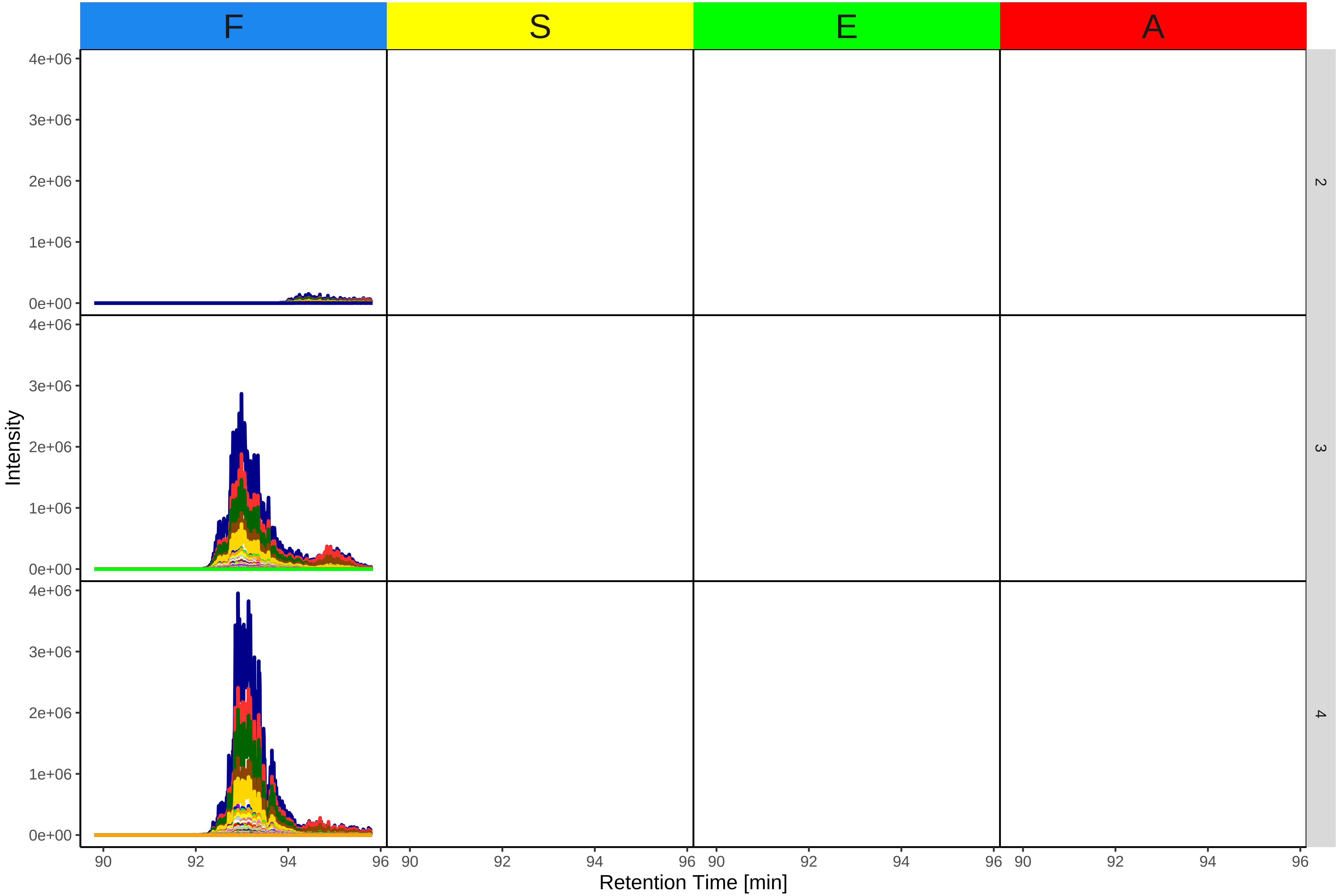

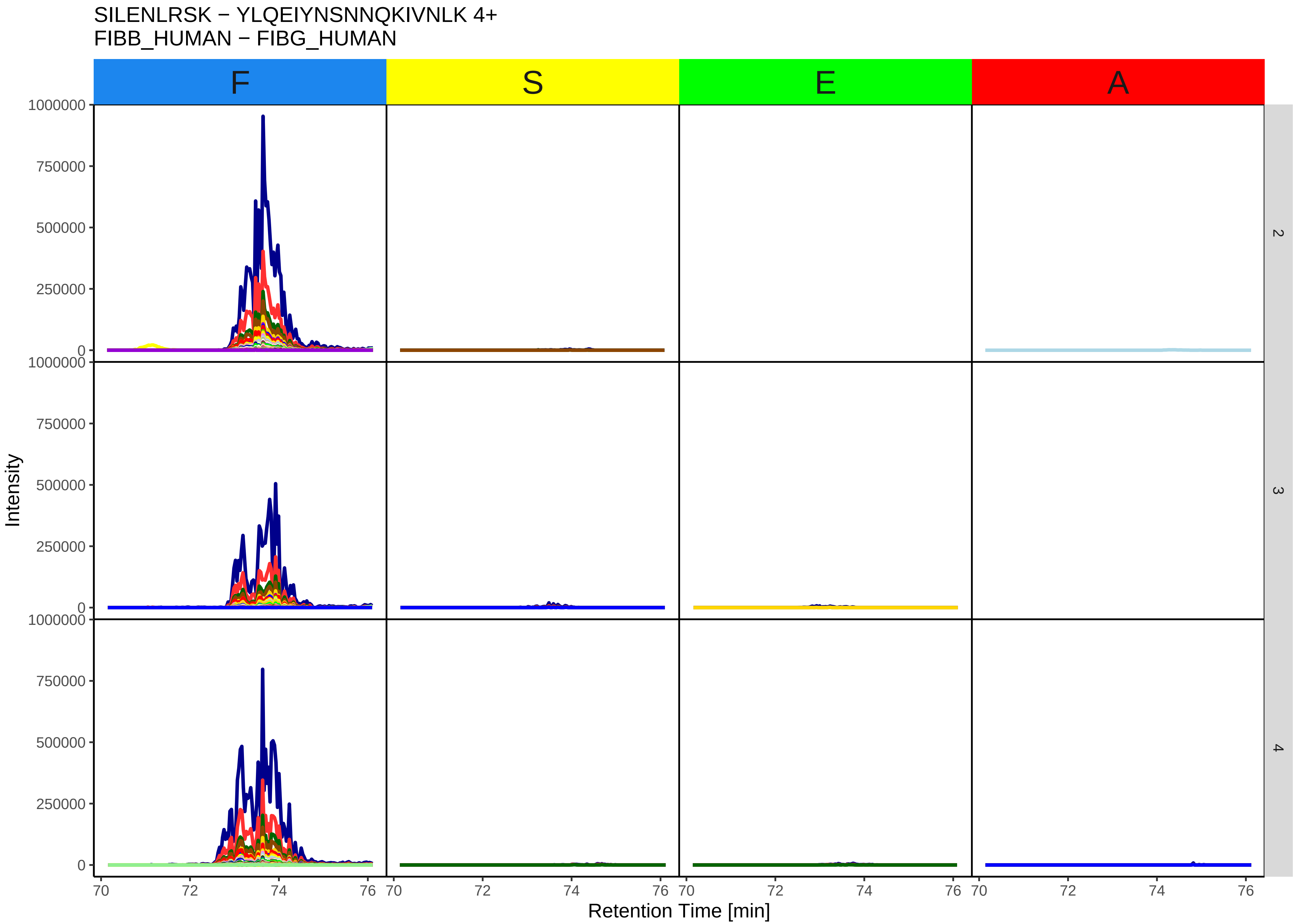
SILENLRSK − YLQEIYNSNNQKIVNLK 4+
FIBB_HUMAN − FIBG_HUMAN
F
S
E
A
1000000
750000
500000
250000
0
2
3
4
Intensity
70
72
74
76
Retention Time [min]

DAQIFIQKK − NFTEVHPDYGSHIQALLDK 4+
CATA_HUMAN − CATA_HUMAN

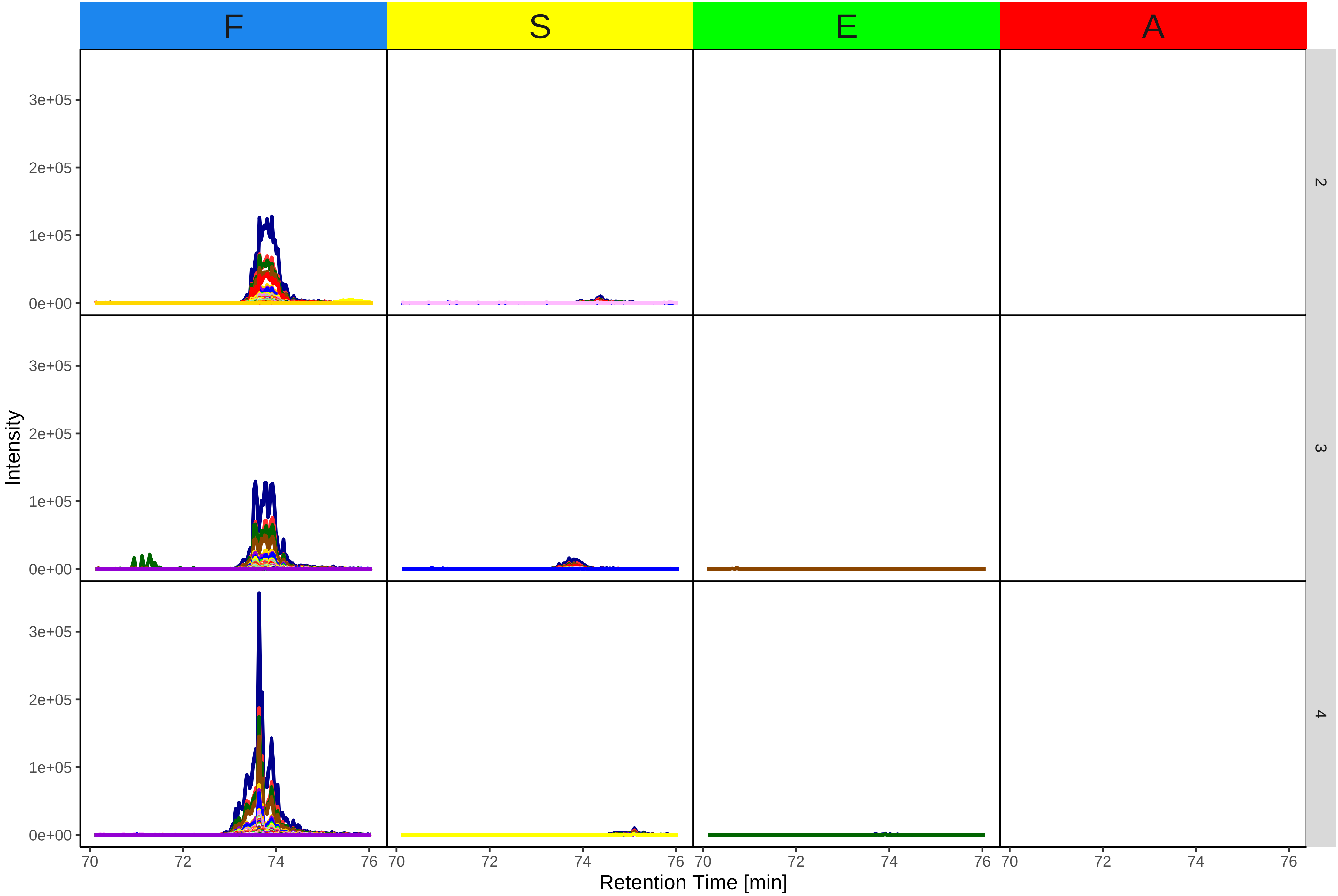

DAQIFIQKK − NFTEVHPDYGSHIQALLDK 5+
CATA_HUMAN − CATA_HUMAN

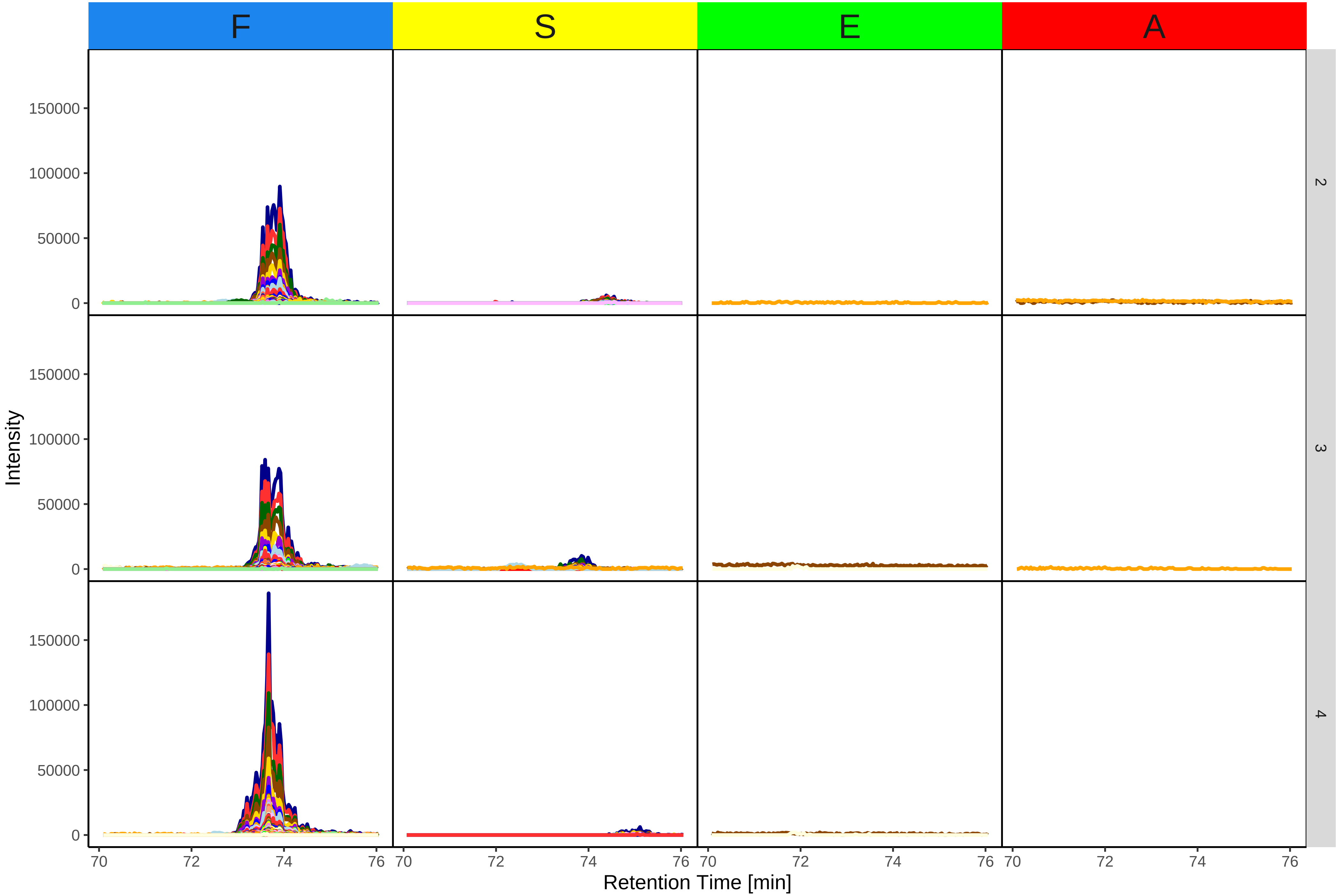

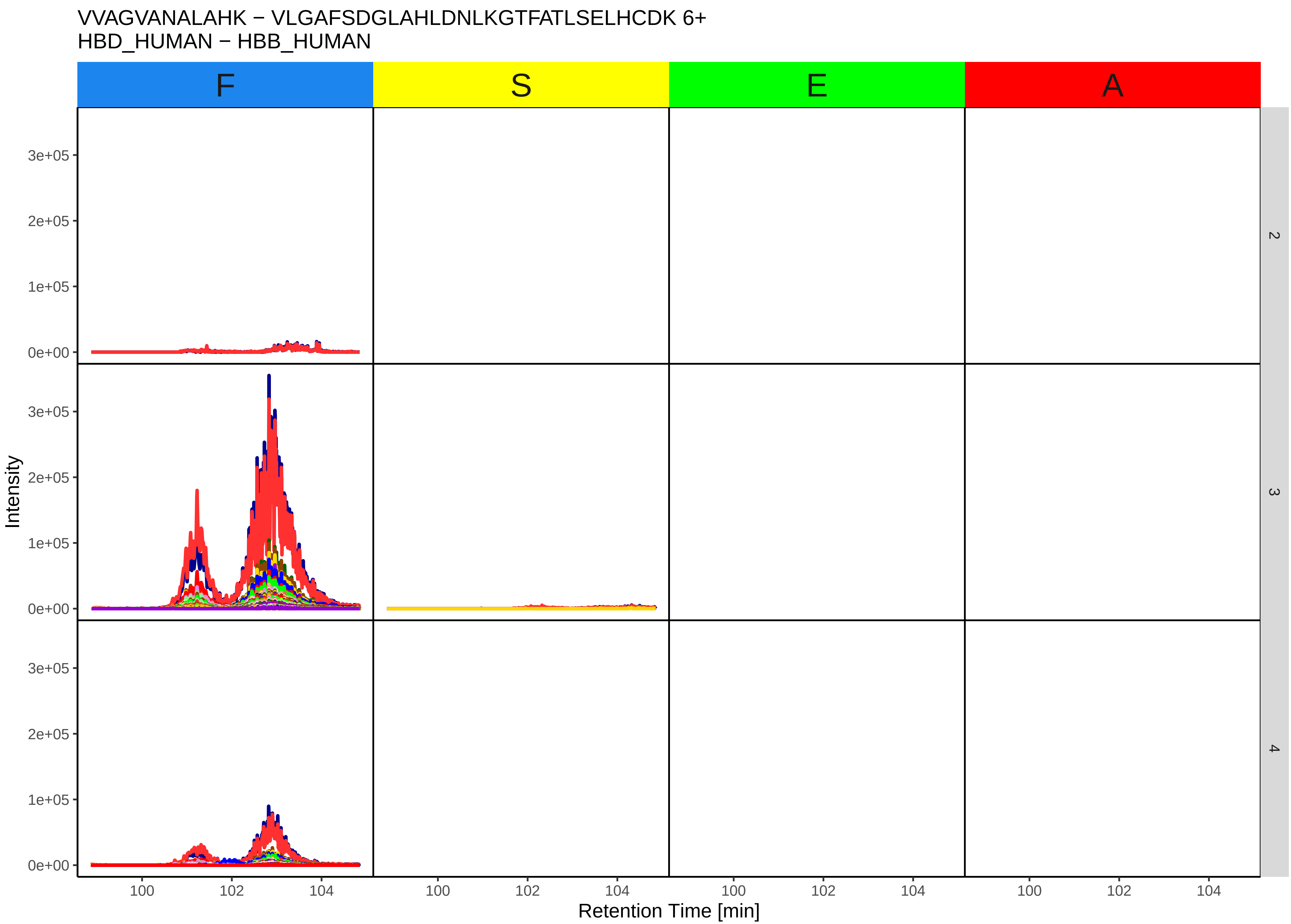
VVAGVANALAHK − VLGAFSDGLAHLDNLKGTFATLSELHCDK 6+
HBD_HUMAN − HBB_HUMAN
F
S
E
A
2
3
4
Intensity
0e+00
1e+05
2e+05
3e+05
100
102
104
Retention Time [min]

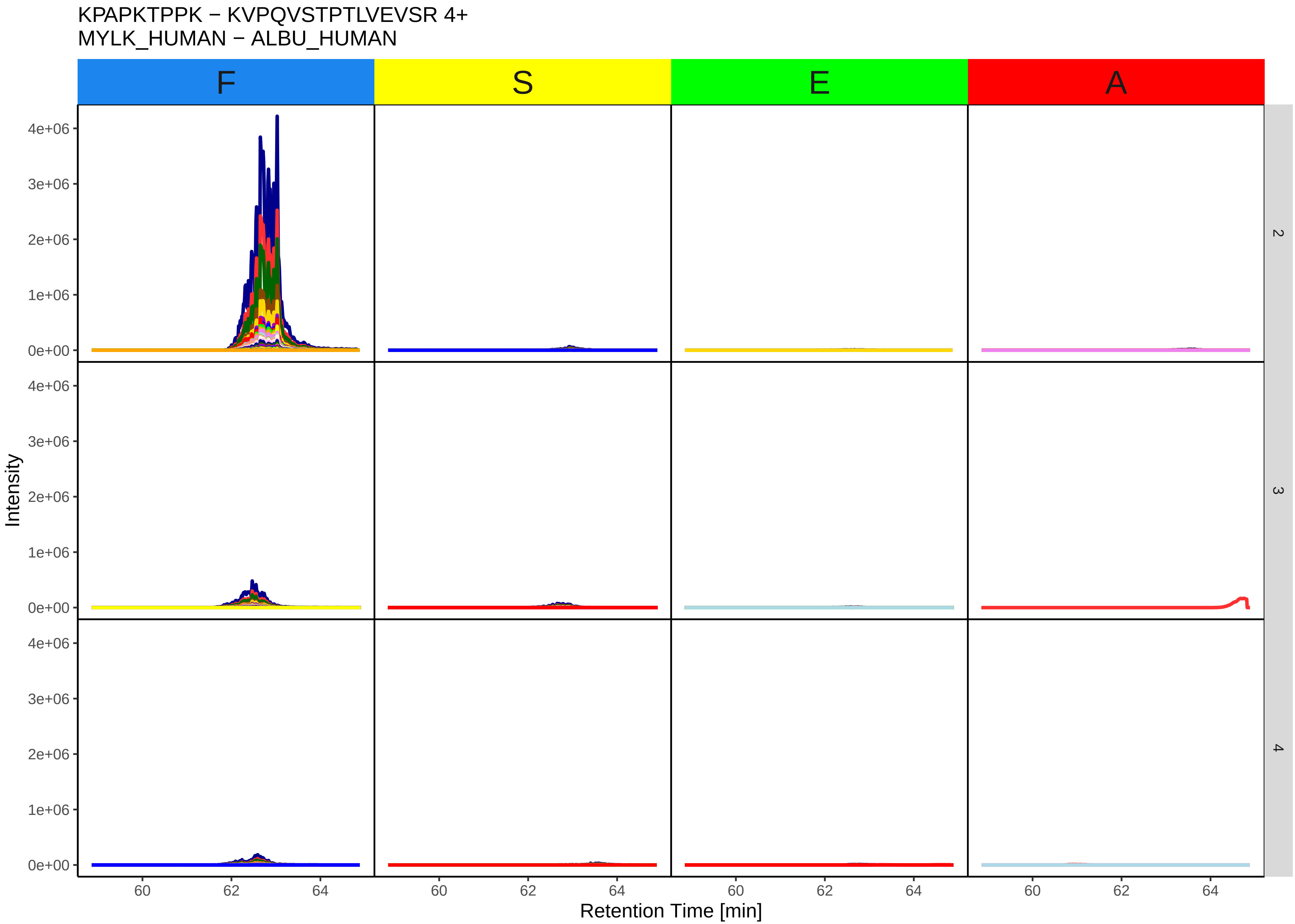
KPAPKTPPK − KVPQVSTPTLVEVSR 4+
MYLK_HUMAN − ALBU_HUMAN
F
S
E
A
2
3
4
Intensity
4e+06
3e+06
2e+06
1e+06
0e+00
60
62
64
Retention Time [min]

DLKDFIR − IGQLSEKEIQK 5+
KCNH8_HUMAN − NEXN_HUMAN

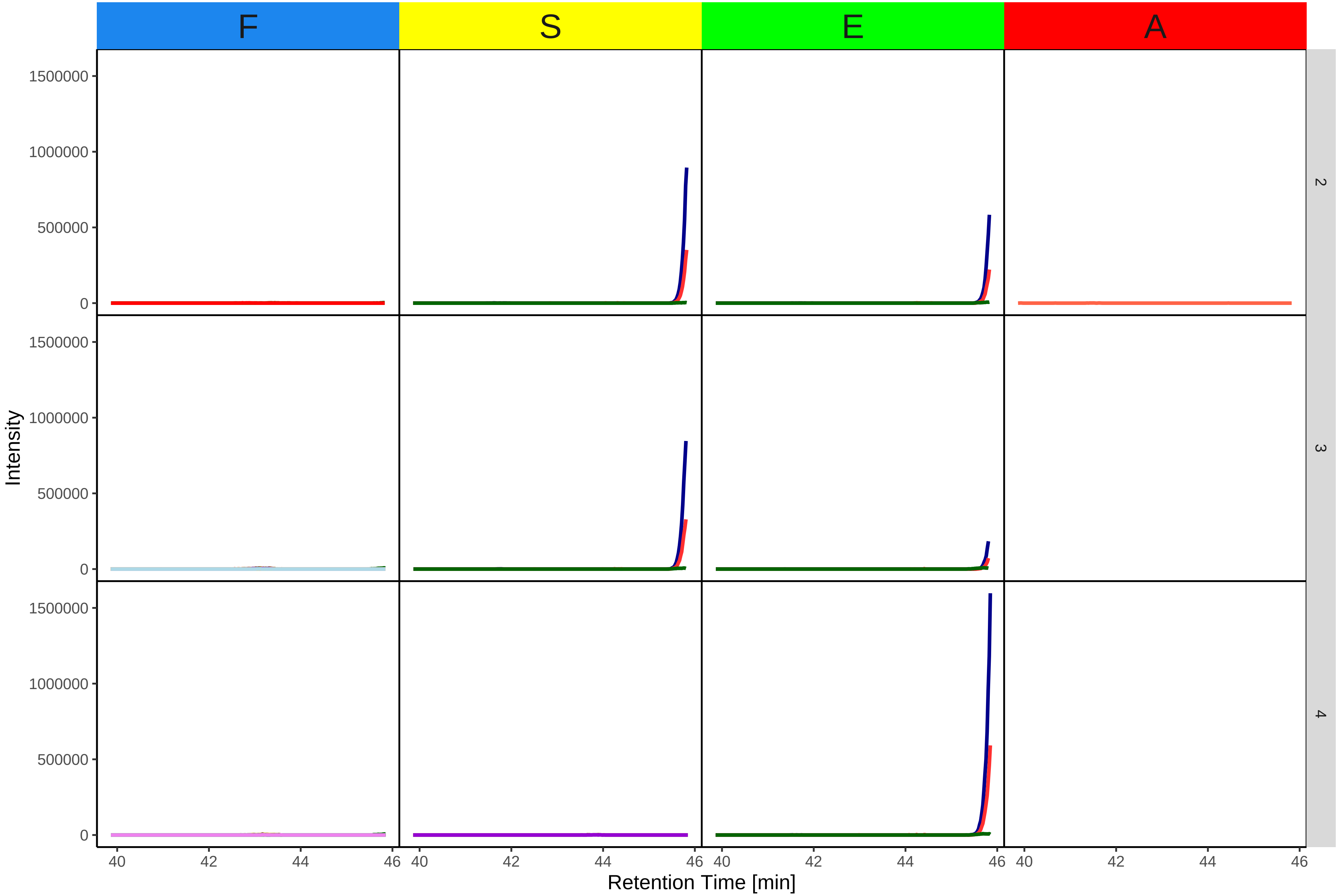

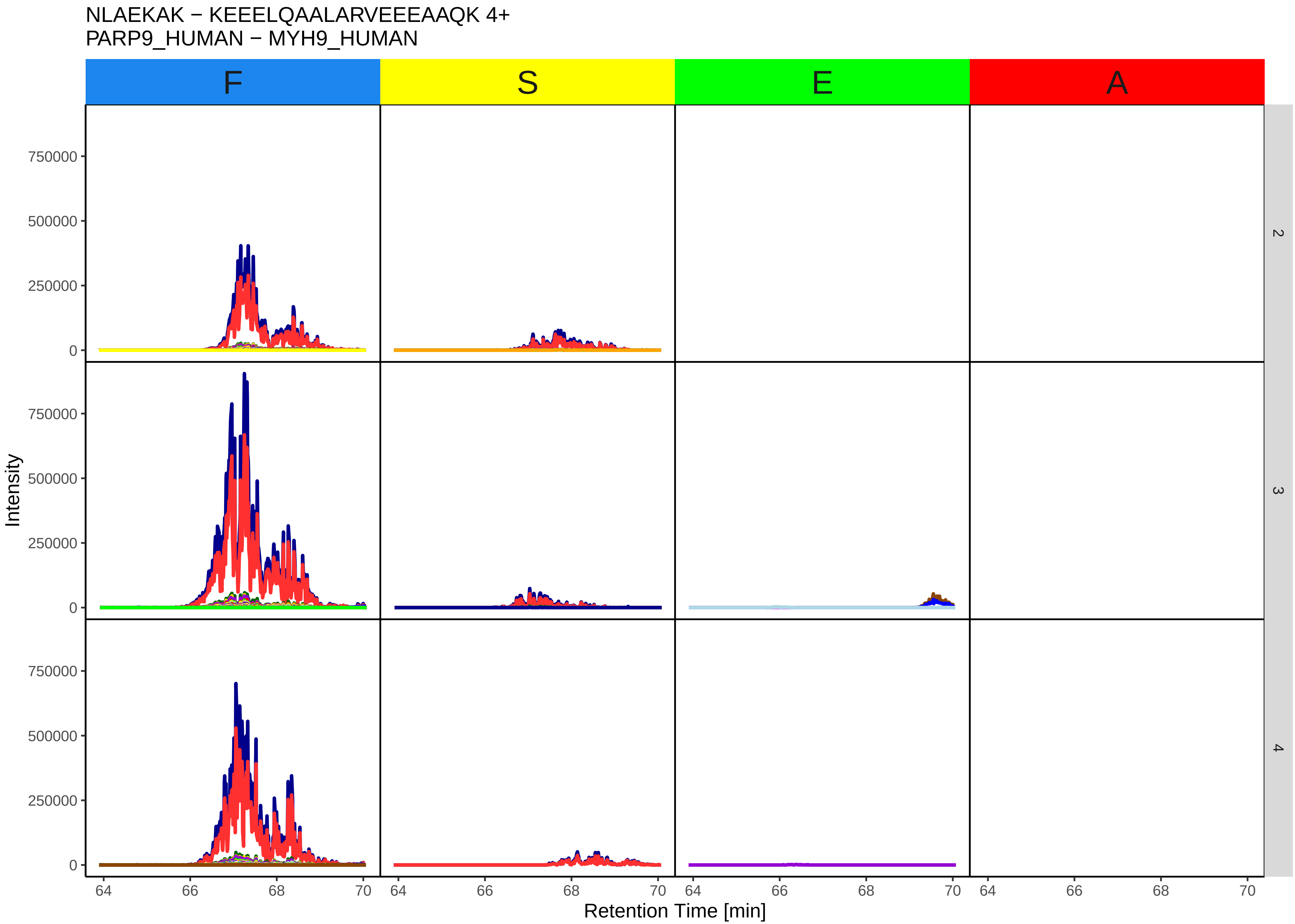

NLAEKAK − KEEELQAALARVEEEAAQK 4+
PARP9_HUMAN − MYH9_HUMAN
F
S
E
A
2
3
4
Intensity
750000
500000
250000
0
64
66
68
70
Retention Time [min]

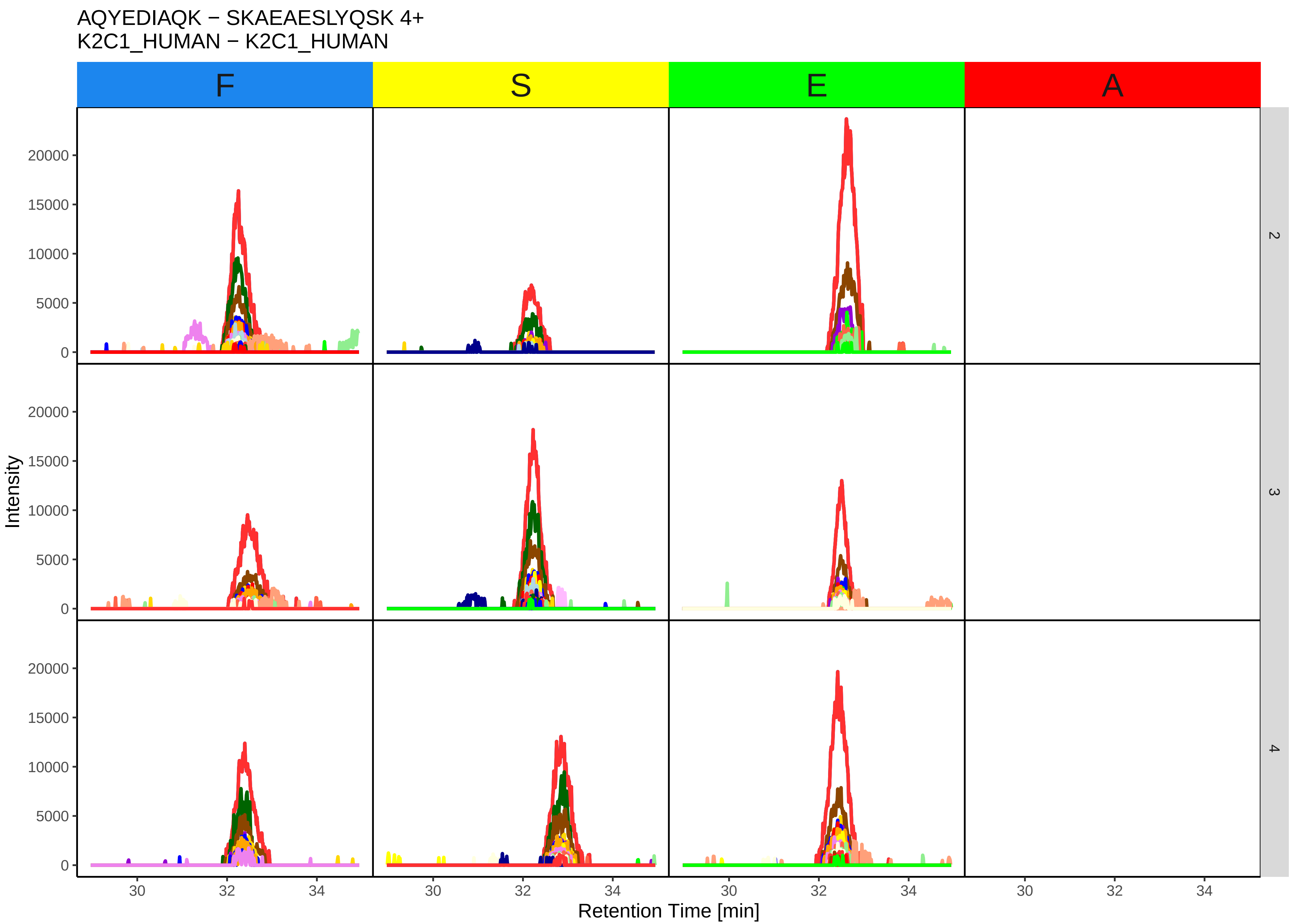

AQYEDIAQK − SKAEAESLYQSK 4+
K2C1_HUMAN − K2C1_HUMAN
F
S
E
A
2
3
4
Intensity
0
5000
10000
15000
20000
30
32
34
Retention Time [min]

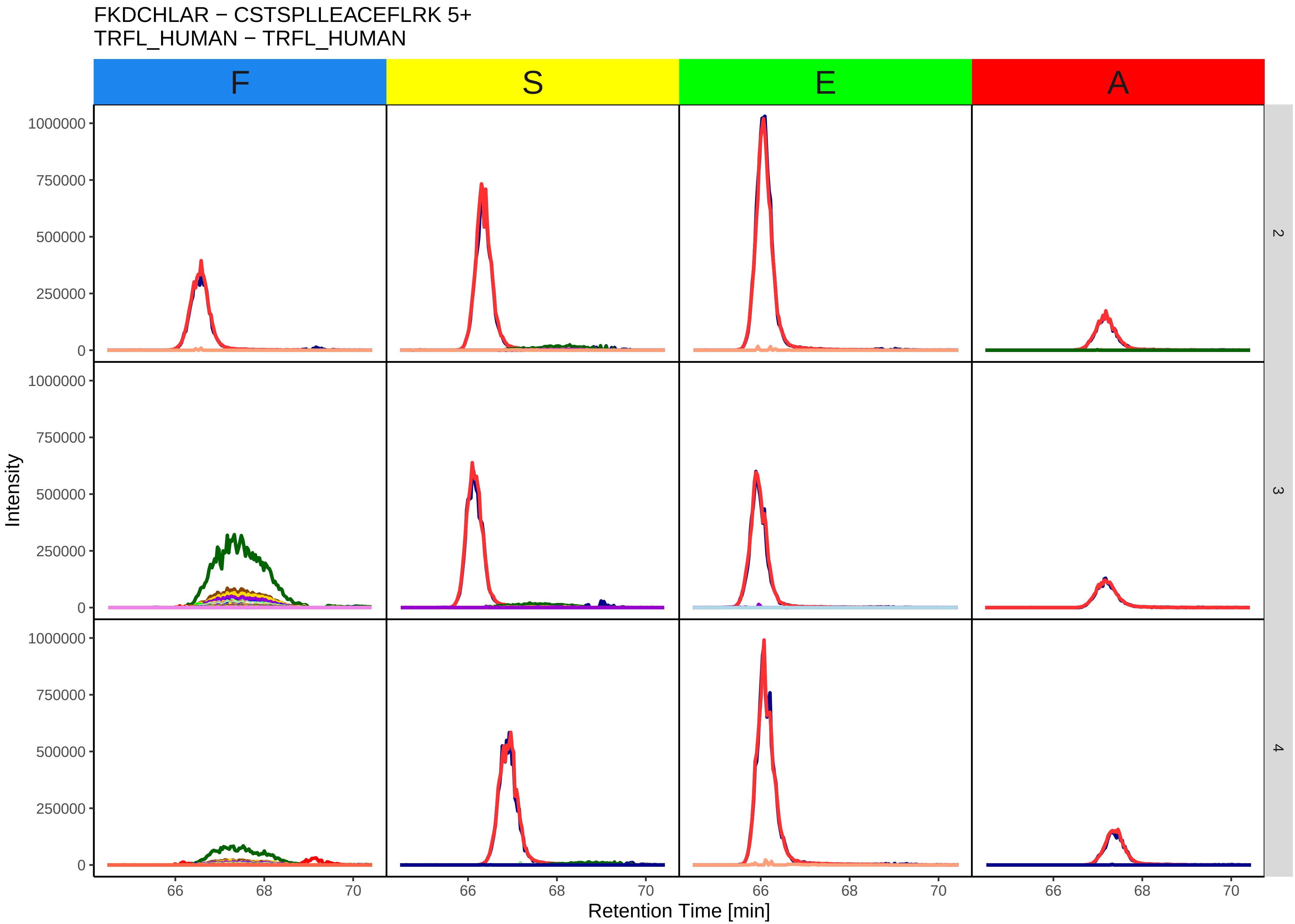
FKDCHLAR − CSTSPLLEACEFLRK 5+
TRFL_HUMAN − TRFL_HUMAN
F
S
E
A
2
3
4
Intensity
1000000
750000
500000
250000
0
66
68
70
Retention Time [min]

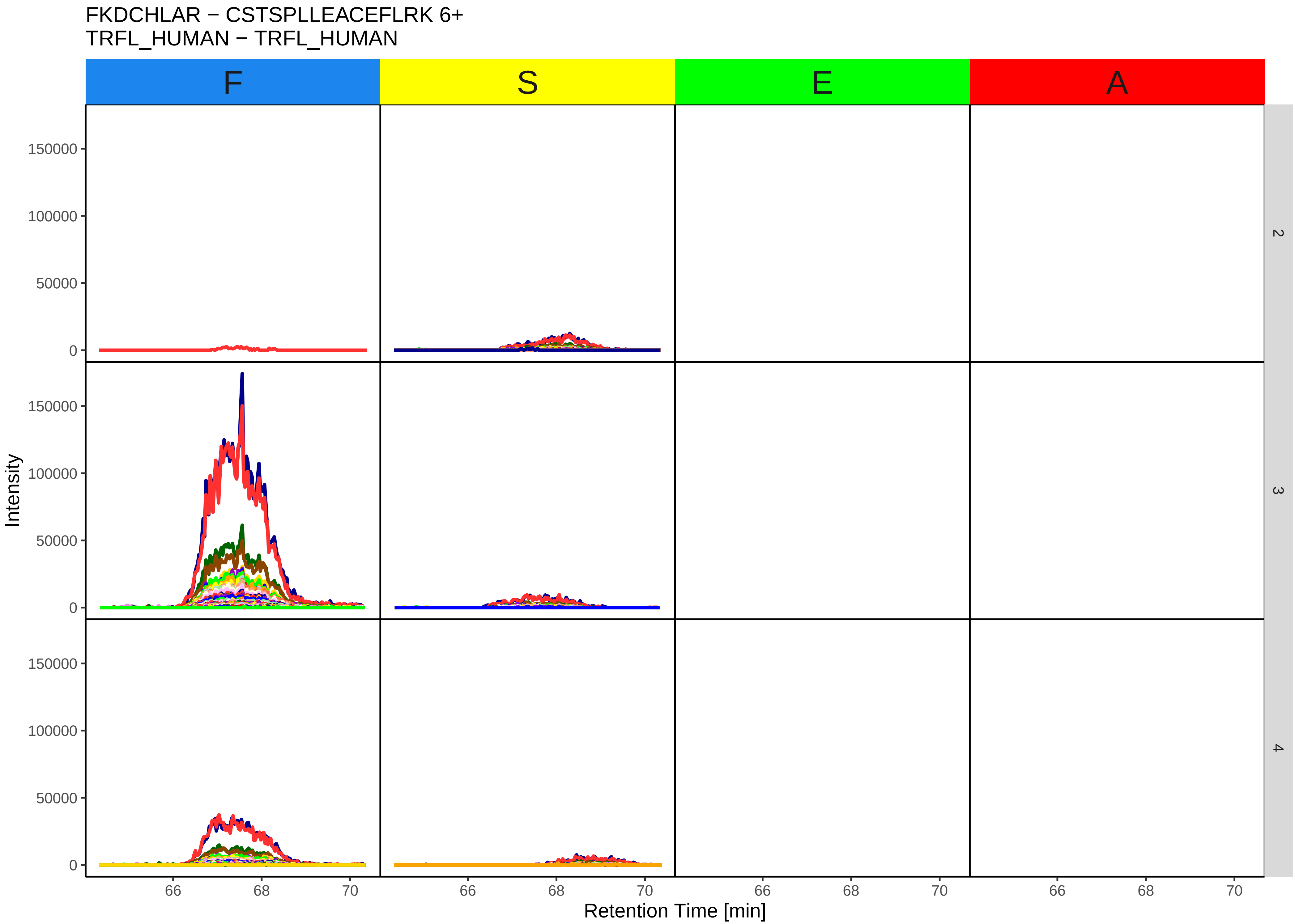
FKDCHLAR − CSTSPLLEACEFLRK 6+
TRFL_HUMAN − TRFL_HUMAN
F
S
E
A
2
3
4
Intensity
150000
100000
50000
0
66
68
70
Retention Time [min]
