# Supplementary material for: Mass Spectrometric Detection of Formaldehyde-Crosslinked PBMC Proteins in Cell-Free DNA Blood Collection Tubes
Source: Molecules. 2023 Nov 30;28(23):7880. doi: 10.3390/molecules28237880 (PMC10708122; doi:10.3390/molecules28237880)
Supplement: Supplementary file 1 [file molecules-28-07880-s001.zip › Summary of Supplemental Materials.docx]

**Summary of Supplemental Materials for:**

**Mass Spectrometric Detection of Formaldehyde-crosslinked PBMC Proteins in Cell-Free DNA Blood-Collection Tubes**

Daniel Röth^1^, Jessica Molina- Franky^1,2,3^, John C. Williams^4^, and Markus Kalkum^1,^ *

^1^ Department of Immunology & Theranostics, Arthur Riggs Diabetes and Metabolism Research Institute, Beckman Research Institute of the City of Hope, Duarte, CA 91010, USA

^2^ Molecular Biology and Immunology Department, Fundación Instituto de Inmunología de Colombia (FIDIC), Bogotá 112111, Colombia

^3^ Biotechnology, Faculty of Sciences, Universidad Nacional de Colombia, Bogotá 111321, Colombia

^4^ Department of Cancer Biology and Molecular Medicine, Beckman Research Institute of the City of Hope, Duarte, CA 91010, USA

**S1: S1_All_FAXL_over_1.5.xlsx**

Excel spread sheet summarizing the output from Formaldehyde_XL_Analyzer software from the Kalisman lab (http://biolchem.huji.ac.il/nirka/index.html) using three different protein FASTA databases (detected, HPA, membrane).

See: http://biolchem.huji.ac.il/nirka/Software/Formaldehyde_2020/Read_Me.pdf

**S2: S2_ XICs_in_Heatmap_order.pdf**

Extracted ion chromatograms for the validation of all potential crosslinks in S samples. XICs for F, S, E, and A samples are shown for donors 2, 3, and 4. Each colored chromatogram represents one transition to a b or y ion.
